# Highly Enantiomerically Enriched Secondary Alcohols via Epoxide Hydrogenolysis

**DOI:** 10.1021/acs.organomet.4c00214

**Published:** 2024-06-17

**Authors:** Olivia J. Borden, Benjamin T. Joseph, Marianna C. Head, Obsidian A. Ammons, Diane Eun Kim, Abigail C. Bonino, Jason M. Keith, Anthony R. Chianese

**Affiliations:** Department of Chemistry, Colgate University, 13 Oak Drive, Hamilton, New York 13346, United States

## Abstract

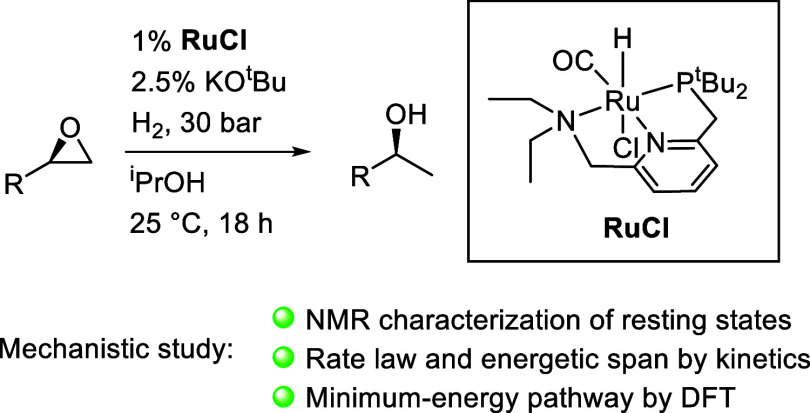

In this article, we report the development of ruthenium-catalyzed hydrogenolysis of epoxides to selectively give the branched (Markovnikov) alcohol products. In contrast to previously reported catalysts, the use of Milstein’s PNN-pincer-ruthenium complex at room temperature allows the conversion of enantiomerically enriched epoxides to secondary alcohols without racemization of the product. The catalyst is effective for a range of aryl epoxides, alkyl epoxides, and glycidyl ethers and is the first homogeneous system to selectively promote hydrogenolysis of glycidol to 1,2-propanediol, without loss of enantiomeric purity. A detailed mechanistic study was conducted, including experimental observations of catalyst speciation under catalytically relevant conditions, comprehensive kinetic characterization of the catalytic reaction, and computational analysis via density functional theory. Heterolytic hydrogen cleavage is mediated by the ruthenium center and exogenous alkoxide base. Epoxide ring opening occurs through an opposite-side attack of the ruthenium hydride on the less-hindered epoxide carbon, giving the branched alcohol product selectively.

## Introduction

**Scheme 1:**
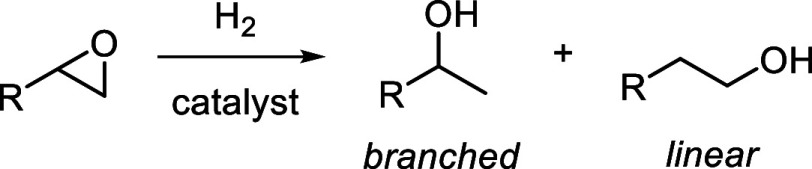
Catalytic Epoxide Hydrogenolysis

The homogeneous transition-metal-catalyzed hydrogenolysis of epoxides, first reported by Ikariya and co-workers in 2003,^1^ has recently emerged as a method for the selective synthesis of a variety of substituted primary, secondary, and tertiary alcohols (Scheme 1). Several reported catalysts selectively give the linear (anti-Markovnikov) alcohol isomer. For example, Scheuermann and co-workers described a PCP-pincer-iridium/triflic acid catalyst system,^2^ which they later showed operates via initial acid-catalyzed hydrolysis of the epoxide, followed by hydrogenolysis to the terminal alcohol catalyzed by iridium nanoparticles generated in situ.^3^ Yao et al. disclosed a titanium–cobalt dual-catalyst system and provided evidence for a radical-based activation of H_2_ and transfer to epoxide substrates.^4^ Beller and co-workers reported an iron/trifluoroacetic acid system^5^ and a cobalt/zinc triflate system,^6^ both of which operate through initial Meinwald rearrangement of the epoxide to the aldehyde, followed by metal-catalyzed hydrogenation to the primary alcohol.

**Chart 1:**
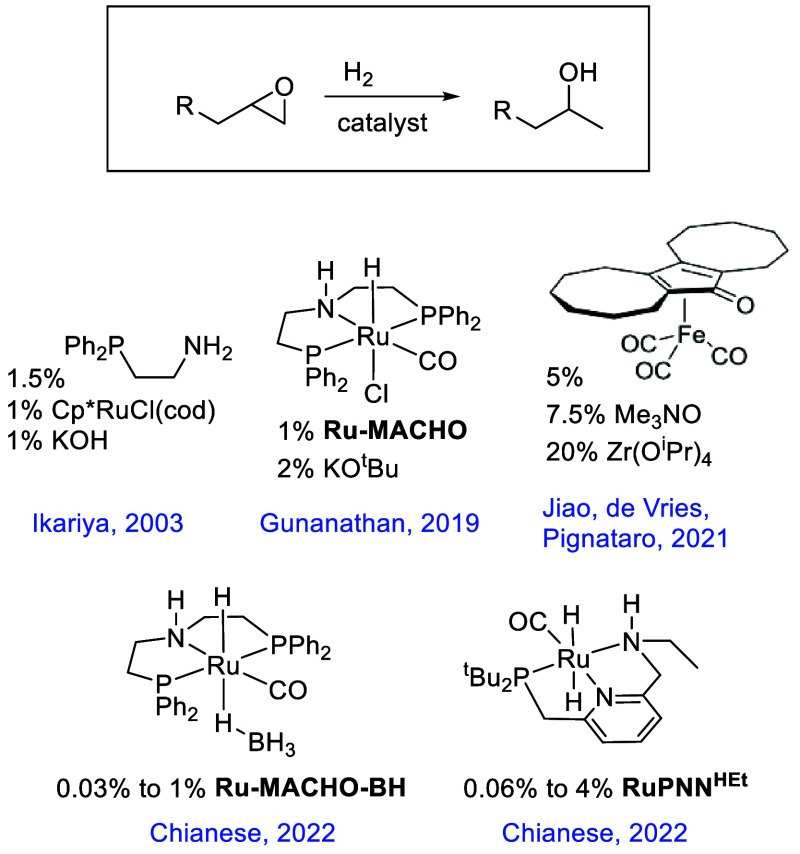
Previously Reported Catalysts for Branched-Selective Epoxide Hydrogenolysis

On the other hand, several catalysts selectively produce the branched (Markovnikov) alcohol isomer. So far, all reported catalysts in this category are capable of Noyori-type^7^ metal–ligand cooperation, involving either RuH/NH or FeH/OH moieties (Chart 1). Ikariya’s 2003 report^1^ featured a combination of Cp*RuCl(1,5-cyclooctadiene), PPh_2_CH_2_CH_2_NH_2_, and KOH. Gunanathan reported that the commercially available Ru-MACHO, in combination with KO^*t*^Bu, promotes the selective hydrogenolysis of a variety of substituted epoxides to give the Markovnikov product.^8^ In both cases, the authors proposed that the epoxide ring opens through a Noyori-type concerted transfer of Ru–H and N–H to the epoxide C and O atoms, respectively. Tadiello et al. showed that a Knölker-type iron-cyclopentadienone catalyst selectively gives the linear product if Al(OTf)_3_ is added as a cocatalyst, while the branched product is favored with Zr(O^i^Pr)_4_ as a cocatalyst.^9^ Based on density functional theory (DFT) calculations, these authors proposed a competition between a Noyori-type concerted pathway and an initial Meinwald isomerization to the aldehyde followed by aldehyde hydrogenation.

Notably, none of the above studies describe the synthesis of enantiomerically enriched alcohols via the hydrogenolysis of enantiomerically enriched epoxides. Ikariya and co-workers noted in a 2007 review^10^ that racemization of the secondary alcohol products was rapid and prevented the application of epoxide hydrogenolysis to the synthesis of enantiomerically enriched secondary alcohols. Gunanathan reported that attempted hydrogenolysis of (*R*)-glycidol gave a complex mixture of products and did not describe other attempts with enantiomerically enriched substrates.^8^

In 2022, we reported^11^ that two Noyori-type ruthenium catalysts, the commercially available **Ru-MACHO-BH** and **RuPNN**^**HEt**^, formed from Milstein’s catalyst^12^ by ethane loss and hydrogen addition,^13^ are highly active for the branched-selective hydrogenolysis of epoxides, without the requirement of strongly basic or Lewis-acidic cocatalysts (Chart 1). High yields were obtained at catalyst loadings as low as 0.03%, compared with 1^1,8^ or 5%^9^ loading in prior reports. Through monitoring of the reactant and product e.e. over the course of the reaction, we showed that product racemization is rapid under the catalytic conditions, which prevented the application of this method for the synthesis of enantiomerically enriched alcohols from epoxides.

**Scheme 2:**
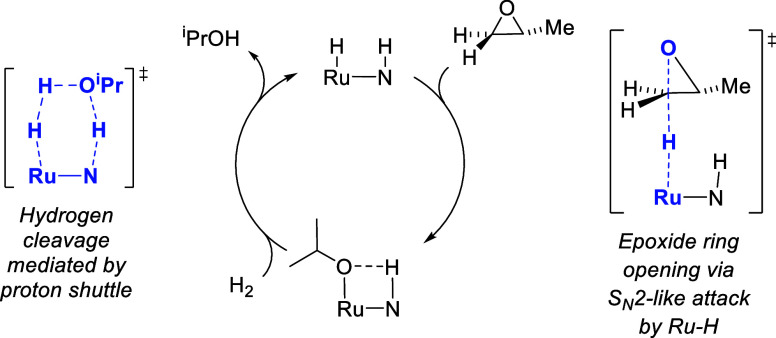
Abbreviated Mechanism for Branched-Selective Epoxide Hydrogenolysis Catalyzed by Noyori-Type Ruthenium-Pincer Complexes

In 2023, we completed a combined experimental/computational mechanistic study of the **Ru-MACHO-BH** and **RuPNN**^**HEt**^ catalysts for epoxide hydrogenolysis.^14^ For both catalysts, we showed that the previously proposed^1,8,9^ concerted, Noyori-type mechanisms for hydrogen transfer to the epoxide have implausibly high free energy barriers more than 50 kcal/mol. Instead, epoxide ring opening proceeds through an S_N_2-like opposite-side attack of the ruthenium hydride on the less-substituted epoxide carbon, without involvement of the pendant N–H group (Scheme 2, right). Hydrogen activation proceeds by Noyori-type metal–ligand cooperation, assisted by an alcohol functioning as a proton shuttle (Scheme 2, left).

Because product racemization presumably proceeds through reversible dehydrogenation of the secondary alcohol to the ketone via a Noyori-type bifunctional mechanism,^7^ we hypothesized that an analogous complex lacking the pendant N–H group could potentially catalyze epoxide hydrogenolysis while avoiding product racemization. Following an extensive process of screening and optimization, we were pleased to find that the commercially available Milstein’s catalyst, in combination with KO^*t*^Bu or KO^i^Pr in ^i^PrOH, promotes the hydrogenolysis of a range of substituted epoxides at room temperature, with extremely high-branched:linear selectivity and minimal product racemization. This article describes the discovery and optimization of this catalyst system, an exploration of the substrate scope, and a detailed mechanistic study combining computation, kinetics, and spectroscopic analysis of resting-state speciation. We conclude that epoxide ring opening proceeds through an S_N_2-like attack of the ruthenium hydride on the less-hindered epoxide carbon, while heterolytic hydrogen activation is mediated by an exogenous alkoxide base. This article was previously deposited to the preprint server ChemRxiv.^15^

## Results and Discussion

### Catalyst Screening and Optimization

We began our screening process with the following goals: (1) high yields of alcohol products with low catalyst loading under mild conditions; (2) high selectivity for the branched (chiral) product over the linear product; and (3) minimal racemization of the branched product. For catalyst screening, we chose (*R*)-styrene oxide as the model substrate because it is available commercially with 98% e.e. and because achieving high selectivity for the branched product has been challenging with aryl epoxide substrates.^1,8,9,11^ We began by screening a variety of known transition-metal pincer complexes (Ru, Ir, and Mn). We used preformed catalysts instead of an in situ combination of ligand and metal precursor, to avoid potential side reactions arising from incomplete metalation. Because the solvent has been shown to strongly affect both catalyst activity and selectivity in epoxide hydrogenolysis,^11^ we screened catalyst systems in toluene, ^*t*^AmOH, ^i^PrOH, EtOH, and MeOH. Table 1 shows highlighted experiments from this optimization process; Table S1 in the Supporting Information shows the results of all 104 screening experiments conducted.

**Table 1:**
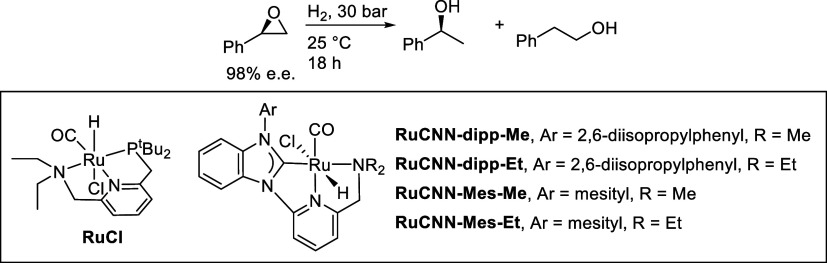
Catalyst Screening and Optimization

entry	catalyst	mol %	additive	mol %	solvent	[epoxide] (M)	yield (%)	e.e. (%)	b:l
1	RuCNN-dipp-Me	1	NaO^*t*^Bu	10	^i^PrOH	0.125	74	92	7.4
2	RuCNN-dipp-Et	1	NaO^*t*^Bu	10	^i^PrOH	0.125	>99	79	7.7
3	RuCNN-Mes-Me	1	NaO^*t*^Bu	10	^i^PrOH	0.125	79	91	5.5
4	RuCNN-Mes-Et	1	NaO^*t*^Bu	10	^i^PrOH	0.125	62	92	5.2
5	RuCl	1	NaO^*t*^Bu	10	^i^PrOH	0.125	31	98	11.1
6	RuCl	1	NaO^*t*^Bu	10	toluene	0.125	2	98	>10
7	RuCl	1	NaO^*t*^Bu	10	^*t*^AmOH	0.125	62	93	19.1
8	RuCl	1	NaO^*t*^Bu	10	EtOH	0.125	3	98	6.7
9	RuCl	1	none		^i^PrOH	0.125	0		
10	RuCl	1	CsF	10	^i^PrOH	0.125	0		
11	RuCl	1	Cs_2_CO_3_	10	^i^PrOH	0.125	24	98	11.5
12	RuCl	1	KF	10	^i^PrOH	0.125	3		0
13	RuCl	1	BEMP	10	^i^PrOH	0.125	4	96	9.5
14	RuCl	1	LiAlH_4_	10	^i^PrOH	0.125	3	98	11.6
15	RuCl	1	NaBH_4_	10	^i^PrOH	0.125	5	93	5.1
16	RuCl	1	KOAc	10	^i^PrOH	0.125	0		
17	RuCl	1	K_3_PO_4_	10	^i^PrOH	0.125	8	98	9.5
18	RuCl	1	KO^*t*^Bu	10	^i^PrOH	0.125	57	98	11.3
19	RuCl	0.25	KO^*t*^Bu	10	^i^PrOH	0.5	36	98	12.2
20	RuCl	1	KO^*t*^Bu	2.5	^i^PrOH	0.5	>99	98	11.9
21	RuCl	1	KO^*t*^Bu	10	^i^PrOH	0.5	>99	98	12
22	RuCl	1	LiO^i^Pr	2.5	^i^PrOH	0.5	52	98	11.9
23	RuCl	1	NaO^i^Pr	2.5	^i^PrOH	0.5	60	98	12.0
24	RuCl	1	KO^i^Pr	2.5	^i^PrOH	0.5	>99	98	11.9

Entries 1–5 show the most promising precatalysts identified in our screening. Notably, all five are ruthenium–pincer complexes, in which the pincer ligand lacks an N–H functional group. Of these complexes, we identified Milstein’s hydridochloride precatalyst **RuCl** (entry 5) as the most promising because it provided the highest branched:linear selectivity and showed no observable product racemization. Switching the solvent from isopropyl alcohol (entry 5) to toluene (entry 6) or ethanol (entry 8) dramatically decreased the product yield. Catalyst activity was improved in *t*-amyl alcohol (entry 7), but some product racemization was observed in this solvent. Continuing with **RuCl** in isopropyl alcohol, we then screened a variety of basic or hydridic additives (entries 9–18). In this series, KO^*t*^Bu (entry 18) emerged as the most promising additive, providing the alcohol product with a 57% yield with high-branched selectivity and no observable product racemization. We then surveyed a range of substrate, catalyst, and base concentrations (entries 19–21) and found the following: (1) higher epoxide concentration is beneficial; (2) 1 mol % loading of **RuCl** is necessary for full conversion; and (3) the loading of KO^*t*^Bu can be lowered to 2.5 mol % with no decrease in yield or selectivity.

The improved yield with KO^*t*^Bu compared to NaO^*t*^Bu (entries 5 vs 18) prompted a comparison of the effect of the alkali metal cation. To avoid complexities arising from multiple alcohols and alkoxide anions in the isopropyl alcohol solvent, we compared the commercially available salts of isopropoxide (entries 22–24). We found that KO^i^Pr was as effective as KO^*t*^Bu (entries 24 vs 20), but that NaO^i^Pr and LiO^i^Pr were less effective, giving similar selectivities but decreased conversion to product. In the end, entries 20 and 24 represent the optimized conditions for this reaction, giving >99% yield of phenethyl alcohol with no observable racemization and a branched:linear ratio of 11.9:1. For practical synthetic applications, KO^*t*^Bu is preferred as the base because of its commercial availability as a solid. KO^i^Pr was employed in our kinetic studies described below, which were conducted in isopropyl alcohol solvent.

### Substrate Scope

With optimized conditions determined for epoxide hydrogenolysis catalyzed by **RuCl** and KO^*t*^Bu, we began to survey the reactivity of a variety of monosubstituted epoxide substrates, to assess whether the catalyst activity, selectivity, and absence of product racemization were maintained (Table 2). First, we compared differently substituted aryl epoxides, which have posed a challenge in the past in obtaining high regioselectivity for the branched product.^1,8,11^ (*R*)-Styrene oxide, the subject of the above optimization study, is reduced with 11.9:1 selectivity for the branched product, with no observable product racemization. *para*-Fluoro, chloro-, and bromo-substituents are all tolerated, and even higher regioselectivity for the branched product is observed. The fluoro- and bromo-substituted epoxides required a higher catalyst loading, 4 and 2% respectively, to achieve full conversion. For the chloro- and bromo-substituted epoxides, a small amount of product racemization were observed, corresponding to 3 and 2% loss of enantiomeric excess, respectively, for these substrates. While we do not know the origin of this small amount of racemization, we note that aryl-halide-containing substrates are challenging for ruthenium-catalyzed epoxide hydrogenolysis, typically requiring higher catalyst loadings for full conversion.^8,11^ This may stem from an unknown transformation of the catalyst through reaction with the substrate, which may contribute to product racemization.

**Table 2:**
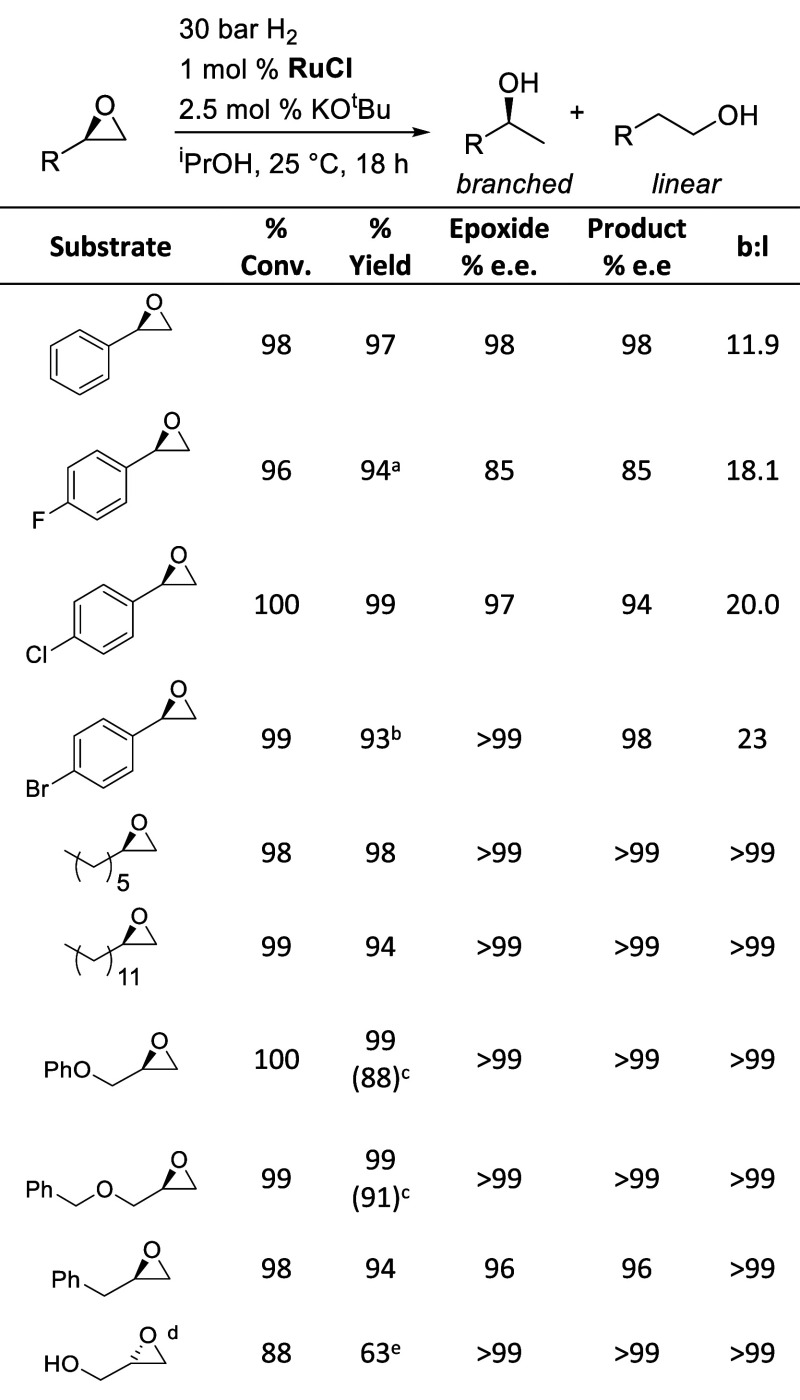
Substrate Scope for Epoxide Hydrogenolysis

a 2 mol % **RuCl** and 5 mol % KO^*t*^Bu were used.

b 4 mol % **RuCl** and 10 mol % KO^*t*^Bu were used.

c Percent yields in parentheses represent isolated yields on a 1.0 g scale.

d (*S*)-Glycidol was used as shown and gave the (*S*)-1,2-propanediol product expected if the configuration of the stereocenter is retained.

e 3.3 mol % **RuCl** and 8.3 mol % KO^*t*^Bu were used.

We then turned to monosubstituted epoxides with a directly attached sp^3^ carbon, which typically give only the branched product in hydrogenolysis catalyzed by Noyori-type complexes.^1,8,11^ The aliphatic epoxides 1-octene oxide and 1-tetradecene oxide were cleanly converted to the secondary alcohols in high yield with no observable linear product and no product racemization. Similar results were obtained for phenyloxy- and benzyloxy-substituted derivatives, as well as allyl benzene oxide. The hydrogenolysis of substrates containing a primary alcohol functional group poses a particular challenge, as base-promoted oligomerization competes with hydrogenolysis at higher temperatures. Ikariya reported no substrates with alcohol functional groups,^1^ and Gunanathan reported that a complex mixture was obtained for the hydrogenolysis of (*R*)-glycidol catalyzed by **Ru-MACHO** and KO^*t*^Bu.^8^ With our system operating at room temperature, (*S*)-glycidol was hydrogenated to give the (*S*)-1,2-propanediol product with no loss of e.e. in 63% yield, albeit with a higher 3.3% loading of **RuCl** required to achieve high conversion.

The high-branched selectivity for this catalyst system likely stems from much slower reactivity at more highly substituted epoxide positions. Indeed, the 1,2-disubstituted substrate *trans*-stilbene oxide is unreactive under our optimized conditions. Although this method tolerates a substrate containing primary alcohol as described above, limitations were observed for other functional groups. In the attempted hydrogenolysis of allyl glycidyl ether, a complex mixture of products was obtained due to competitive hydrogenation of the C=C double bond. In the attempted hydrogenolysis of the ester-containing glycidyl methacrylate, a mixture of products was observed due to known base-promoted transesterification.^16^

### Mechanistic Study: Background

**Scheme 3:**
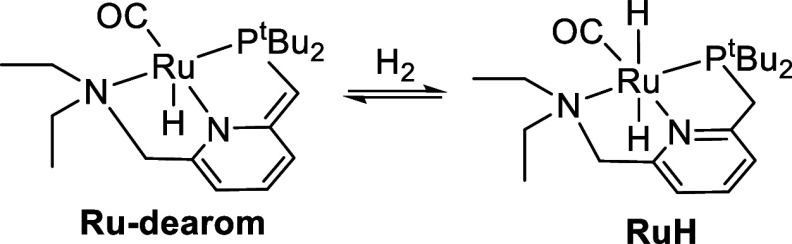
Reversible Activation of Hydrogen by **Ru-dearom**

Milstein’s catalyst precursor **RuCl**—and the deprotonated, dearomatized form **Ru-dearom**—have a rich history of application in the hydrogenation and dehydrogenation of polar bonds,^17^ following Milstein’s initial reports of ester hydrogenation^18^ and the reverse reaction, the acceptorless dehydrogenative coupling of primary alcohols to esters.^12^**Ru-dearom** is known to reversibly activate dihydrogen at room temperature to give the dihydride **RuH** (Scheme 3).^18^ This metal–ligand-cooperative cleavage of hydrogen (or its reverse in dehydrogenation reactions) was featured in many DFT studies of the reactions catalyzed by **RuH** or **Ru-dearom**,^19^ but experimental studies of catalyst speciation under operating conditions (≥100 °C) were not reported.

**Scheme 4:**
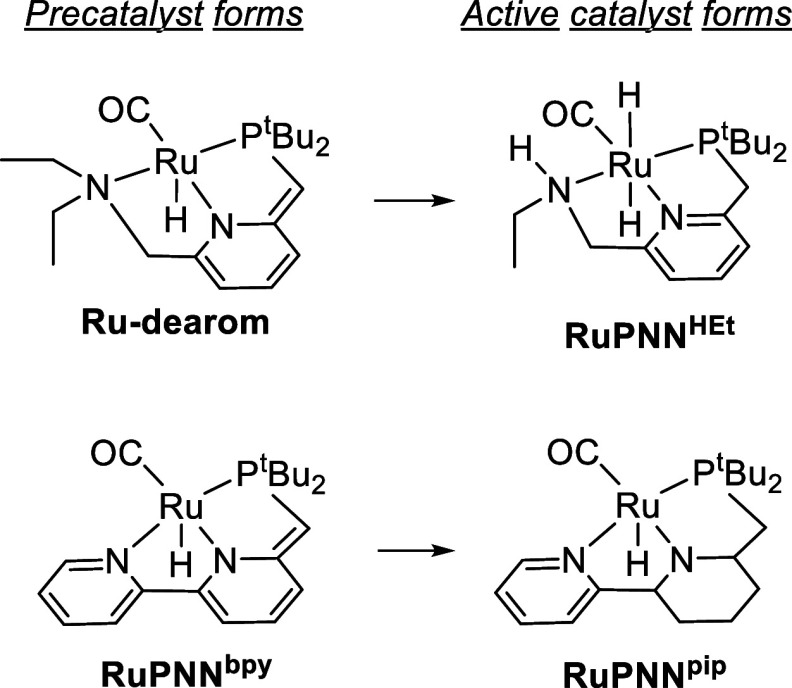
Conversion of Precatalysts with Dearomatized Pincer Ligands to the Active, Noyori-Type Forms

In 2019, we demonstrated that **Ru-dearom** is catalytically inactive for ester hydrogenation but rapidly undergoes a dehydroalkylation reaction under the conditions of catalysis, releasing ethane and ultimately producing **RuPNN**^**HEt**^,^13^ which operates through a well-precedented Noyori-type mechanism requiring the nascent N–H functional group (Scheme 4, top).^20^ Khaskin and Gusev later showed that the closely related **RuPNN**^**bpy**^ also forms a Noyori-type catalyst **RuPNN**^**pip**^ under operating conditions, this time through hydrogenation of the pyridine ring (Scheme 4, bottom).^21^ In 2020, Gusev showed through DFT that for Milstein’s catalyst, hydrogen activation mediated by an exogenous alkoxide ion proceeds with a lower barrier than activation through the CH_2_ linker.^22^ Taken together, these recent reports call into question the involvement of aromatization/dearomatization pathways through the CH_2_ linkers in catalysis for complexes, such as **Ru-dearom** and **RuPNN**^**bpy**^, and emphasize that DFT calculations and studies of stoichiometric reactivity at low temperature, while informative, are most reliable when paired with experimental characterization under catalytically relevant conditions.

For the present catalytic transformation, dehydroalkylative catalyst activation (Scheme 4, top) can be excluded because the reaction is conducted at 25 °C, in which dehydroalkylation is known to be extremely slow.^13^ Although **RuPNN**^**HEt**^, the product of dehydroalkylative activation of **Ru-dearom**, is known to catalyze epoxide hydrogenolysis, it also rapidly catalyzes product racemization,^11^ which is not observed in this work. Based on Gusev’s recent report, we expected that the most energetically accessible pathway for hydrogen activation would involve deprotonation of ruthenium-coordinated H_2_ by exogenous alkoxide.^22^ We anticipated that the preferred pathway for epoxide ring opening by **RuH** would involve an S_N_2-like attack of the ruthenium hydride on the less-hindered epoxide carbon, as we previously demonstrated for the closely analogous complex **RuPNN**^**HEt**^.^14^ To test these hypotheses while considering plausible alternatives, we employed a combination of spectroscopic analysis of catalyst speciation under catalytically relevant conditions, kinetic analysis, and DFT calculations.

### Analysis of Catalyst Resting Speciation

**Scheme 5:**
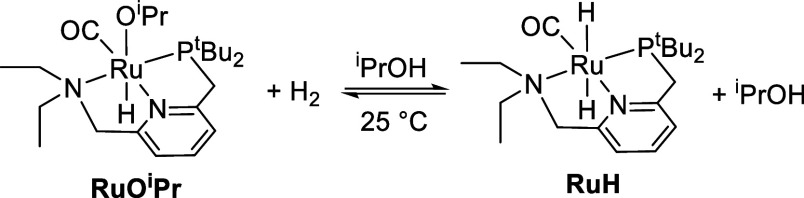
Equilibrium between **RuO**^**i**^**Pr** and **RuH**

We began by studying the speciation of **RuCl**, activated with KO^t^Bu, by NMR spectroscopy in isopropyl alcohol solvent under varying pressures of H_2_. In the absence of hydrogen, the hydridoalkoxide species **RuO**^**i**^**Pr** is formed cleanly, in agreement with previous reports.^22,23^ Under hydrogen, a rapid equilibrium is established between **RuO**^**i**^**Pr** and **RuH** (Scheme 5).

**Figure 1:**
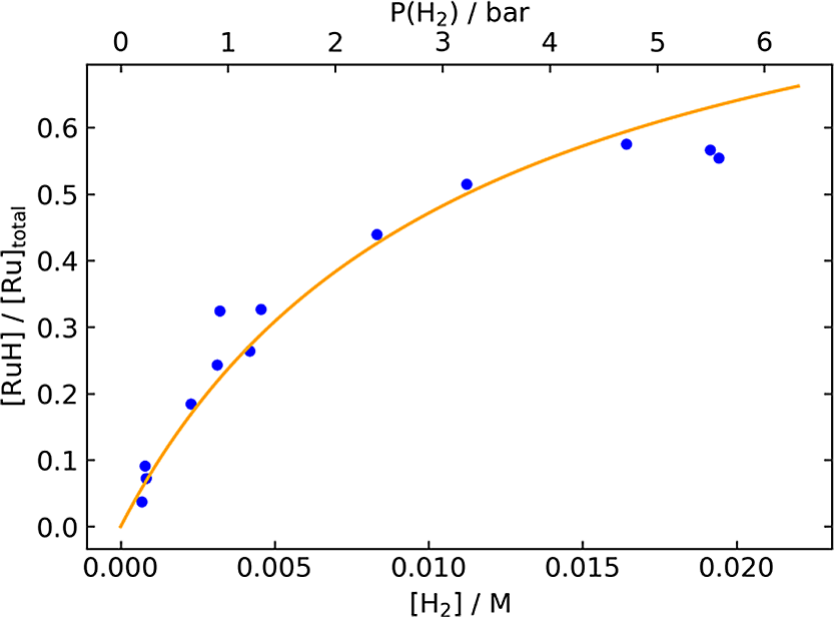
Mole fraction [**RuH**]/[Ru]_total_ vs [H_2_], as determined by ^1^H NMR spectroscopy. Blue points represent values measured in independent experiments. The orange curve represents the best fit of the data to determine the equilibrium constant *K*_1_.

Figure 1 shows the mole fraction [**RuH**]/[Ru]_total_ as a function of the hydrogen concentration, as measured by ^1^H NMR spectroscopy in nondeuterated isopropyl alcohol at 25 °C. A least-squares fit gives *K*_1_ = 89 ± 6, corresponding to Δ*G*° = −2.66 ± 0.04 kcal/mol (see the SI for details). The addition of 0.25 M tetradecene oxide did not produce any new ruthenium species under these conditions or alter the observed ratios, providing evidence against the involvement of additional species with a ruthenium- or ligand-bound epoxide.^24^

**Scheme 6:**
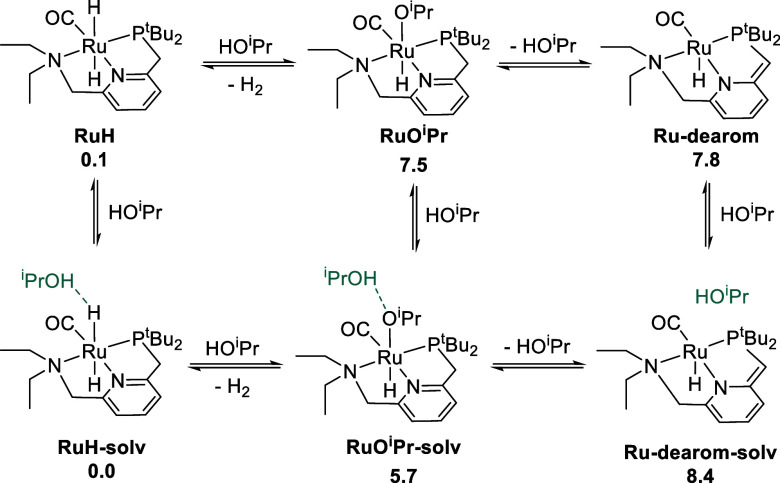
Relative Standard-State Free Energies of Plausible Catalyst Resting States, Calculated by DFT at 298.15 K

Using DFT, we calculated the free energies of the potential resting states **RuO**^**i**^**Pr**, **RuH**, and **Ru-dearom**, in the presence and absence of explicit isopropyl alcohol solvent molecules. Scheme 6 shows the calculated relative free energies. **RuH** and **Ru-dearom** show a very small effect of explicit solvent (≤0.6 kcal/mol), but **RuO**^**i**^**Pr** shows a greater effect, as **RuO**^**i**^**Pr-solv** is 1.9 kcal/mol lower than **RuO**^**i**^**Pr**. This is consistent with previous studies^14,20^ and results from the strong hydrogen-bond-accepting ability of the coordinated alkoxide oxygen in **RuO**^**i**^**Pr**. The calculated standard-state free energy change of −5.7 kcal/mol for the conversion of **RuO**^**i**^**Pr** to **RuH** agrees well with the experimentally measured value of −2.66 kcal/mol. The absence of **Ru-dearom** in these experiments is qualitatively consistent with its higher standard-state free energy calculated by DFT.

### Minimum Energy Pathway

**Figure 2:**
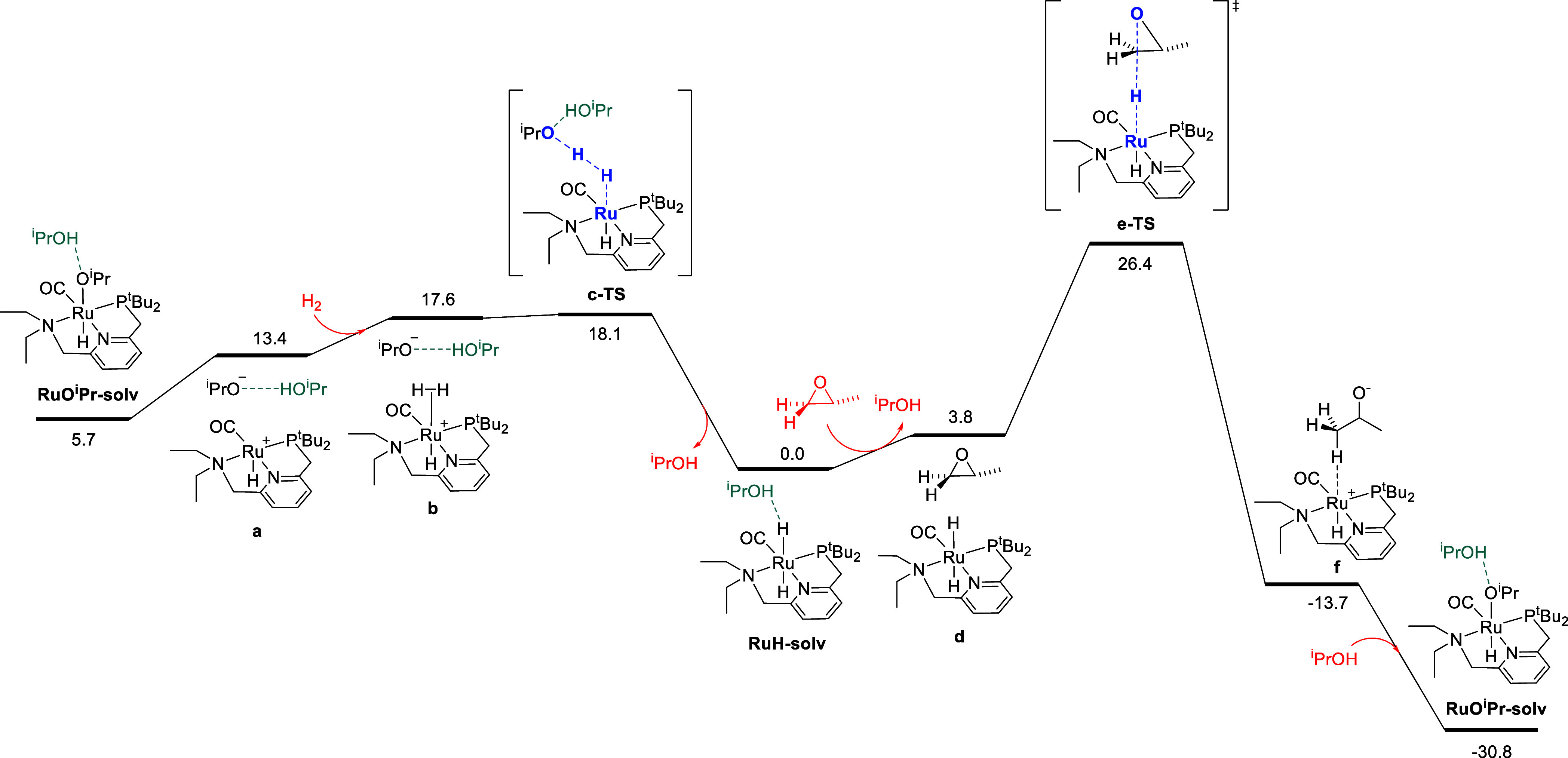
Minimum energy pathway for epoxide hydrogenolysis beginning with **RuO**^**i**^**Pr-solv**. Atoms in bold and blue represent the atoms primarily involved in bond-breaking and bond-forming in transition states. The energies reported are Gibbs free energies at 298.15 K, corrected to the 1.0 M standard state for all species except for the solvent isopropyl alcohol, whose standard state is 13.08 M, its neat molarity. Mass balance is ensured throughout, and energies are calculated relative to **RuH-solv**, the main catalyst resting state.

With experimental confirmation of the resting-state speciation calculated by DFT, we turned to elucidate the pathways for hydrogen activation and epoxide ring opening. To appropriately model the steric and electronic nature of our experimental model substrate, 1-tetradecene oxide, we chose propylene oxide as the model substrate for computations. This has the benefit of minimizing complications due to the multiple conformations of the alkyl chain in 1-tetradecene oxide. Figure 2 shows the calculated minimum energy pathway for the hydrogenolysis of propylene oxide to isopropyl alcohol, beginning with **RuO**^**i**^**Pr-solv**. First, alkoxide dissociation to give **a** is followed by H_2_ coordination to give the σ-complex **b**. This species is deprotonated by exogenous isopropoxide in a nearly barrierless reaction through **c-TS**, which generates the predominant resting-state **RuH-solv**. This isopropoxide-mediated hydrogen activation reaction closely follows the ethoxide-mediated pathway previously reported by Gusev.^22^**RuH-solv** then forms the dispersion adduct **d**, after which epoxide ring opening proceeds through **e-TS**, representing an S_N_2-like attack of the ruthenium hydride on the terminal epoxide carbon. This leads directly to the C–H σ-complex **f**, which rearranges to regenerate **RuO**^**i**^**Pr-solv**. The epoxide ring-opening pathway from **RuH-solv** to **RuO**^**i**^**Pr-solv** is analogous to that calculated previously for the very similar complex **RuPNN**^**HEt**^.^14^

Because **e-TS** features a developing negative charge on the epoxide oxygen, we modeled this pathway including an explicit molecule of isopropyl alcohol to stabilize this negative charge through hydrogen bonding. This pathway, shown in Figure S15, had a slightly higher barrier of 26.7 kcal/mol. In contrast, the activation of hydrogen via **c-TS** does require the explicit isopropyl alcohol molecule shown in Figure 2; the analogous pathway without explicit solvent proceeded through a transition-state **k-TS** that was 6.2 kcal/mol higher in free energy (Figure S16). As hydrogen activation involving the pincer CH_2_ linkers has featured prominently in previous DFT studies on Milstein’s catalyst,^19b,19d,19e,19g,19h,25^ we considered these pathways as alternatives to the MEP shown in Figure 2. We located H_2_ activation transition states involving either **Ru-dearom** or its isomer where the NCH_2_ linker is deprotonated, in both cases including an isopropyl alcohol molecule as a proton shuttle. These pathways, shown in Figures S17 and S18 in the Supporting Information, proceed through barriers at least 7.4 kcal/mol higher than the alkoxide-mediated mechanism through **c-TS**.

### Predicted Kinetics

**Scheme 7:**
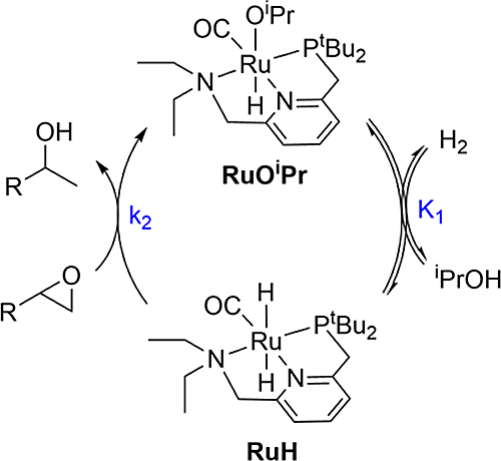
Simplified Mechanism Determining the Kinetics for Epoxide Hydrogenolysis

For the MEP shown in Figure 2, the reaction kinetics can be simplified as shown in Scheme 7. The hydridoalkoxide **RuO**^**i**^**Pr** and the dihydride **RuH** first establish a rapid pre-equilibrium, which is followed by rate-limiting epoxide ring opening through **e-TS**.

As derived in the Supporting Information, this scheme leads to the following rate law:
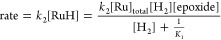


The rate is expected to vary in a first-order manner with the total ruthenium concentration and the epoxide concentration. The dependence of the rate on the hydrogen pressure is expected to follow saturation kinetics, exhibiting first-order dependence at low hydrogen pressure and zero-order dependence at higher pressure. In the kinetics experiments described in the following section, the hydrogen pressures employed range from 10 to 30 bar. Based on the experimentally measured *K*_1_ value of 89 (see above), this leads to predicted mole fractions [**RuH**]/[Ru]_total_ ranging from 0.76 to 0.90. Because Scheme 7 pre-equilibrium has already shifted mostly toward **RuH** under these conditions, only a modest effect of P_H2_ on the catalytic rate is expected.

### Kinetic Studies

We then sought to experimentally determine the effects of reactant and catalyst concentrations on the reaction rate, to compare with the predictions from computation. For kinetic studies, we monitored the hydrogenolysis of racemic 1-tetradecene oxide with varying concentrations of epoxide, ruthenium, and base, as well as varying hydrogen pressure. We chose 1-tetradecene oxide as the substrate for several reasons: (1) the reaction is very clean, as branched 2-tetradecanol is the only observed product; (2) low volatility of the reactant and product facilitate accurate quantitation; and (3) it is sterically very similar to propylene oxide, which was used in the computational studies as described above. We used KO^i^Pr as base rather than KO^*t*^Bu, to avoid potential complications resulting from mixtures of alcohols and alkoxide anions in solution.

**Scheme 8:**
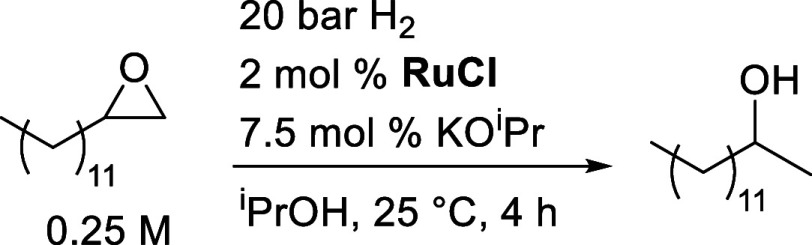
Standard Conditions for Kinetic Experiments

In the standard experiment, 1-tetradecene oxide (0.25 M), **RuCl** (0.005 M), and KO^i^Pr (0.0188 M) were stirred at 25 °C for 4 h under 20 bar of hydrogen, and the reaction progress was monitored by gas chromatography (Scheme 8). In all kinetic experiments, the epoxide was consumed in a pseudo-first-order manner after an induction period of approximately 15–30 min (see Table S5 for complete data). At this point, we do not have a clear explanation for these brief induction periods, but we suspect that they may arise from the heterogeneous nature of the activation of **RuCl** by KO^i^Pr, which results in precipitation of KCl. In the plots below, *k*_obs_ is calculated from the slope of the plot of ln[epoxide] vs time, excluding data from the first 30 min of reaction.

**Figure 3:**
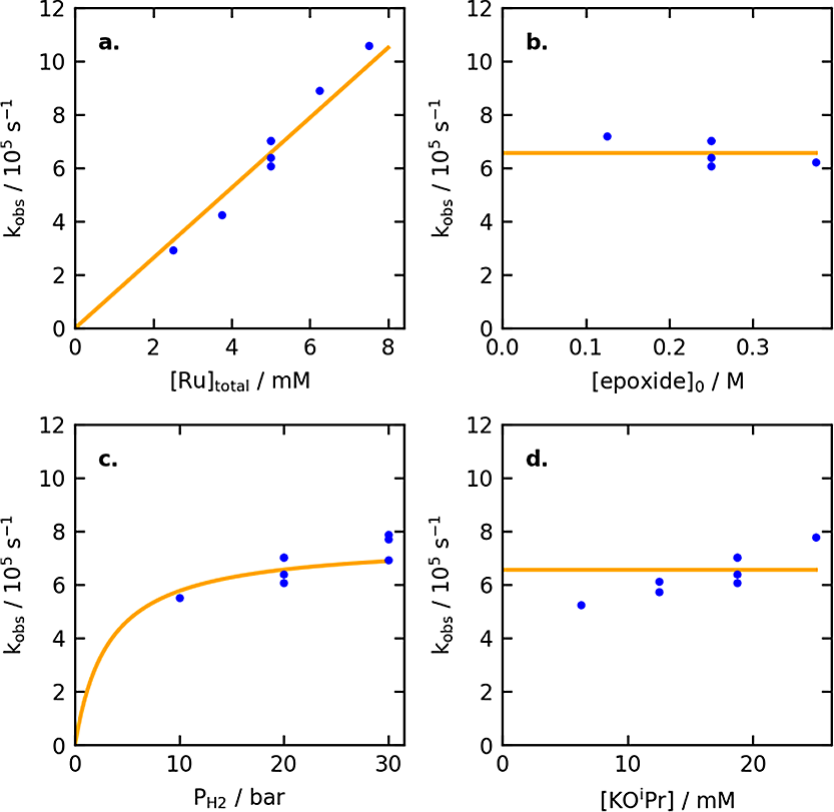
Plots of *k*_obs_ vs [Ru]_total_ (a), [epoxide]_0_ (b), hydrogen pressure (c), and [KO^i^Pr] (d). Blue points represent *k*_obs_ values from independent experiments. Orange lines represent the *k*_obs_ value predicted from a global fit of all 18 experiments.

First, we examined the effect of the concentration of the precatalyst, **RuCl**. As Figure 3a shows, *k*_obs_ increases linearly with [Ru]_total_, consistent with a first-order dependence of the rate on [Ru]_total_. This suggests a monomeric active catalyst species, consistent with the DFT calculations described earlier. Next, we varied the initial concentration of the epoxide, 1-tetradecene oxide. As Figure 3b shows, a minimal effect on *k*_obs_ is observed, consistent with minimal saturation in [epoxide] or product inhibition. We then monitored the reaction under different hydrogen pressures (Figure 3c). The slight increase in *k*_obs_ with increasing hydrogen pressure agrees remarkably well with the above measurements of the equilibrium between the two resting states **RuO**^**i**^**Pr** and **RuH**. Essentially, the increase in *k*_obs_ arises from a higher steady-state mole fraction of **RuH** at higher hydrogen pressures, which increases from 0.76 at 10 bar to 0.90 at 30 bar. Finally, we observed a slight but consistent increase in *k*_obs_ with increasing [KO^i^Pr] (Figure 3d). As described earlier, the alkoxide base is not involved in the turnover-frequency-determining sequence from **RuH** to **e-TS** and is not expected to affect the reaction rate based on the calculated minimum energy pathway. Further investigations into this effect are described in the next section.

Using the above overall rate law and the *K*_1_ value of 89 ± 6 determined from NMR experiments, we calculated *k*_2_ as 0.0152 ± 0.0016 M^–1^·s^–1^ (see the SI for details). Applying the Eyring equation at 298.15 K gives an activation free energy Δ*G*^‡^ of 19.93 ± 0.06 kcal/mol for this step. This experimental barrier, which corresponds to the free energy difference between **RuH-solv** and **e-TS** in Figure 2, is somewhat lower than the barrier of 26.4 kcal/mol calculated by DFT, which may reflect incomplete modeling of the beneficial effect of the alkoxide base in catalysis, as described in more detail below.

### Effect of the Alkali Metal Cation and Added [2.2.2]Cryptand

**Figure 4:**
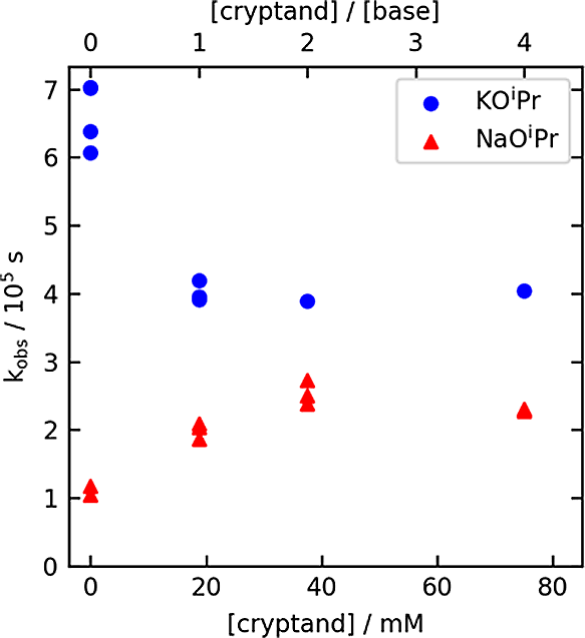
Dependence of *k*_obs_ on the concentration of added [2.2.2]cryptand for epoxide hydrogenolysis with 18.75 mM KO^i^Pr (blue circles) or NaO^i^Pr (red triangles).

Because of the notable effect of the alkali metal cation on product yield during catalyst optimization (K^+^ > Na^+^ > Li^+^, Table 1, entries 22–24), as well as the modest increase in reaction rate with increasing KO^i^Pr concentration (Figure 3d), we decided to examine the rate of epoxide hydrogenolysis with NaO^i^Pr, compared to the optimal base KO^i^Pr. To attempt to deconvolute potential activating vs inhibiting effects of the metal cation, we measured the reaction rate for both bases in the presence of varying amounts of [2.2.2]cryptand, which sequesters both cations strongly in alcohol solvents.^26^Figure 4 shows the dependence of *k*_obs_ on the concentration of added cryptand for both bases.

First, it is notable that, in the absence of added cryptand, the rate of epoxide hydrogenolysis is approximately six times larger for KO^i^Pr vs NaO^i^Pr. This is consistent with the results from optimization (Table 1, entries 23 vs 24). The addition of cryptand slows the reaction with KO^i^Pr and accelerates the reaction with NaO^i^Pr, bringing the *k*_obs_ values closer to one another. Empirically, this points to a reactivity order Na^+^ < [M-cryptand]^+^ < K^+^.

A recent review by Dub summarizes some of the potential causes for the beneficial effect of metal alkoxides in Noyori-type hydrogenation catalysis.^27^ Possible effects of the metal alkoxide include catalyst activation through deprotonation of an acidic site (e.g., replacing N–H with N–K),^28^ substrate activation where a metal alkoxide cluster stabilizes a developing negative charge on substrate oxygen,^29^ and reactivation of catalysts deactivated by trace water.^30^ Further complicating the magnitude of the effect, metal alkoxides are known to aggregate into variably sized clusters in alcohol solvents, which changes the effective concentration of both the alkoxide anion and the metal cation.^27^ Pidko, Filonenko, and co-workers recently described a detailed study of the effects of KO^t^Bu concentration on ester hydrogenation catalyzed by a Noyori-type Mn-pincer catalyst.^31^ In their system, the reaction rate is higher at higher base concentration, and they applied the COSMO-RS solvation model to show that the base concentration affects the free energies of on- and off-cycle Mn species, with the net effect that inhibition by the primary alcohol product is reduced at higher [KO^*t*^Bu]. In our system, the data do not clearly distinguish between these potential effects. We hesitate to draw conclusions from further computation, as even the 6-fold increase in reaction rate for KO^i^Pr vs NaO^i^Pr amounts to only a 1.1 kcal/mol decrease in the overall free energy barrier for catalysis at 25 °C.

### Absence of the NH Group Is Key To Avoid Racemization

**Figure 5:**
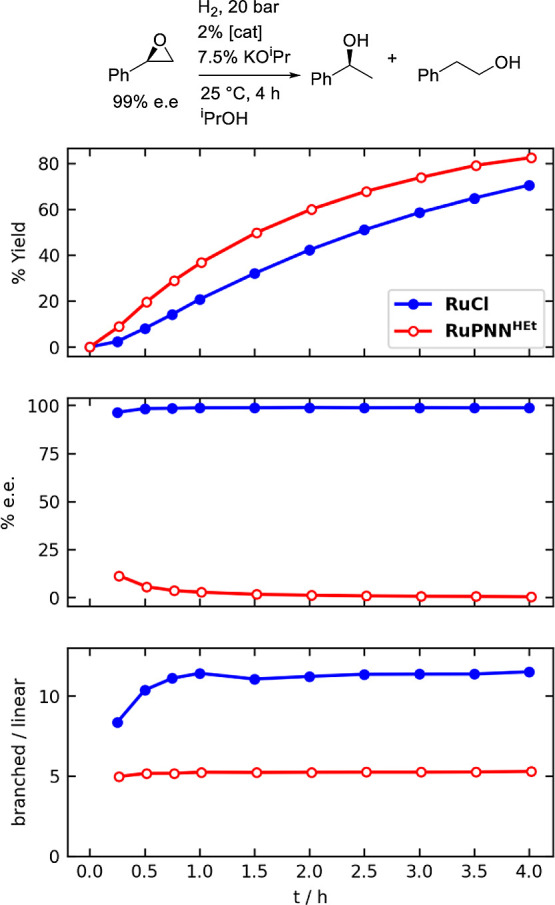
Comparison of (*R*)-styrene oxide hydrogenolysis catalyzed by **RuCl** vs **RuPNN**^**HEt**^. The top panel shows the total yield of hydrogenolysis products, the middle panel shows the e.e. of the 1-phenylethanol product, and the bottom panel shows the branched:linear ratio.

It is noteworthy that **RuCl**, which lacks an NH functional group on the supporting ligand, catalyzes epoxide hydrogenolysis with little to no product racemization, while the closely related **RuPNN**^**HEt**^ gave the racemic product in our previous study.^11^ Because the two catalysts only differ in the nitrogen substituents on the pincer ligand—**RuCl** features an NEt_2_ group while **RuPNN**^**HEt**^ features an NHEt group—we suspected that the NH group was likely involved in product racemization. Because in our prior study, epoxide hydrogenolysis was conducted at 80 °C instead of room temperature and no base was added, we first compared the selectivity of the two catalysts under identical conditions. Figure 5 shows the time course of (*R*)-styrene oxide hydrogenolysis catalyzed by **RuCl** vs **RuPNN**^**HEt**^ at 25 °C with added KO^i^Pr. The two catalysts are similarly active: **RuCl** gives a yield of 65% after 4 h, while **RuPNN**^**HEt**^ gives a yield of 79%. The branched:linear ratio for **RuCl** is nearly constant in the range of 11–12 throughout the reaction; the lower early values likely arise from an imprecise measurement of very low quantities of the linear product at early time points. The branched:linear ratio for **RuPNN**^**HEt**^ is nearly constant in the range of 5.0–5.3 throughout the reaction, in line with previously reported results for this catalyst in isopropyl alcohol solvent at 80 °C with no added base.^11^ As expected, the branched product e.e. for the reaction catalyzed by **RuCl** is maintained at 99% throughout the reaction, indicating no observable racemization over this time period. In contrast, for **RuPNN**^**HEt**^ the product e.e. quickly erodes from an initially measured value of 11% after 15 min, indicating fast product racemization by this catalyst.

**Figure 6:**
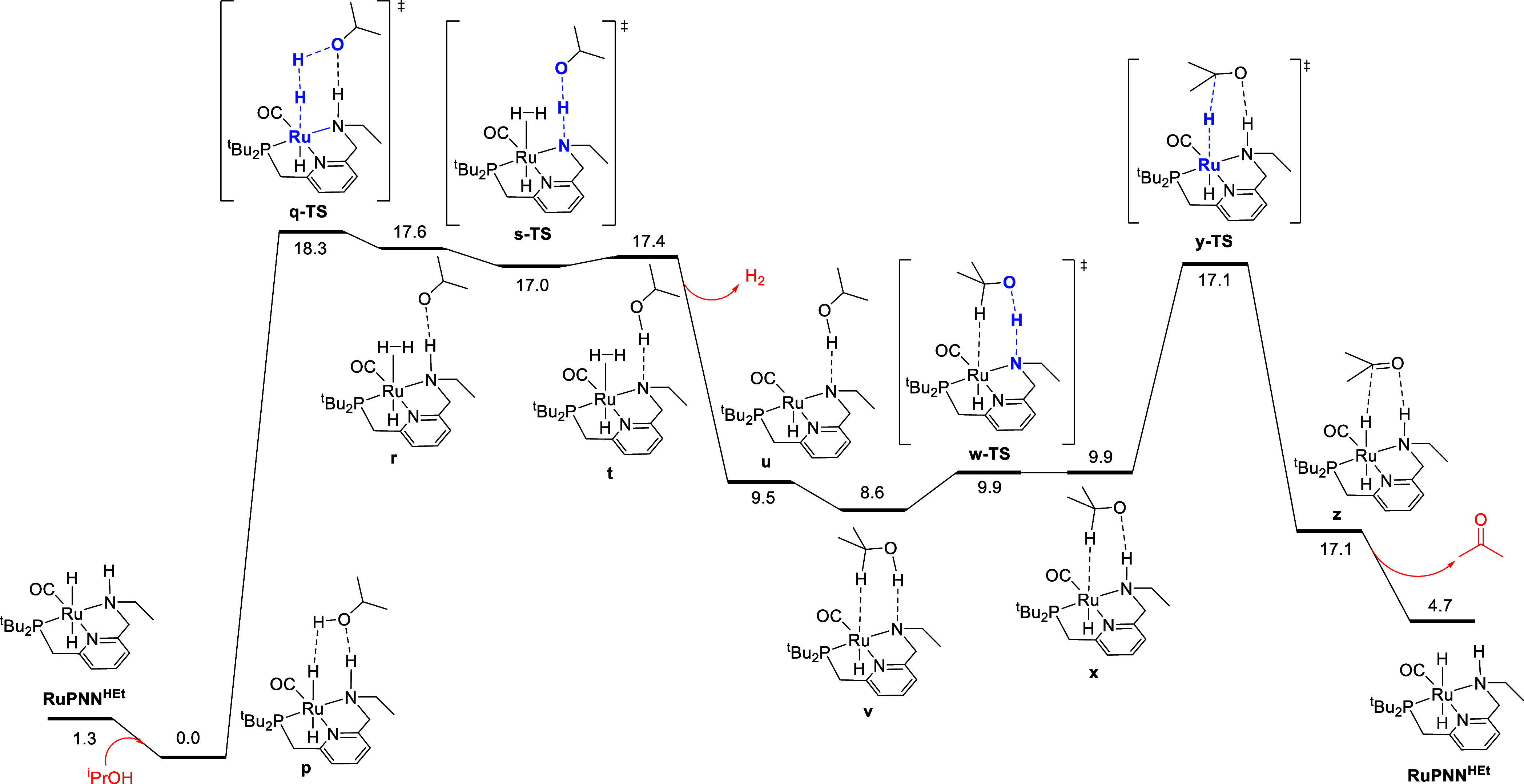
MEP for the reversible dehydrogenation of 2-propanol to acetone, catalyzed by **RuPNN**^**HEt**^. Free energies at 298.15 K are calculated relative to **p**, the calculated catalyst resting state.

In a study on the mechanism of ester hydrogenation, we previously showed that **RuPNN**^**HEt**^ catalyzes the hydrogenation of acetaldehyde to ethanol with a low computed barrier,^20^ with the ligand N–H playing a key role in stabilizing the hydride-transfer transition state. Based on this insight, we hypothesized that product racemization in the current system might occur through reversible Noyori-type dehydrogenation of the secondary alcohol products to the ketones. To examine this hypothesis, we conducted a DFT analysis of the **RuPNN**^**HEt**^-catalyzed dehydrogenation of 2-propanol to acetone. The MEP is shown in Figure 6. The catalyst resting state is **p**, a hydrogen-bonded adduct of **RuPNN**^**HEt**^ and 2-propanol. First, the hydrogen-bonded alcohol protonates the ruthenium hydride through **q-TS**, giving the cationic H_2_ σ-complex **r** as an ion pair with isopropoxide. Then, the isopropoxide ion deprotonates the ligand N–H group through **s-TS** giving the neutral complex **t**. Release of H_2_ then gives **u**, which forms the C–H σ-complex **v**. The pincer nitrogen deprotonates the alcohol oxygen through **w-TS** giving the ion pair **x**, which abstracts hydride from the methine carbon through **y-TS**, giving the acetone complex **z**. Release of acetone completes the thermodynamically unfavorable dehydrogenation, regenerating **RuPNN**^**HEt**^. If a chiral secondary alcohol undergoes such a reversible dehydrogenation, racemization will result. The overall barrier for dehydrogenation is 18.3 kcal/mol, lower than both the computed (26.4 kcal/mol) and experimental (19.9 kcal/mol) barriers for epoxide hydrogenolysis catalyzed by **RuCl**. As stabilization by the N–H group is key in hydride abstraction from the methine carbon through **y-TS**,^32^ an analogous low-barrier mechanism for racemization is not available for **RuCl**.

## Conclusions

In this work, we report the first example of a homogeneous catalyst for the selective formation of highly enantiomerically enriched secondary alcohols via hydrogenolysis of epoxides. The development of the optimized **RuCl**/KO^*t*^Bu catalyst system, which minimizes product racemization, was substantially informed by previous mechanistic work in our group. In particular, the insight that epoxide ring opening mediated by ruthenium-hydrides does not require a Noyori-type Ru–H/N-H unit^14^ spurred us to focus our screening on catalysts lacking an N–H group, as shown in Tables 1 and S1. The knowledge that the PNN- and CNN-pincer-ruthenium complexes shown in Table 1 (entries 1–5), when activated by base, convert to N–H-containing catalysts at elevated temperatures^13^ informed the decision to screen catalysts at room temperature.

For the optimized catalyst system, our proposed mechanism is based on a detailed experimental/computational study. In the MEP calculated by DFT, heterolytic hydrogen activation is mediated by the ruthenium center and exogenous alkoxide base, as previously proposed by Gusev.^22^ Epoxide ring opening is facilitated by an S_N_2-like attack of the ruthenium hydride on the less-hindered epoxide carbon. The calculated MEP led to predictions for hydrogen-pressure-dependent catalyst speciation, validated experimentally by NMR measurements, and an overall rate law for catalysis validated experimentally by kinetics.

## Experimental and Computational Methods

### Catalyst Screening and Optimization

Screening and optimization experiments for catalytic epoxide hydrogenolysis were conducted in a 450 mL Parr 4760 reaction vessel with a stainless-steel insert designed to hold eight 13 mm disposable glass test tubes. First, the Parr reactor was brought into the glovebox. Each reaction mixture was prepared in the glovebox by transferring the appropriate metal catalyst, additive, solvent (2 mL total reaction volume), the appropriate amount of (*R*)-styrene oxide (98% ee, Sigma-Aldrich, 0.25 or 1.0 mmol, according to Table S1), and 0.20 equiv. *n*-tetradecane as an internal standard to an oven-dried test tube, along with a stir bar. The reaction tubes (up to eight, all with the same solvent) were transferred to the Parr reactor, which was sealed and removed from the glovebox. The reactor was then pressurized to 30 bar with hydrogen and stirred magnetically for 18 h at the appropriate temperature (25 or 80 °C). After cooling to room temperature if necessary, the reactor was carefully vented and opened. A 30 μL aliquot of each reaction mixture was removed and diluted into a GC vial with 1 mL toluene, and then analyzed by GC-FID. The reactants and products were separated using a 30 m Agilent Cyclosil-B column, first holding at 100 °C for 1 min, then ramping to 180 °C at a rate of 5 °C/min, and then holding at 180 °C for 5 min. Styrene oxide elutes at 7.00 min (*R*) and 7.14 min (*S*), 1-phenylethanol elutes at 9.72 min (*R*) and 9.92 min (*S*), 2-phenylethanol elutes at 10.42 min, and tetradecane elutes at 11.89 min. Conversion, yield, and e.e. were determined by comparing the appropriate integrations against the tetradecane standard. The results from selected experiments are shown in Table 1, and the complete list of 104 experiments is shown in Table S1.

### Epoxide Hydrogenolysis End Point Analysis Experiments Shown in Table 2

In the glovebox, the epoxide (1.0 mmol), **RuCl** (1.0 mol %), KO^*t*^Bu (2.5 mol %), and *n*-tetradecane (0.20 equiv) as an internal standard were added to isopropyl alcohol (2.0 mL total volume) and transferred to an oven-dried test tube with a stir bar. The test tube was placed in a stainless-steel pressure reactor, which was sealed and brought out of the glovebox. The reactor was pressurized with 30 bar H_2_. The reactor was then kept at room temperature (25 °C) using a water bath and was stirred magnetically for 18 h. The reactor was then carefully vented and opened to air. A 30 μL aliquot of the reaction mixture was removed, diluted in 1 mL toluene, and analyzed by GC-FID. Conversion and yields were calculated based on the calibrated response factors of the reactant and products relative to the tetradecane internal standard. Reactions were analyzed by GC-FID on either a 30 m Agilent Cyclosil-B column or a 30 m Supelco Alpha DEX 120 column. The GC temperature programs and relevant retention times are listed in Table S2 in the Supporting Information.

### Gram-Scale Hydrogenolysis of (*S*)-Glycidyl Phenyl Ether

A 100 mL stainless-steel reactor and an oven-dried glass liner were brought into the glovebox. A stirbar, glycidyl phenyl ether (1.00 g, 6.7 mmol), KO^*t*^Bu (18.7 mg, 0.17 mmol), **RuCl** (32.5 mg, 0.067 mmol), and isopropyl alcohol (13.3 mL) were added to the glass liner. The glass liner was placed in the reactor, which was sealed and removed from the glovebox. The reactor was pressurized with 30 bar H_2_. The reactor was kept at 25 °C in a water bath and was stirred magnetically for 18 h. The reactor was then carefully vented and opened to air. The reaction solution was transferred to a round-bottom flask and volatiles were removed by rotary evaporation. The crude material was then purified by silica gel chromatography using a gradient of 0–40% ethyl acetate in hexanes and transferred to a round-bottom flask, then the solvent was removed by rotary evaporation. Yield of (*S*)-1-phenoxypropan-2-ol: 0.896 g, 88%, >99% e.e.

### Gram-Scale Hydrogenolysis of (*S*)-Benzyl Glycidyl Ether

A 100 mL stainless-steel reactor and an oven-dried glass liner were brought into the glovebox. A stirbar, benzyl glycidyl ether (1.00 g, 6.09 mmol), KO^*t*^Bu (18.7 mg, 0.17 mmol), **RuCl** (32.5 mg, 0.067 mmol), and isopropyl alcohol (13.3 mL) were added to the glass liner. The glass liner was placed in the reactor, which was sealed and removed from the glovebox. The reactor was pressurized with 30 bar H_2_. The reactor was kept at 25 °C in a water bath and was stirred magnetically for 18 h. The reactor was then carefully vented and opened to air. The reaction solution was transferred to a round-bottom flask, and volatiles were removed by rotary evaporation. The crude material was then purified by silica gel chromatography using a gradient of 0 to 50% ethyl acetate in hexanes and transferred to a round-bottom flask, then the solvent was removed by rotary evaporation. Yield of (*S*)-1-benzyloxypropan-2-ol: 0.922 g, 91%, >99% e.e.

### Computational Methods

DFT calculations were performed using the Gaussian 16 computational chemistry package, Revision B.01.^33^ The geometries and energies of all species were calculated using the dispersion-corrected hybrid functional ωB97X-D^34^ and the def2-SVP basis set.^35^ A superfine integration grid was used for all calculations, which aided convergence of structures with loosely bound fragments such as explicit isopropyl alcohol molecules. Complete structures with no truncations were used in all cases. Geometry optimization and frequency calculation were conducted in a solvent, using a polarizable continuum with radii and nonelectrostatic terms from Truhlar and co-workers’ SMD solvation model, and with dielectric constants chosen for 2-propanol.^36^ Geometry optimization in the solvent is important to identify ion-pair intermediates that might be missed in the gas phase.^37^

Frequency calculations ensured the absence of imaginary vibrational modes in intermediates and the presence of exactly one imaginary mode in transition states. Intrinsic reaction coordinate calculations were employed to verify that transition states led to the specified minima. Free energy corrections were calculated at the experimental reaction temperature of 25 °C, or 298.15 K. Standard-state corrections were applied in order to adjust from 1 atm to 1 M for solution-phase free energies, amounting to 1.89 kcal/mol added to the free energy of each isolated molecule at 298.15 K.^38^ For the solvent 2-propanol, the standard state is its neat molarity of 13.08 M, corresponding to a correction of 3.42 kcal/mol from 1 atm. Although the standard state for molecular hydrogen is sometimes taken as the gas at 1 atm, we have used a 1 M standard state in 2-propanol, for consistency in computing reaction kinetics from the calculated free energies. The solvation-corrected electronic energies were further refined using the same ωB97X-D functional and the larger basis set def2-TZVP.^35^ All energies reported in this article are standard-state free energies at 298.15 K. Tables of calculated energies are provided in the Supporting Information, and geometries in Cartesian coordinates are included in a separate, compiled.XYZ file.

### Apparatus for Kinetic Experiments

Kinetic experiments were conducted in an Asynt Multicell Parallel High-Pressure Reactor, designed to allow sampling of aliquots from five hydrogenation reactions run in parallel. Our customization of this apparatus was described previously^13^ but is repeated here for convenience. The reactor was customized to fit in our glovebox antechamber and to include reaction sampling valves on five reactor cells. The sampling valves employ 0.8 mm ID 1/16″ stainless-steel tubing, a Swagelok Low Flow metering valve (part no. SS-SS1) to control flow, and a Swagelok ball valve (part no. SS-41GS1) to allow removal of samples. As the internal volume of the sampling valve system was measured to be 0.25 mL, removal of 0.50 mL of liquid before collecting each aliquot ensures that a fresh sample is being taken directly from the reaction mixture. A sixth reactor cell is fitted with a thermocouple to allow control of the internal reaction temperatures. For reactions at 25 °C, the reactor was partially submerged (2 cm past the internal liquid level) in a stirred water bath (a nonmagnetic stainless-steel cooking pot), whose temperature is controlled using a stirring hot plate with a temperature probe. The stirring hot plate allows stirring of the reactions and water bath.

### Experimental Procedure for Kinetic Experiments

First, the water bath was preheated to 25 °C. The Asynt reactor was brought into the glovebox with oven-dried glass reactor liners and Teflon-coated stir bars. Reaction solutions, with a total volume of 10.0 mL, were prepared with the appropriate amount of ruthenium complex, racemic 1-tetradecene oxide, KO^i^Pr solution (5% in isopropyl alcohol), optionally [2.2.2]cryptand, and tetradecane (0.20 equiv relative to epoxide) as internal standard. In selected experiments, KO^i^Pr was replaced by NaO^i^Pr. The reactor was closed and removed from the glovebox and allowed to incubate for 20 min in the water bath. After gently purging the H_2_ line for 2 min, the reactor was pressurized to the desired pressure. Aliquots were removed at predetermined times for analysis by gas chromatography. To ensure that samples represented the reaction mixture without contamination from the transfer line, 0.5 mL of the reaction mixture was discarded before one drop was collected for each aliquot. Each one-drop aliquot was diluted with 1 mL toluene in a crimp-top vial, then sealed and analyzed by GC-FID. The concentrations of reactants and products at each time point were determined by integration of their GC signals against the tetradecane standard. GC analysis employed a 30 m Agilent Cyclosil-B column. The oven temperature was initially held at 100 °C for 1 min, then increased to 220 °C at a rate of 5 °C/min, and then held at 220 °C for 5 min. With these parameters, tetradecene oxide was eluted at 18.14 min, 2-tetradecanol was eluted at 18.71 min, and the standard tetradecane was eluted at 11.77 min. Although the chiral column was used to minimize the need to switch columns repeatedly for different experiment types, the reactant and product enantiomers were not separated by this temperature program. The concentrations measured over time in these experiments are reported in Tables S5–S7.

## Supporting Information Available

Experimental and computational details (PDF)
Atomic coordinates for all computed molecular structures, compiled as one file readable by the free program Mercury^39^ (XYZ)
Animations showing the imaginary vibrational mode for all calculated transition states (ZIP)

## Supplementary Material

om4c00214_si_001.pdf

om4c00214_si_002.xyz
