# Supplementary material for: Highly Enantiomerically Enriched Secondary Alcohols via Epoxide Hydrogenolysis
Source: Organometallics. 2024 Jun 17;43(13):1490–501. doi: 10.1021/acs.organomet.4c00214 (PMC11234370; doi:10.1021/acs.organomet.4c00214)
Supplement: Supplementary file 1 — om4c00214_si_001.pdf [file om4c00214_si_001.pdf]

Supporting Information for

Highly Enantiomerically Enriched Secondary Alcohols via Epoxide Hydrogenolysis

Olivia J. Borden, Benjamin T. Joseph, Marianna C. Head, Obsidian A. Ammons, Diane Eun Kim, Abigail C. Bonino, Jason M. Keith*, and Anthony R. Chianese*

Department of Chemistry, Colgate University, 13 Oak Drive, Hamilton, New York 13346, United States

Emails: J. M. Keith, jkeith@colgate.edu; A. R. Chianese, achianese@colgate.edu

Table of Contents

General Methods. 2

Complete List of Results from 104 Screening and Optimization Experiments. 2

Chiral GC Traces for Racemic and Enantiomerically Enriched Products 7

NMR Spectra for Isolated Products of Epoxide Hydrogenolysis 17

Energies Calculated by DFT 19

Alternative Pathway: Ring Opening Including Explicit 2-Propanol 21

Alternative Pathway: Hydrogen Activation Without Explicit 2-Propanol 21

Alternative Pathway: Hydrogen Activation Through the NCH_2 Linker 22

Alternative Pathway: Hydrogen Activation Through the PCH_2 Linker 22

Derivation of the Rate Law for Catalytic Epoxide Hydrogenolysis 23

Determination of the Equilibrium Constant K_1 by NMR Spectroscopy 24

Concentrations Measured in Kinetic Experiments 26

Determination of the Rate Constant k_2 from Kinetic Data 30

Comparison of (R)-Styrene Oxide Hydrogenolysis by RuCl and $RuPNN^{HEt}$ 32

References 33

General Methods. All epoxide hydrogenolysis reactions were assembled in an argon-filled MBraun Labmaster 130 glovebox. Solvents were purchased in anhydrous form from EMD-Millipore or Acros and were deoxygenated by sparging with argon before bringing into the glovebox. Gas chromatography was conducted using a Shimadzu 2030 system equipped with an FID detector. Hydrogen gas was purchased from Airgas at the Ultrahigh Purity level. **RuCl**, **Ru-MACHO**, **Ru-MACHO-BH**, and **Ru-MACHO-tBu**, were purchased from Strem Chemicals. **Mn-PNP-iPr** was purchased from TCI America. $RuHClCO(PPh_3)_3$ purchased from Alfa Aesar. **RuCNN-dipp-Me**,[1] **RuCNN-dipp-Et**,[1] **RuCNN-dipp-iPr**,[1] **RuCNN-Mes-Me**,[2] **RuCNN-Mes-Me**,[2] **RuCNN-Et-imine**,[3] **IrCCCMes**,[4] and **IrCCCAd**[5] were synthesized as described previously. (*R*)-Styrene oxide was purchased from Sigma-Aldrich, (*S*)-benzyl glycidyl ether was purchased from TCI America, and (*S*)-glycidol was purchased from Thermo Scientific. All other epoxides were purchased in racemic form and resolved as described by Jacobsen and coworkers.[6]

Complete List of Results from 104 Screening and Optimization Experiments. Table 1 in the main text shows results from selected screening and optimization experiments. The results of all such experiments are shown below in Table S1. Chart S1 shows the structures of all catalysts tested.

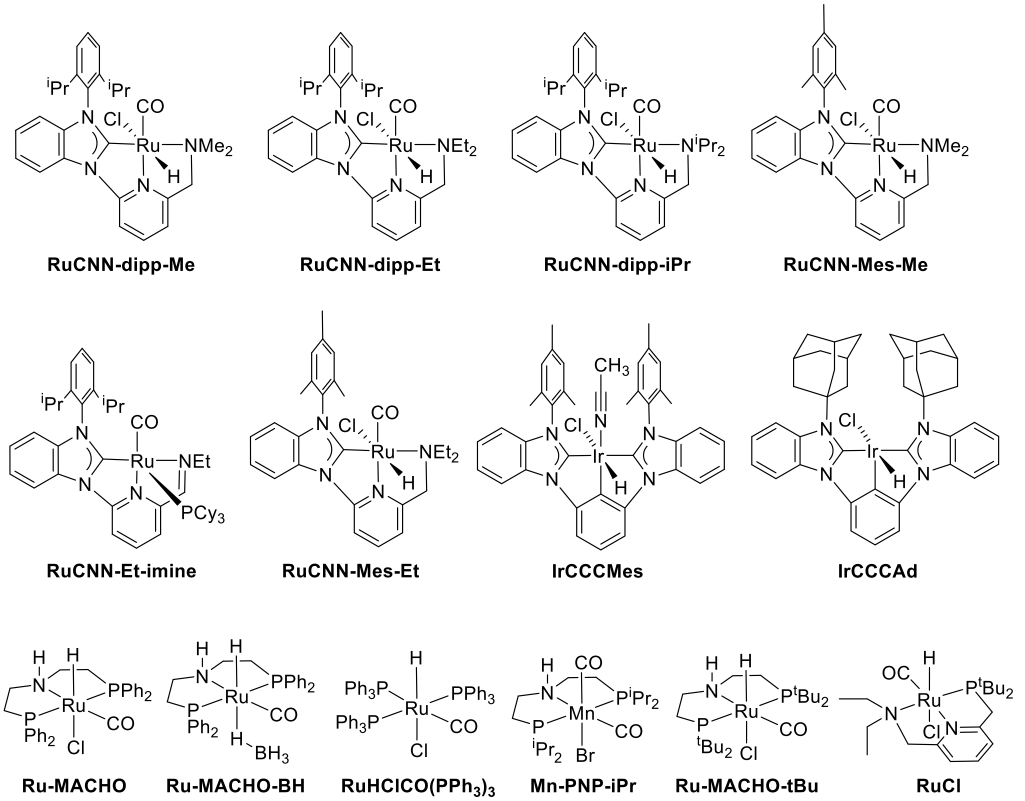

Chart S1. Precatalysts used in screening, as listed below in Table S1.

Table S1. Results of catalyst screening and optimization. Entries in bold are also included in Table 1 in the main text. The column "e.e. (%)" lists the enantiomeric excess of the 1-phenylethanol product, and "b:l" lists the ratio of the branched 1-phenylethanol product to the linear 2-phenylethanol product.

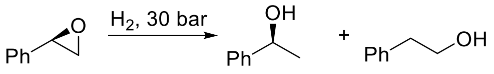

Catalyst	mol %	Additive	mol %	Solvent	T (°C)	[epoxide] (M)	Yield (%)	e.e. (%)	b:l
RuCNN-dipp-Me	1	NaO^tBu	10	toluene	80	0.125	>99	6	3.4
RuCNN-dipp-Et	1	NaO^tBu	10	toluene	80	0.125	42	0	2.7
RuCNN-dipp-iPr	1	NaO^tBu	10	toluene	80	0.125	31	0	8.2
RuCNN-Mes-Me	1	NaO^tBu	10	toluene	80	0.125	100	5	1.8
RuCNN-Mes-Me	1	none	--	toluene	80	0.125	0	--	--
IrCCC-Mes	1	NaO^tBu	10	toluene	80	0.125	6	14	5.2
IrCCC-Ad	1	NaO^tBu	10	toluene	80	0.125	5	13	3
RuCNN-Et-imine	1	none	--	^iPrOH	80	0.125	>99	0	6.0
RuCNN-Et-imine	1	NaO^tBu	10	^iPrOH	80	0.125	>99	0	6.0
RuCNN-dipp-Me	1	NaO^tBu	10	^iPrOH	80	0.125	98	0	6.2
RuCNN-dipp-Me	1	none	--	^iPrOH	80	0.125	1	2	2.9
RuCNN-dipp-iPr	1	NaO^tBu	10	^iPrOH	80	0.125	>99	0	7.5
Ru-MACHO	1	NaO^tBu	10	^iPrOH	80	0.125	>99	0	11.5
Ru-MACHO-BH	1	none	--	^iPrOH	80	0.125	>99	0	11.1
$RuHClCO(PPh_3)_3$	1	NaO^tBu	10	^iPrOH	80	0.125	4	94	4.3
Mn-PNP-iPr	1	NaO^tBu	10	^iPrOH	80	0.125	0	--	--
Mn-PNP-iPr	1	none	--	^iPrOH	80	0.125	5	95	28.7
Ru-MACHO-tBu	1	NaO^tBu	10	^iPrOH	80	0.125	0	--	--
Ru-MACHO-tBu	1	none	--	^iPrOH	80	0.125	9	88	32.7
RuCNN-Et-imine	1	none	--	MeOH	80	0.125	10	36	3.1
RuCNN-Et-imine	1	NaO^tBu	10	MeOH	80	0.125	8	5	3.4
RuCNN-dipp-Me	1	NaO^tBu	10	MeOH	80	0.125	10	89	3
RuCNN-dipp-Me	1	none	--	MeOH	80	0.125	13	98	0.15
RuCNN-dipp-iPr	1	NaO^tBu	10	MeOH	80	0.125	20	92	5.3
Ru-MACHO	1	NaO^tBu	10	MeOH	80	0.125	98	4	7.2
Ru-MACHO-BH	1	none	--	MeOH	80	0.125	99	58	6.8
RuHClCO(PPh3)3	1	NaO^tBu	10	MeOH	80	0.125	7	96	5.1
RuCNN-dipp-Me	1	NaO^tBu	10	toluene	25	0.125	5	61	7.9
RuCNN-dipp-Et	1	NaO^tBu	10	toluene	25	0.125	8	42	5.7
RuCNN-dipp-iPr	1	NaO^tBu	10	toluene	25	0.125	6	16	10
RuCNN-Mes-Me	1	NaO^tBu	10	toluene	25	0.125	14	62	2.6
RuCNN-Mes-Et	1	NaO^tBu	10	toluene	25	0.125	8	46	4.8
IrCCC-Mes	1	NaO^tBu	10	toluene	25	0.125	1	60	>10
**RuCl**	**1**	**NaO^tBu**	**10**	**toluene**	**25**	**0.125**	**2**	**98**	**>10**
RuCNN-dipp-Me	1	NaO^tBu	10	^tAmOH	25	0.125	21	36	11.7
RuCNN-dipp-Et	1	NaO^tBu	10	^tAmOH	25	0.125	41	32	12.3
RuCNN-dipp-iPr	1	NaO^tBu	10	^tAmOH	25	0.125	17	57	9.6

RuCNN-Mes-Me	1	NaO^{t}Bu	10	^{t}AmOH	25	0.125	6	49	7.6
RuCNN-Mes-Et	1	NaO^{t}Bu	10	^{t}AmOH	25	0.125	7	36	7.5
IrCCC-Mes	1	NaO^{t}Bu	10	^{t}AmOH	25	0.125	1	98	>10
IrCCC-Ad	1	NaO^{t}Bu	10	^{t}AmOH	25	0.125	5	13	3
**RuCl**	**1**	**NaO^{t}Bu**	**10**	**^{t}AmOH**	**25**	**0.125**	**62**	**93**	**19.1**
RuCNN-Et-imine	1	none	--	^{i}PrOH	25	0.125	0	--	--
RuCNN-Et-imine	1	NaO^{t}Bu	10	^{i}PrOH	25	0.125	3	98	>10
**RuCNN-dipp-Me**	**1**	**NaO^{t}Bu**	**10**	**^{i}PrOH**	**25**	**0.125**	**74**	**92**	**7.4**
**RuCNN-dipp-Et**	**1**	**NaO^{t}Bu**	**10**	**^{i}PrOH**	**25**	**0.125**	**>99**	**79**	**7.7**
RuCNN-dipp-iPr	1	NaOtBu	10	^{i}PrOH	25	0.125	9	92	7.3
**RuCNN-Mes-Me**	**1**	**NaO^{t}Bu**	**10**	**^{i}PrOH**	**25**	**0.125**	**79**	**91**	**5.5**
**RuCNN-Mes-Et**	**1**	**none**	**--**	**^{i}PrOH**	**25**	**0.125**	**62**	**92**	**5.2**
IrCCC-Mes	1	NaO^{t}Bu	10	^{i}PrOH	25	0.125	2	98	6.4
**RuCl**	**1**	**NaO^{t}Bu**	**10**	**^{i}PrOH**	**25**	**0.125**	**31**	**98**	**11.1**
RuCNN-dipp-Me	1	NaO^{t}Bu	10	EtOH	25	0.125	11	97	5.6
RuCNN-dipp-Et	1	NaO^{t}Bu	10	EtOH	25	0.125	11	97	5
RuCNN-dipp-iPr	1	NaO^{t}Bu	10	EtOH	25	0.125	3	98	9.8
RuCNN-Mes-Me	1	NaO^{t}Bu	10	EtOH	25	0.125	6	98	5
RuCNN-Mes-Et	1	none	--	EtOH	25	0.125	4	98	4.05
IrCCC-Mes	1	NaO^{t}Bu	10	EtOH	25	0.125	22	90	8.2
IrCCC-Ad	1	NaO^{t}Bu	10	EtOH	25	0.125	2	92	1.1
RuCl	1	NaO^{t}Bu	10	EtOH	25	0.125	3	98	6.7
**RuCl**	**1**	**none**	**--**	**^{i}PrOH**	**25**	**0.125**	**0**	**--**	**--**
**RuCl**	**1**	**CsF**	**10**	**^{i}PrOH**	**25**	**0.125**	**0**	**--**	**--**
**RuCl**	**1**	**Cs_2CO_3**	**10**	**^{i}PrOH**	**25**	**0.125**	**24**	**98**	**11.5**
**RuCl**	**1**	**KF**	**10**	**^{i}PrOH**	**25**	**0.125**	**3**	**--**	**0**
**RuCl**	**1**	**BEMP**	**10**	**^{i}PrOH**	**25**	**0.125**	**4**	**96**	**9.5**
**RuCl**	**1**	**$LiAlH_4$**	**10**	**^{i}PrOH**	**25**	**0.125**	**3**	**98**	**11.6**
**RuCl**	**1**	**$NaBH_4$**	**10**	**^{i}PrOH**	**25**	**0.125**	**5**	**93**	**5.1**
**RuCl**	**1**	**KOAc**	**10**	**^{i}PrOH**	**25**	**0.125**	**0**	**--**	**--**
**RuCl**	**1**	**K_3PO_4**	**10**	**^{i}PrOH**	**25**	**0.125**	**8**	**98**	**9.5**
**RuCl**	**1**	**KO^{t}Bu**	**10**	**^{i}PrOH**	**25**	**0.125**	**57**	**98**	**11.3**
RuCl	1	KO^{t}Bu	10	^{i}PrOH	25	0.125	44	98	11.3
RuCl	0.25	KO^{t}Bu	2.5	^{i}PrOH	25	0.5	41	98	11.9
RuCl	1	KO^{t}Bu	40	^{i}PrOH	25	0.125	54	98	11.6
RuCl	4	KO^{t}Bu	10	^{i}PrOH	25	0.125	>99	98	11.5
**RuCl**	**0.25**	**KO^{t}Bu**	**10**	**^{i}PrOH**	**25**	**0.5**	**36**	**98**	**12.2**
RuCl	4	KO^{t}Bu	40	^{i}PrOH	25	0.125	>99	98	11.8
**RuCl**	**1**	**KO^{t}Bu**	**2.5**	**^{i}PrOH**	**25**	**0.5**	**>99**	**98**	**11.9**
**RuCl**	**1**	**KO^{t}Bu**	**10**	**^{i}PrOH**	**25**	**0.5**	**>99**	**98**	**12**
RuCl	1	LiO^{i}Pr	10	^{i}PrOH	25	0.125	37	98	11.2
RuCl	0.25	LiO^{i}Pr	2.5	^{i}PrOH	25	0.5	30	98	11.9
RuCl	1	LiO^{i}Pr	40	^{i}PrOH	25	0.125	35	98	11.5
RuCl	4	LiO^{i}Pr	10	^{i}PrOH	25	0.125	61	98	11.4
RuCl	0.25	LiO^{i}Pr	10	^{i}PrOH	25	0.5	28	98	12.1

RuCl	4	LiO^{i}Pr	40	^{i}PrOH	25	0.125	61	98	11.6
**RuCl**	**1**	**LiO^{i}Pr**	**2.5**	**^{i}PrOH**	**25**	**0.5**	**52**	**98**	**11.9**
RuCl	1	LiO^{i}Pr	10	^{i}PrOH	25	0.5	51	98	12.1
RuCl	1	NaO^{i}Pr	10	^{i}PrOH	25	0.125	41	98	11.5
RuCl	0.25	NaO^{i}Pr	2.5	^{i}PrOH	25	0.5	36	98	11.9
RuCl	1	NaO^{i}Pr	40	^{i}PrOH	25	0.125	58	98	11.4
RuCl	4	NaO^{i}Pr	10	^{i}PrOH	25	0.125	68	98	11.4
RuCl	0.25	NaO^{i}Pr	10	^{i}PrOH	25	0.5	52	98	12.1
RuCl	4	NaO^{i}Pr	40	^{i}PrOH	25	0.125	95	98	11.5
**RuCl**	**1**	**NaO^{i}Pr**	**2.5**	**^{i}PrOH**	**25**	**0.5**	**60**	**98**	**12.0**
RuCl	1	NaO^{i}Pr	10	^{i}PrOH	25	0.5	84	98	12.0
RuCl	1	KO^{i}Pr	10	^{i}PrOH	25	0.125	80	98	11.5
RuCl	0.25	KO^{i}Pr	2.5	^{i}PrOH	25	0.5	76	98	12.0
RuCl	1	KO^{i}Pr	40	^{i}PrOH	25	0.125	83	98	11.6
RuCl	4	KO^{i}Pr	10	^{i}PrOH	25	0.125	>99	98	11.6
RuCl	0.25	KO^{i}Pr	10	^{i}PrOH	25	0.5	80	98	12.1
RuCl	4	KO^{i}Pr	40	^{i}PrOH	25	0.125	99	98	11.6
**RuCl**	**1**	**KO^{i}Pr**	**2.5**	**^{i}PrOH**	**25**	**0.5**	**>99**	**98**	**11.9**
RuCl	1	KO^{i}Pr	10	^{i}PrOH	25	0.5	>99	98	12.0

Table S2. GC temperature programs and retention times for chiral epoxides and their hydrogenolysis products.

			Retention times (min)[a]					
			Epoxide		Branched		Linear	Tetradecane
**Substrate**	**Column**	**Temperature Program**	**(R)**	**(S)**	**(R)**	**(S)**		
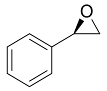	Cyclosil-B	hold at 100 °C for 1 min, ramp to 180 °C at 5 °C/min, hold at 180 °C for 1 min	7.00	7.14	9.72	9.92	10.42	11.91
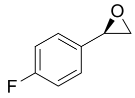	Cyclosil-B	hold at 90 °C for 90 min, ramp to 220 °C at 5 °C/min, hold at 220 °C for 5 min	18.33	19.60	55.50	61.33	68.48	80.77
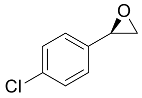	Cyclosil-B	hold at 100 °C for 120 min, ramp to 220 °C at 5 °C/min, hold at 220 °C for 10 min	40.10	42.94	112.32	123.83	127.57	47.95
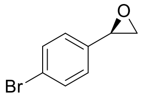	Cyclosil-B	hold at 120 °C for 90 min, ramp to 180 °C at 20 °C/min, hold at 180 °C for 10 min	29.10	30.52	66.15	69.81	85.01	17.42
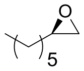	Alpha DEX 120	hold at 40 °C for 85 min, ramp to 60 °C at 1 °C/min, hold at 60 °C for 45 min, ramp to 220 °C at 10 °C/min, hold at 220 °C for 10 min	84.60	83.17	113.07	114.22	--	159.62
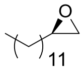	Alpha DEX 120	hold at 110 °C for 130 min, ramp to 220 °C at 20 °C/min, hold at 220 °C for 9.5 min	99.42[b]	99.42[b]	119.14	117.78	--	17.19
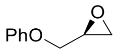	Cyclosil-B	hold at 110 °C for 40 min, ramp to 180 °C at 20 °C/min, hold at 180 °C for 4 min	34.25	33.83	37.02	35.72	--	28.83
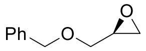	Cyclosil-B	hold at 110 °C for 90 min, ramp to 180 °C at 20 °C/min, hold at 180 °C for 10 min	48.45	46.57	43.47	42.39	--	28.83
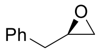	Cyclosil-B	hold at 100 °C for 60 min, ramp to 220 °C at 20 °C/min, hold at 220 °C for 10 min	22.04	22.53	30.20	28.50	--	46.93
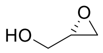	Cyclosil-B	hold at 50 °C for 90 min, ramp to 220 °C at 20 °C/min, hold at 220 °C for 10 min	13.39	14.25	33.47	32.17	--	97.17

[a] The underlined retention times indicate the major enantiomer of the epoxide reactant and branched product listed in Table 2 in the main text. [b] The enantiomers of 1-tetradecene oxide were not resolved with either available GC column. Because the 2-tetradecanol product after hydrogenolysis was measured to have >99% e.e., we infer that the epoxide reactant also had >99% e.e.

Chiral GC Traces for Racemic and Enantiomerically Enriched Products

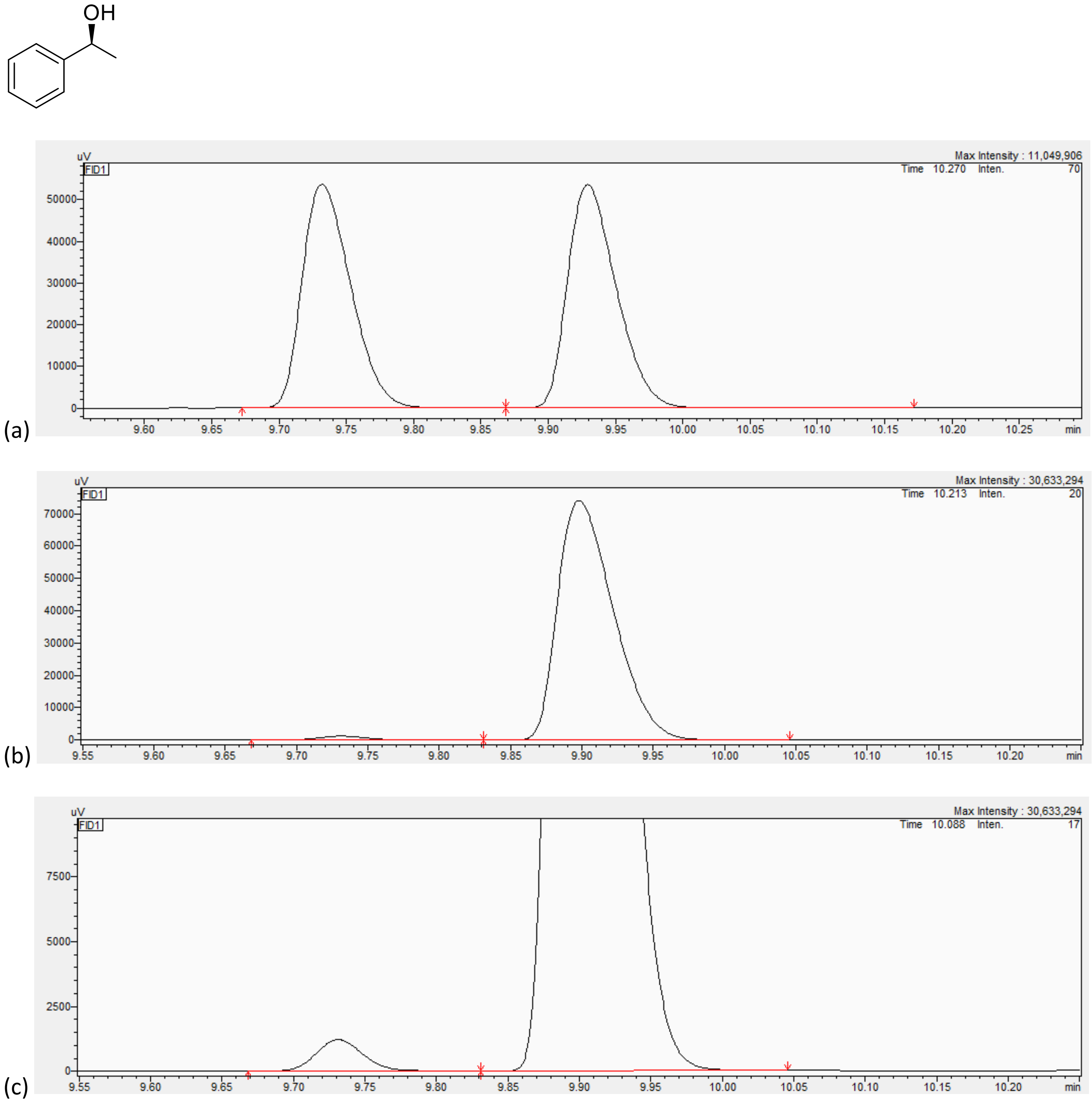

Figure S1. Chiral GC traces for 1-phenyl-ethanol: (a) racemic; (b) 98% e.e; (c) 98% e.e. zoomed in.

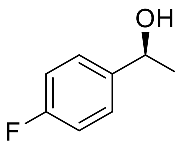

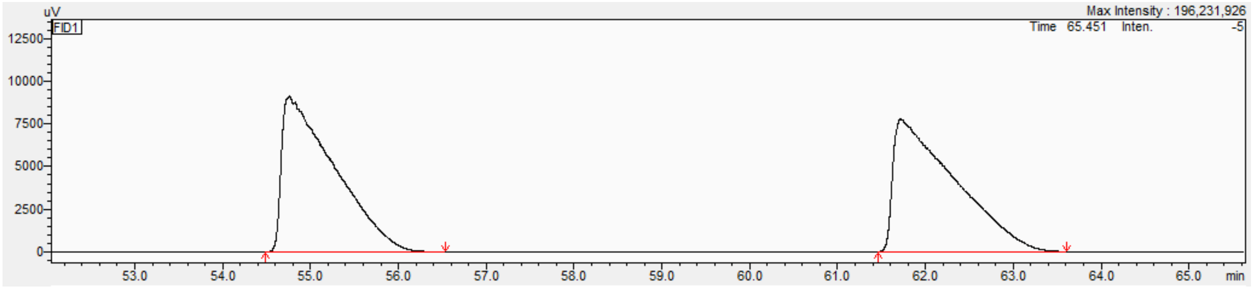

(a)

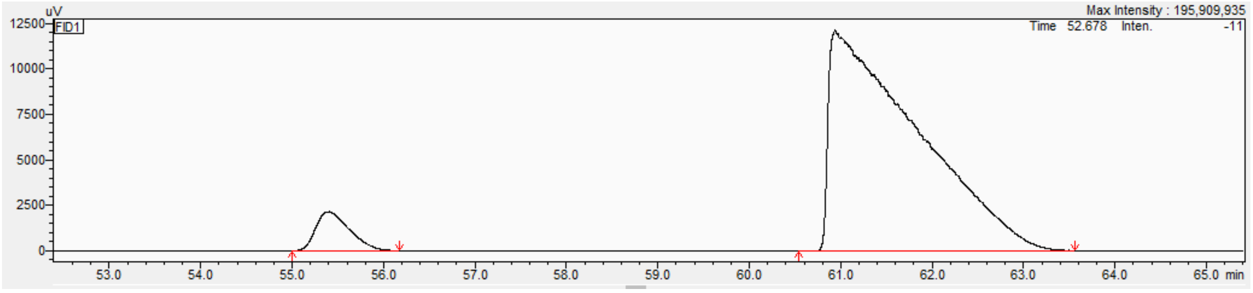

(b)

Figure S2. Chiral GC traces for 1-(4-fluorophenyl)-ethanol: (a) racemic; (b) 85% e.e.

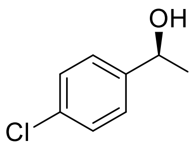

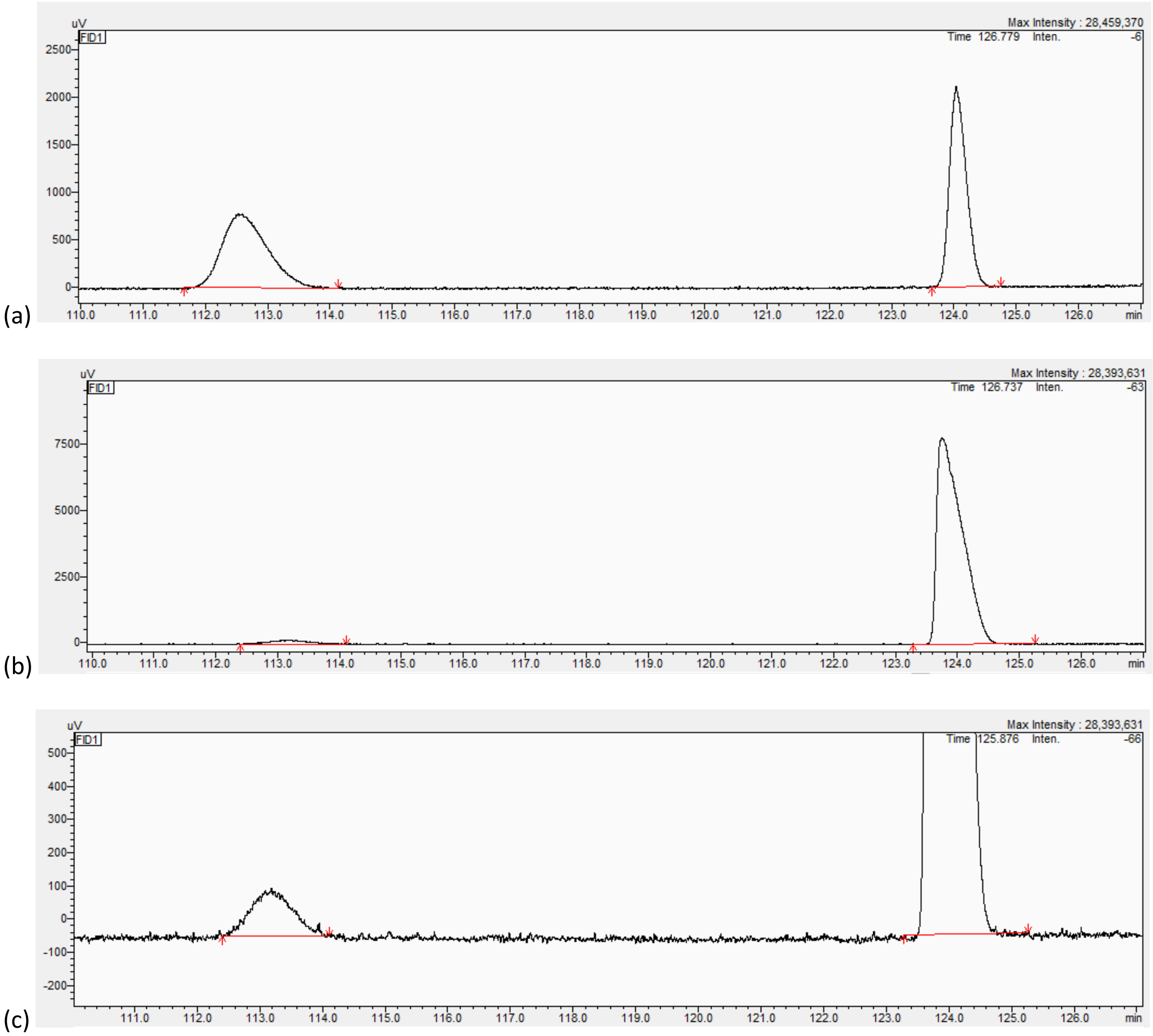

Figure S3. Chiral GC traces for 1-(4-chlorophenyl)-ethanol: (a) racemic; (b) 94% e.e; (c) 94% e.e. zoomed in.

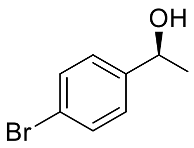

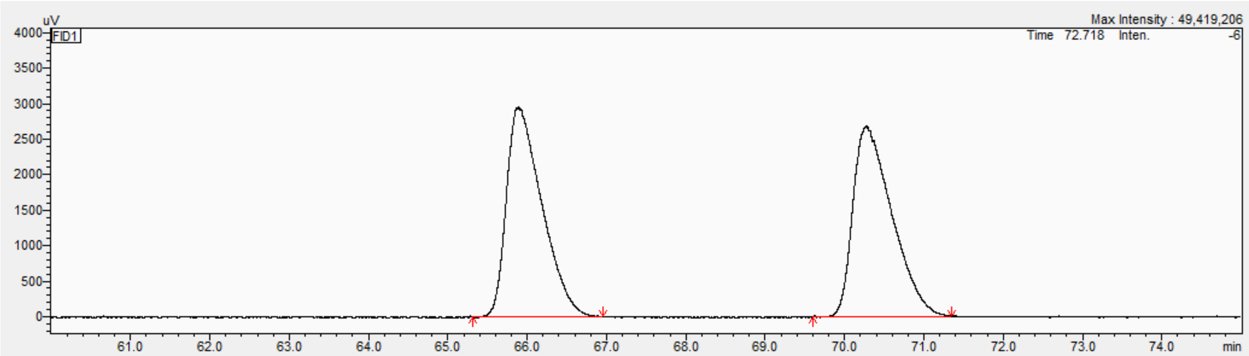

(a)

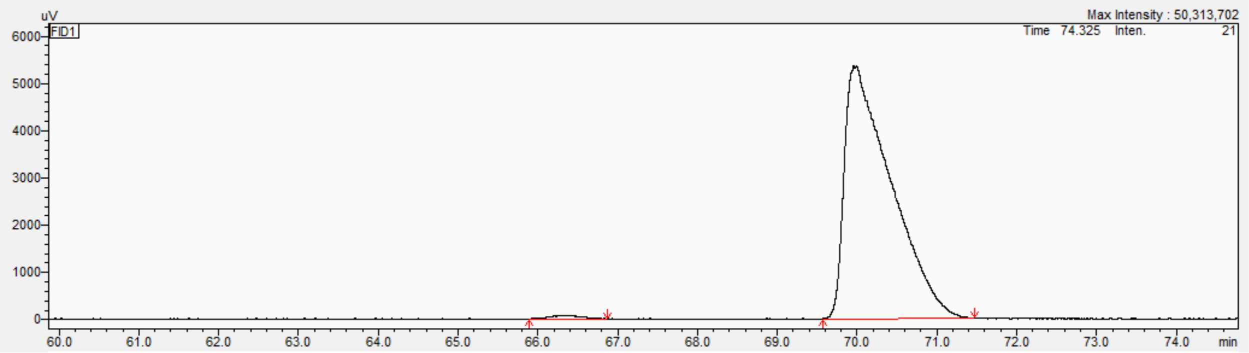

(b)

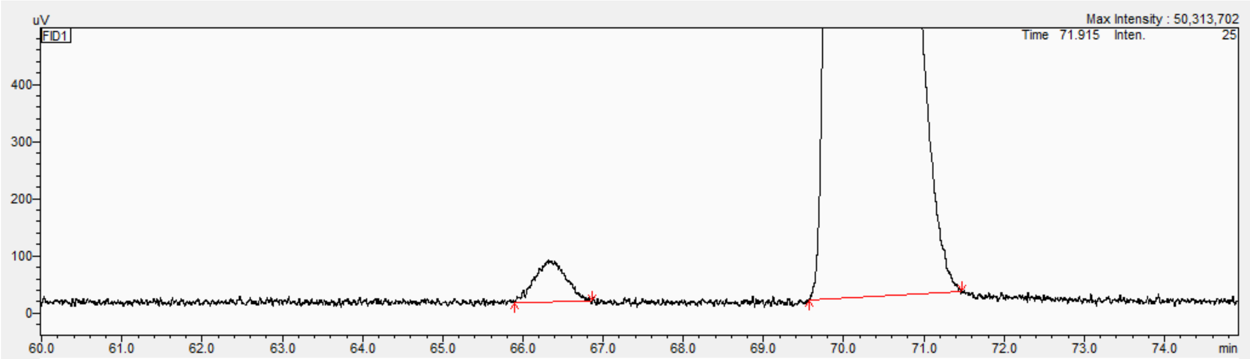

(c)

Figure S4. Chiral GC traces for 1-(4-bromophenyl)-ethanol: (a) racemic; (b) 98% e.e; (c) 98% e.e. zoomed in.

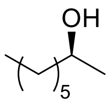

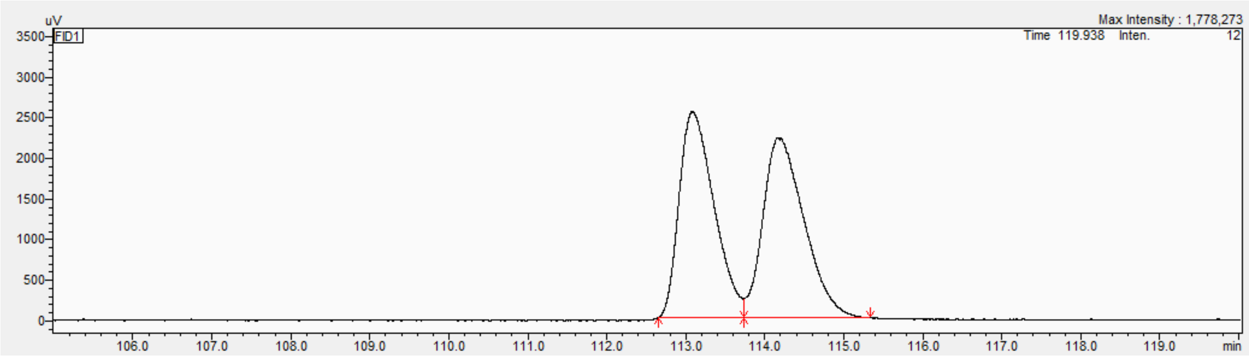

(a)

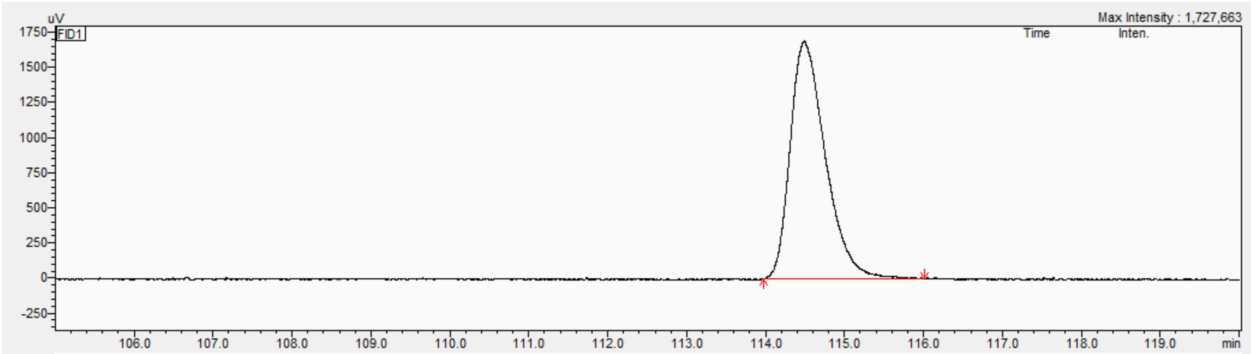

(b)

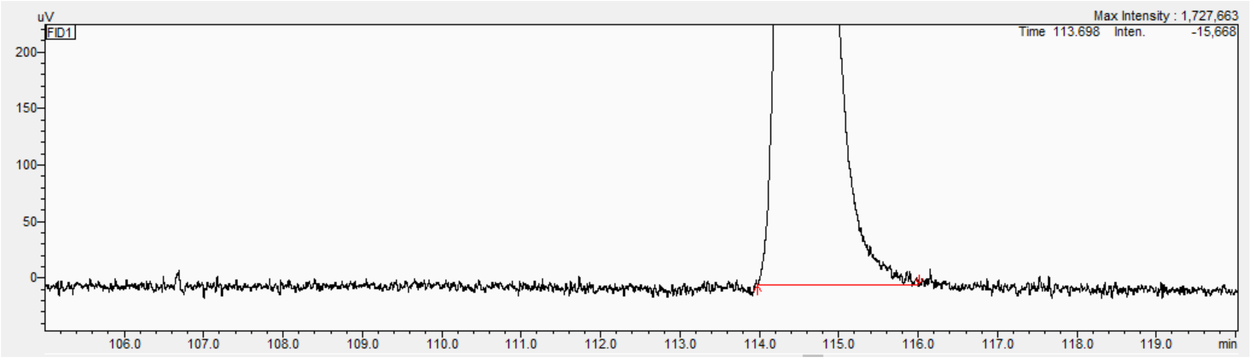

(c)

Figure S5. Chiral GC traces for 2-octanol: (a) racemic; (b) >99% e.e; (c) >99% e.e. zoomed in.

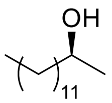

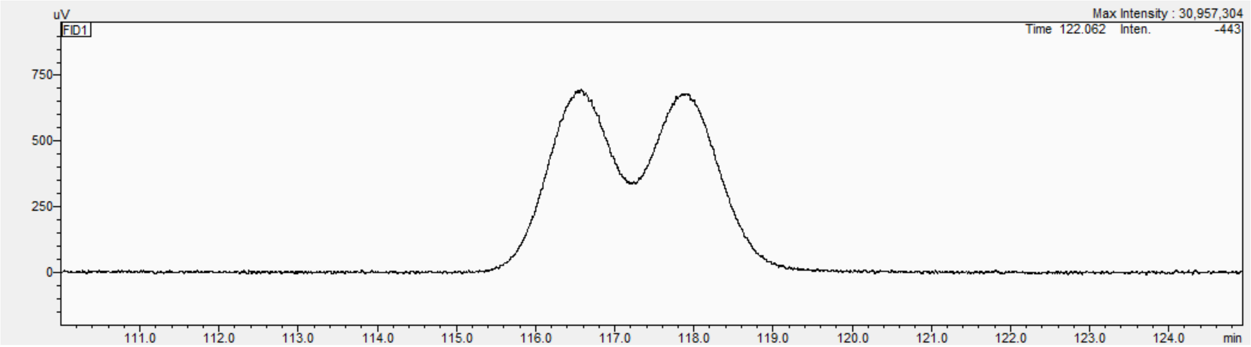

(a)

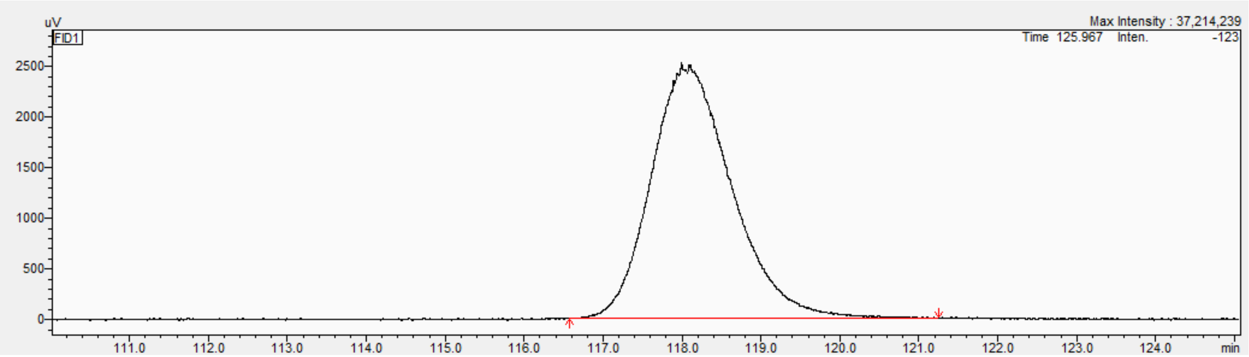

(b)

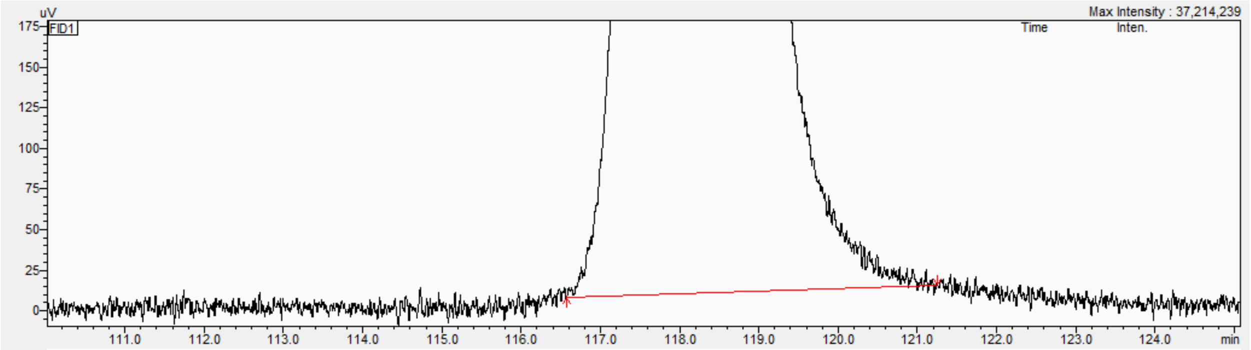

(c)

Figure S6. Chiral GC traces for 2-tetradecanol: (a) racemic; (b) >99% e.e; (c) >99% e.e. zoomed in.

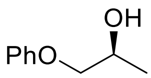

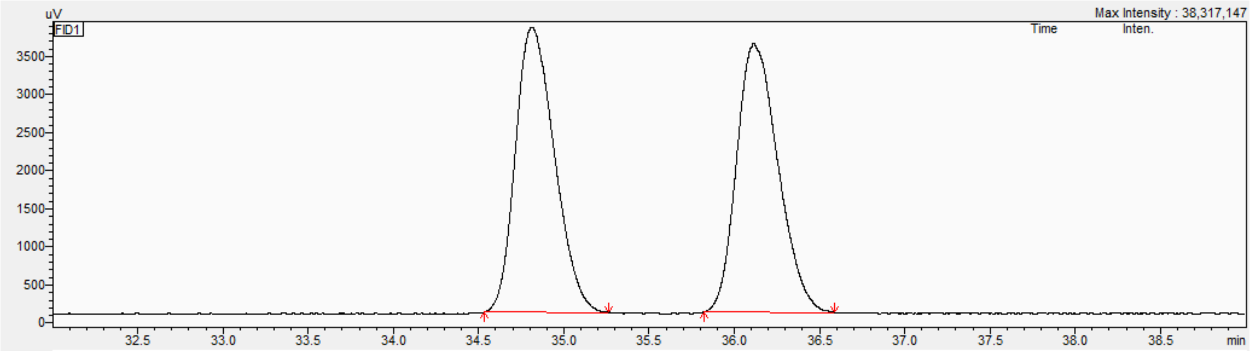

(a)

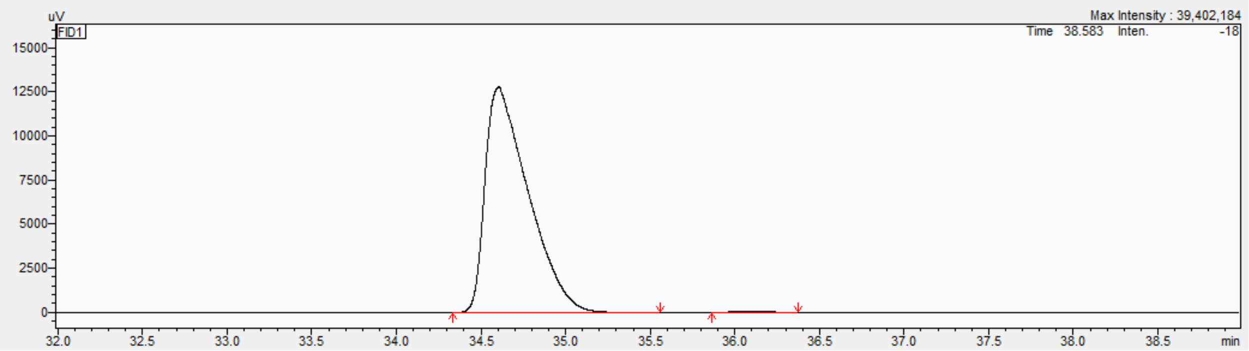

(b)

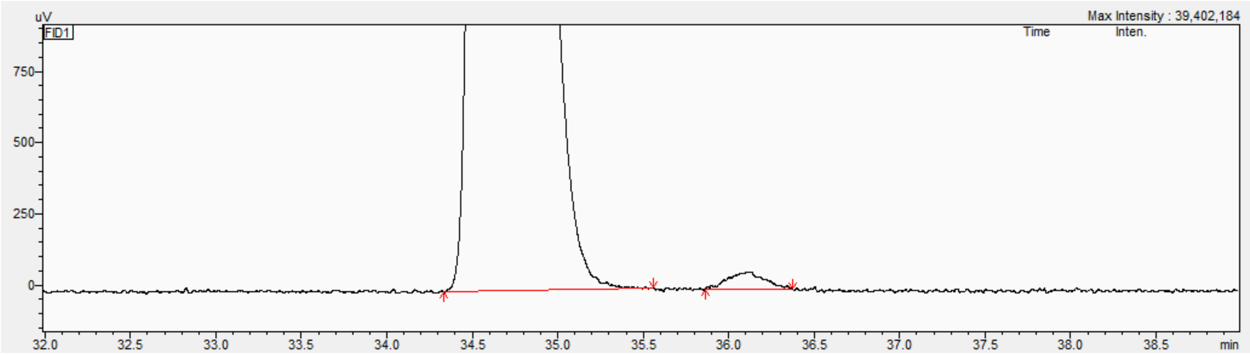

(c)

Figure S7. Chiral GC traces for 1-phenoxy-2-propanol: (a) racemic; (b) >99% e.e; (c) >99% e.e. zoomed in.

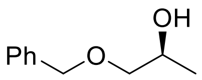

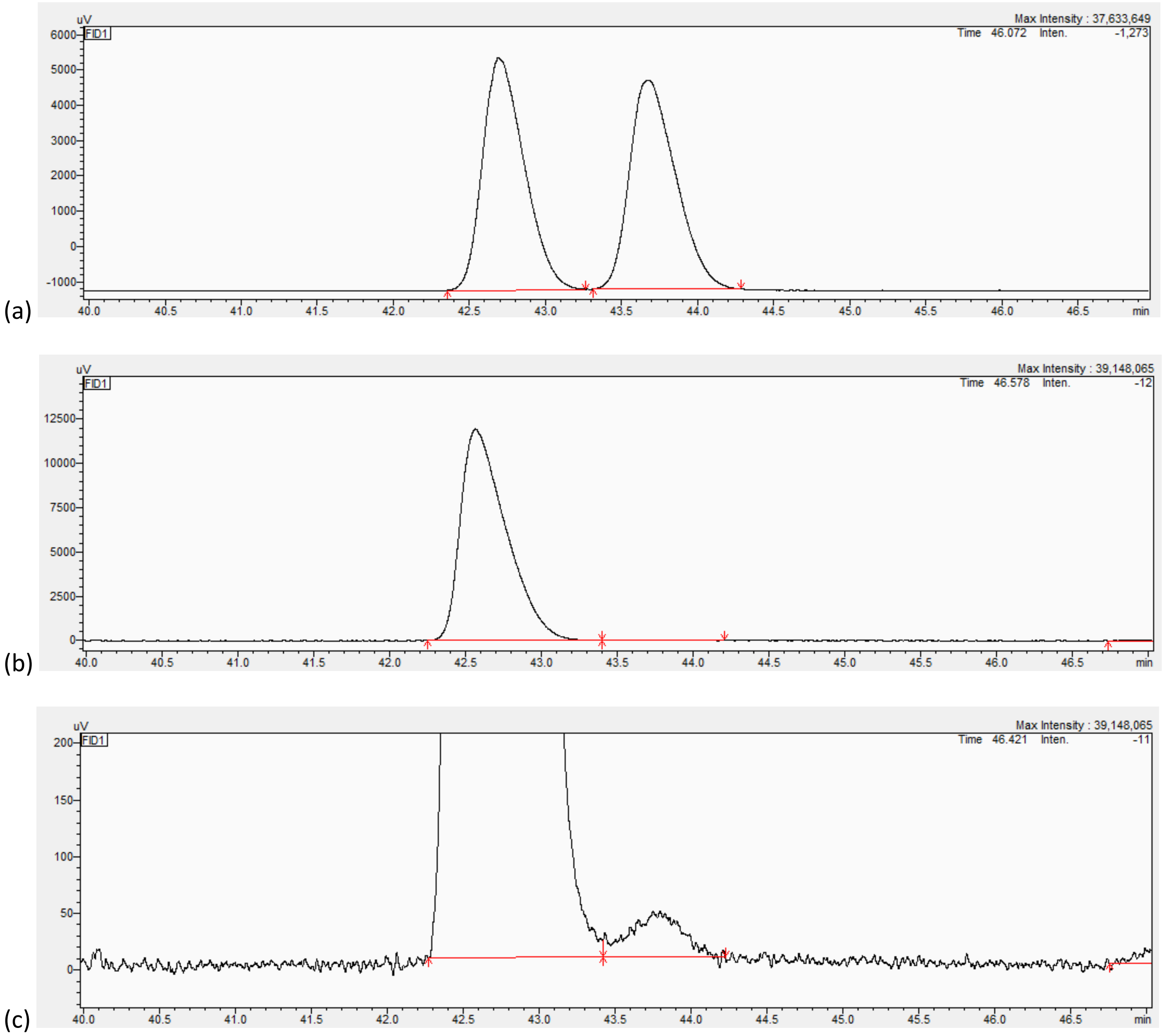

Figure S8. Chiral GC traces for 1-benzyloxy-2-propanol: (a) racemic; (b) >99% e.e; (c) >99% e.e. zoomed in.

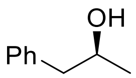

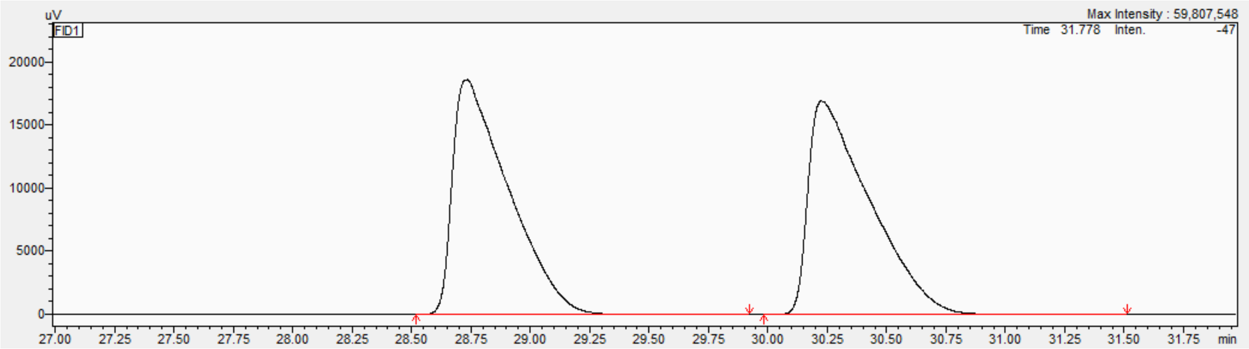

(a)

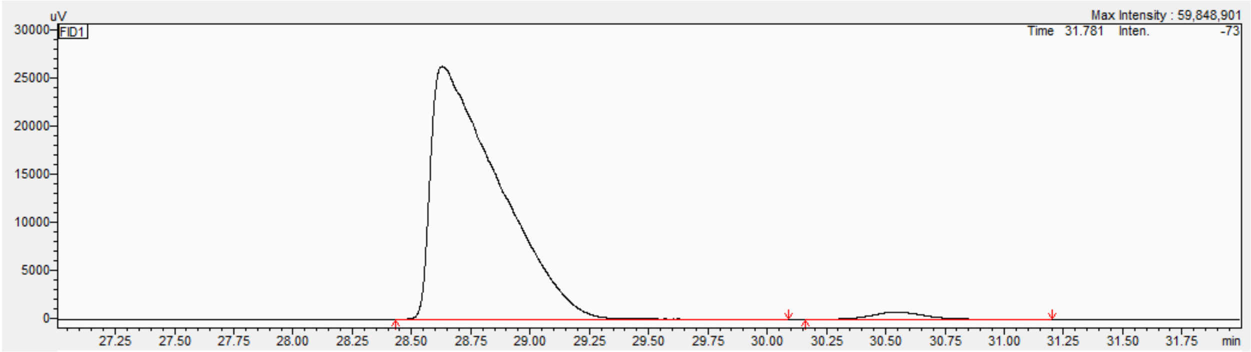

(b)

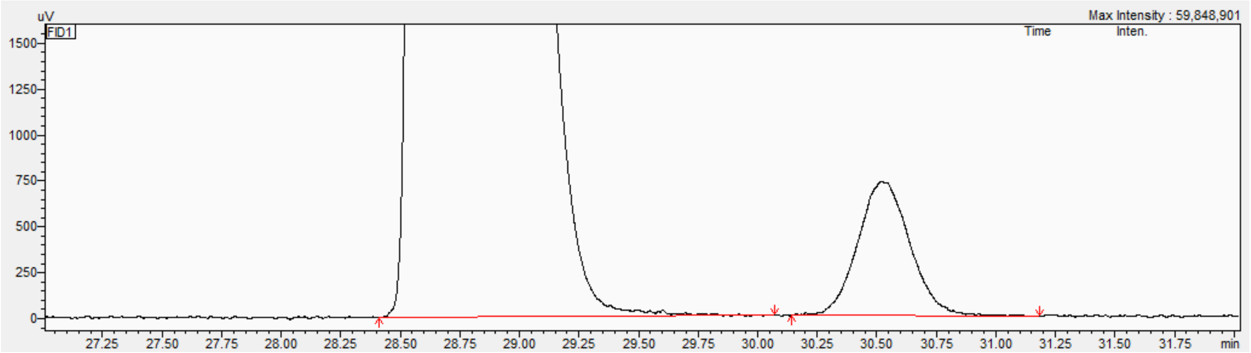

(c)

Figure S9. Chiral GC traces for 1-phenyl -2-propanol: (a) racemic; (b) 96% e.e; (c) 96% e.e. zoomed in.

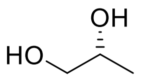

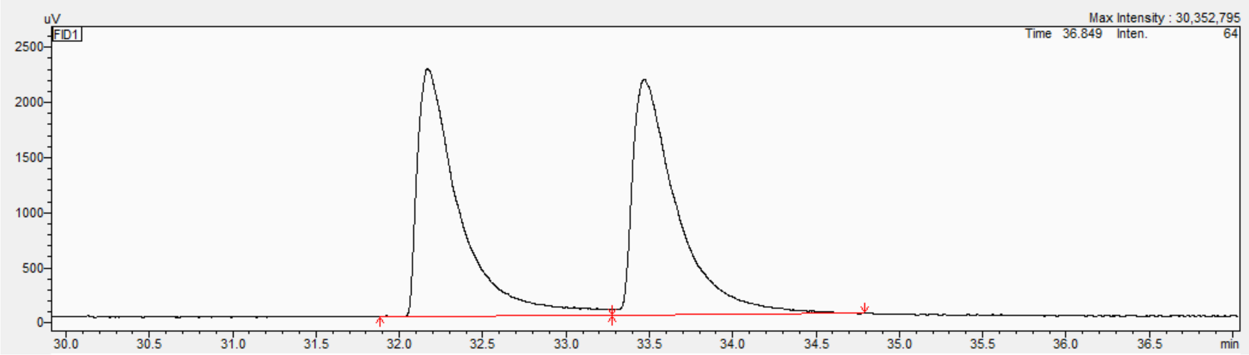

(a)

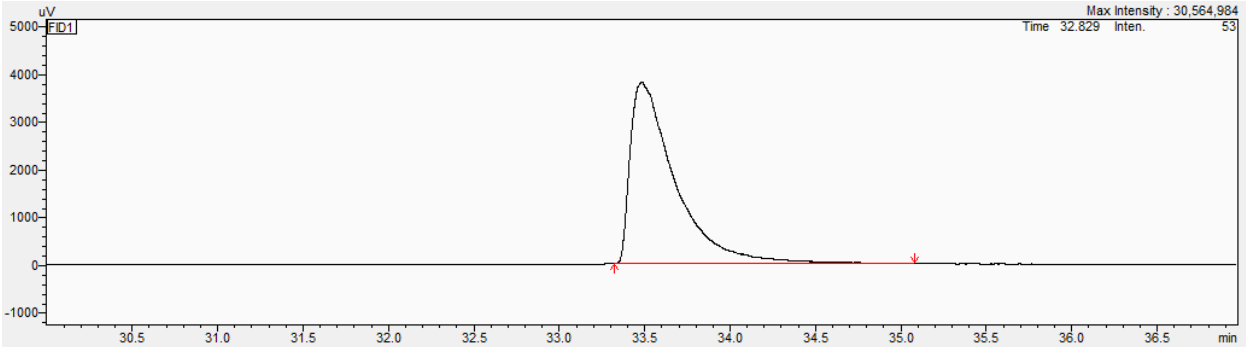

(b)

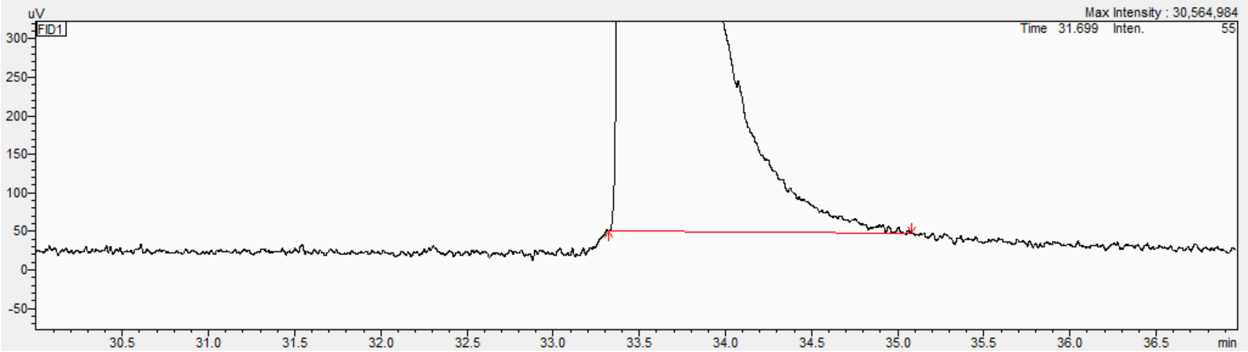

(c)

Figure S10. Chiral GC traces for 1,2-propanediol: (a) racemic; (b) >99% e.e; (c) >99% e.e. zoomed in.

NMR Spectra for Isolated Products of Epoxide Hydrogenolysis

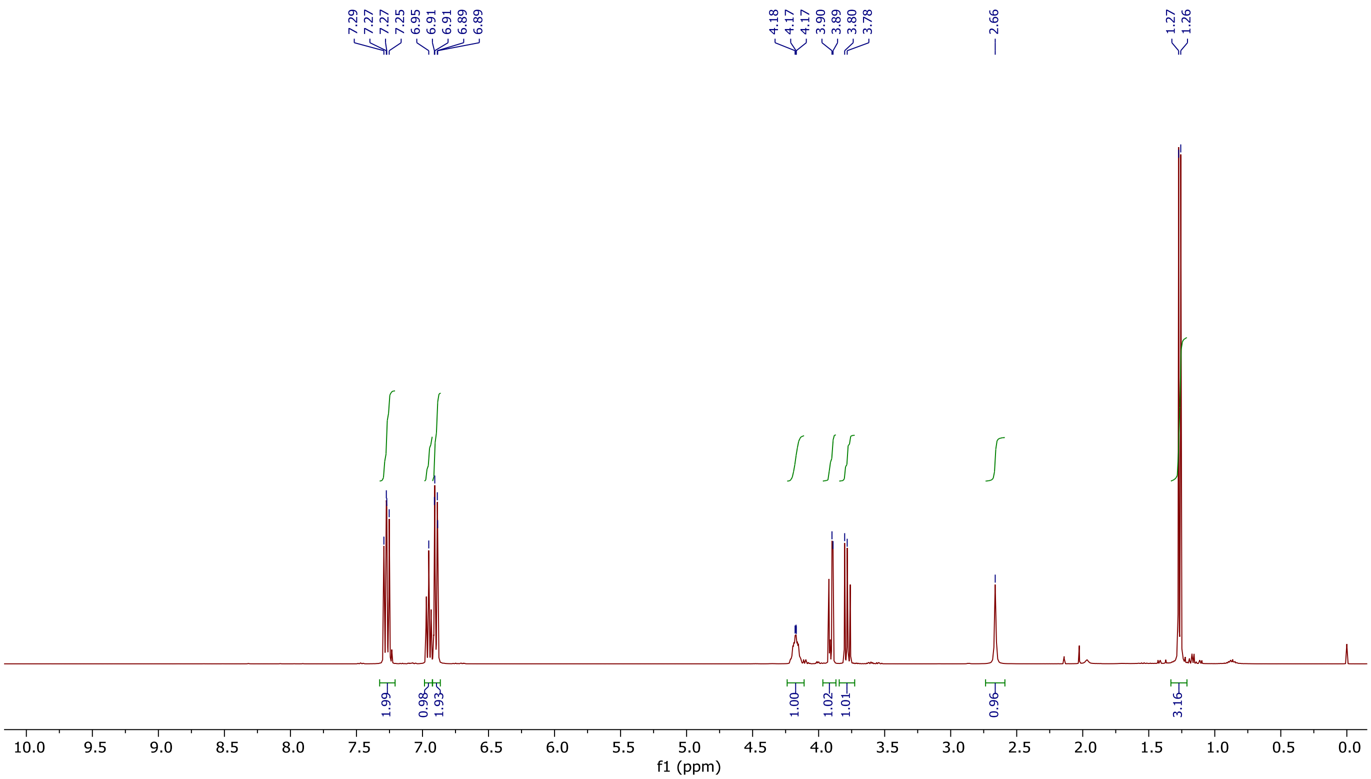

Figure S11. ^{1}H NMR spectrum of 1-phenoxypropan-2-ol.

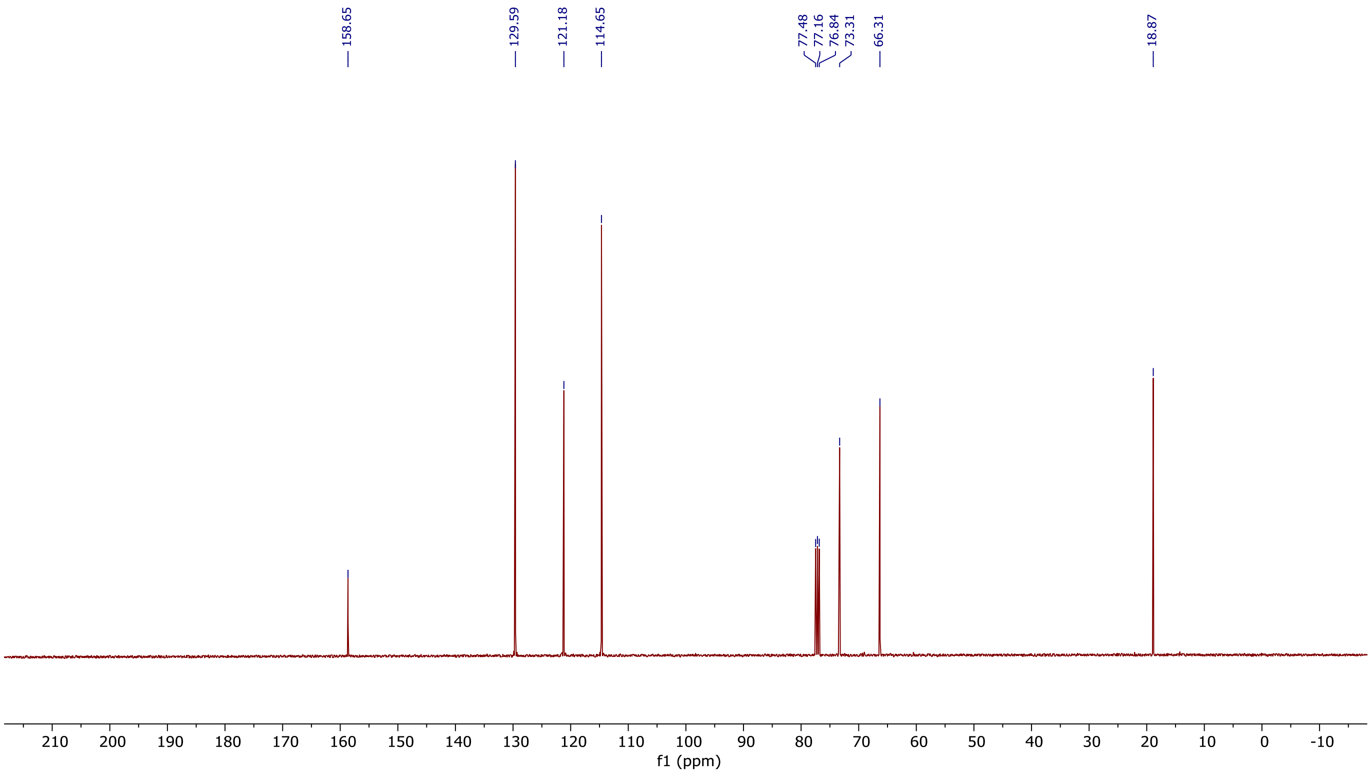

Figure S12. ^{13}C NMR spectrum of 1-phenoxypropan-2-ol.

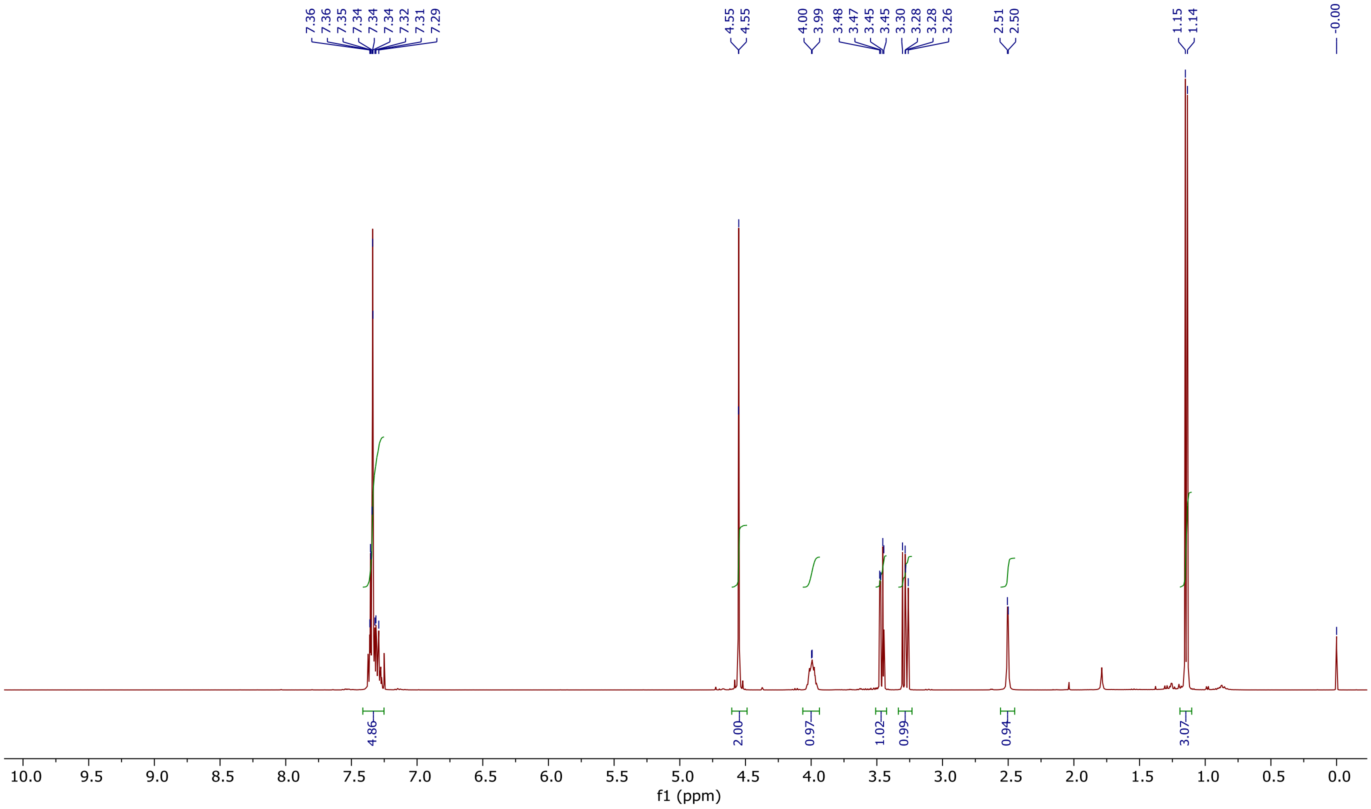

Figure S13. ^{1}H NMR spectrum of 1-benzyloxypropan-2-ol.

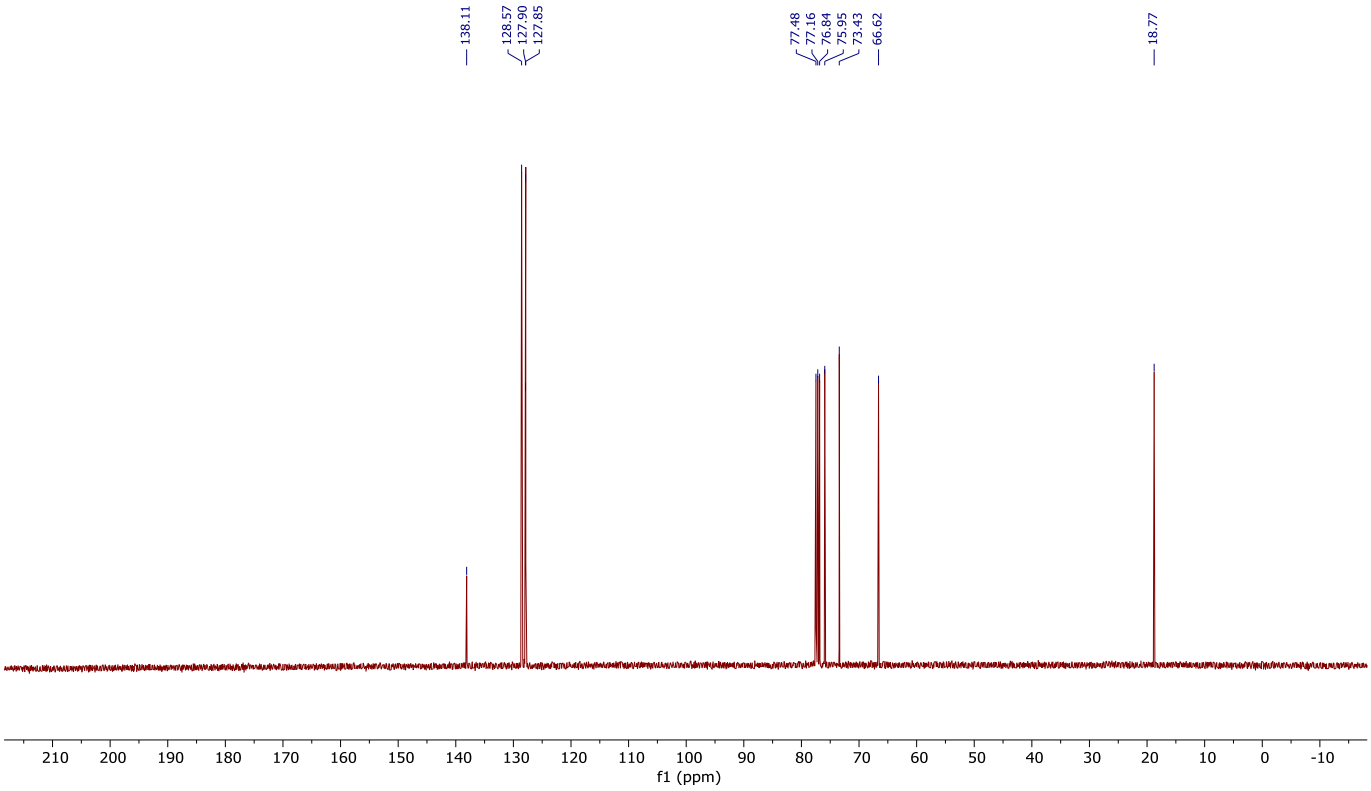

Figure S14. ^{13}C NMR spectrum of 1-benzyloxypropan-2-ol.

Energies Calculated by DFT

Table S3 below shows the energies calculated by DFT for all structures reported in this paper relevant to propylene oxide hydrogenolysis catalyzed by **RuCl** (Scheme 6, Figure 2, and Figures S15 – S18). The column **E(BS2)** represents the solvent-corrected electronic energy in hartrees, calculated with the ωB97X-D functional and the def2-TZVP basis set. The column **G(corr)** represents the correction to the Gibbs free energy calculated at 298.15 K after geometry optimization using the ωB97X-D functional and the def2-SVP basis set. The column **SS G (kcal)** represents the Gibbs free energy for each isolated species at 298.15 K in kcal/mol, including the addition of 1.89 kcal/mol for each molecule to convert to a 1 M standard state, except for the solvent 2-propanol, to which 3.42 kcal/mol are added to convert to the 13.08 M standard state. The column **Mass Balance** lists the small molecules included in the total free energy for the calculation of reaction pathways. The column **G(total, kcal)** is the sum of standard-state free energies of the ruthenium complex and any small molecules included for mass balance. The column **G(rel)** is the total free energy referenced against **RuH-solv**.

Table S10 (page S33) shows the energies calculated by DFT for the reversible dehydrogenation of 2-propanol to acetone, catalyzed by **$RuPNN^{HEt}$** (Figure 6). The DFT method is the same as that described above. The column **G(rel)** in Table S10 is the total free energy referenced against **p**, the 2-propanol hydrogen-bond adduct of **$RuPNN^{HEt}$**.

Table S3. Energies calculated by DFT for RuCl-catalyzed hydrogenolysis of propylene oxide.

Small Molecules	E(BS2)	G(corr)	SS G(kcal)
hydrogen	-1.1758287	-0.001509	-736.90
1-propanol	-194.3815412	0.081334	-121923.236
2-propanol	-194.3871279	0.081117	-121925.355
propylene-oxide	-193.1307677	0.059632	-121151.982

Resting states	E(BS2)	G(corr)	SS G(kcal)	Mass Balance	G(total, kcal)	G(rel)
RuH	-1405.636038	0.487909	-881741.20	2 * 2-propanol	-1125591.91	0.14
RuH-solv	-1600.035144	0.586226	-1003666.70	2-propanol	-1125592.05	0.00
Ru-OiPr	-1598.835132	0.572538	-1002922.27	2-propanol + H_2	-1125584.52	7.53
RuOiPr-solv	-1793.240530	0.674377	-1124849.50	H_2	-1125586.40	5.66
Ru-dearom	-1404.427308	0.465707	-880996.65	2 * 2-propanol + H_2	-1125584.25	7.80
Ru-dearom-solv	-1598.824899	0.563726	-1002921.38	2-propanol + H_2	-1125583.63	8.42
**Minimum Energy Pathway**						
RuOiPr-solv	-1793.240530	0.674377	-1124849.50	H_2 + epoxide	-1246738.38	5.66
a	-1793.223814	0.670041	-1124841.73	H_2 + epoxide	-1246730.61	13.42
b	-1794.409963	0.688564	-1125574.43	propylene-oxide	-1246726.41	17.63
c-TS	-1794.407626	0.687040	-1125573.92	propylene-oxide	-1246725.90	18.14
RuH-solv	-1600.035144	0.586226	-1003666.70	propylene-oxide + 2-propanol	-1246744.04	0.00
d	-1598.775593	0.565219	-1002889.50	2 * 2-propanol	-1246740.21	3.82
e-TS	-1598.741423	0.566987	-1002866.95	2 * 2-propanol	-1246717.66	26.38
f	-1598.809258	0.570983	-1002907.01	2 * 2-propanol	-1246757.72	-13.68
RuOiPr-solv	-1793.240530	0.674377	-1124849.50	2-propanol	-1246774.86	-30.82
**Ring Opening including explicit 2-propanol**						
g	-1793.175663	0.666980	-1124813.44	2-propanol	-1246738.79	5.24
h-TS	-1793.143716	0.669165	-1124792.02	2-propanol	-1246717.38	26.66
i	-1793.218584	0.672545	-1124836.88	2-propanol	-1246762.23	-18.20
**Hydrogen activation with only one explicit 2-propanol**						
j	-1599.998313	0.587194	-1003642.98	propylene-oxide + 2-propanol	-1246720.32	23.72
k-TS	-1599.997062	0.587035	-1003642.29	propylene-oxide + 2-propanol	-1246719.63	24.40
RuH-solv	-1600.035144	0.586226	-1003666.70	propylene-oxide + 2-propanol	-1246744.04	0.00
**Hydrogen activation through NCH_2 linker**						
l	-1599.997449	0.585994	-1003643.19	2 * 2-propanol	-1246720.53	23.51
m-TS	-1599.987113	0.582245	-1003639.06	2 * 2-propanol	-1246716.39	27.64
RuH-solv	-1600.035144	0.586226	-1003666.70	2 * 2-propanol	-1246744.04	0.00
**Hydrogen activation through PCH_2 linker**						
n	-1600.010547	0.586906	-1003650.84	2 * 2-propanol	-1246728.17	15.86
o-TS	-1599.991509	0.583331	-1003641.13	2 * 2-propanol	-1246718.47	25.56
RuH-solv	-1600.035144	0.586226	-1003666.70	2 * 2-propanol	-1246744.04	0.00

Alternative Pathway: Ring Opening Including Explicit 2-Propanol

Figure S15 below shows the calculated pathway for epoxide ring-opening, including an explicit solvent molecule to stabilize the developing negative charge on the epoxide oxygen. The barrier of 26.7 kcal/mol is slightly higher than the corresponding barrier of 26.4 kcal/mol in the MEP.

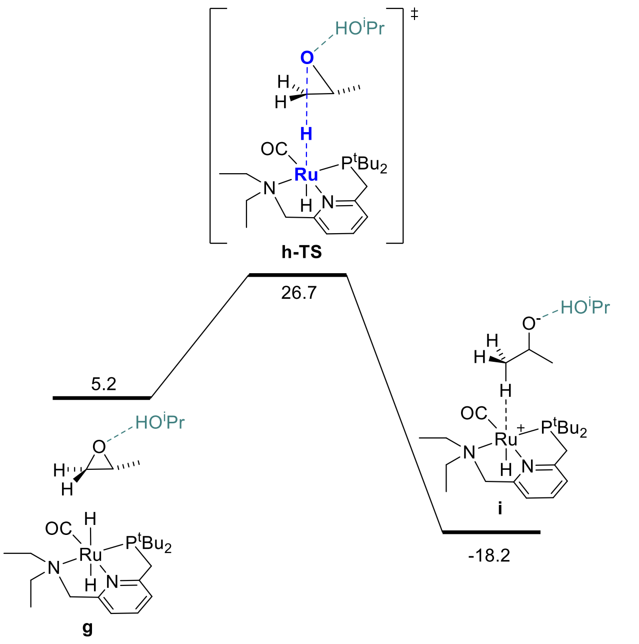

Figure S15. Ring opening including explicit 2-propanol.

Alternative Pathway: Hydrogen Activation Without Explicit 2-Propanol

Figure S16 below shows the calculated pathway for hydrogen activation mediated by the isopropoxide anion, omitting the explicit solvent molecule in the corresponding sequence in the MEP. The barrier of 24.4 kcal/mol is significantly higher than the corresponding barrier of 18.1 kcal/mol in the MEP.

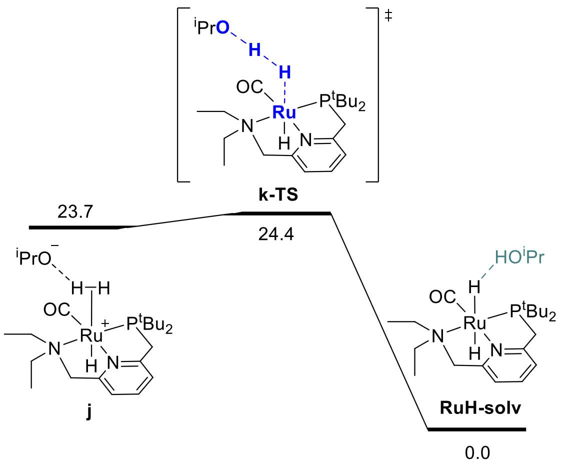

Figure S16. Hydrogen activation without explicit 2-propanol.

Alternative Pathway: Hydrogen Activation Through the NCH_2 Linker

Figure S17 below shows the calculated pathway for hydrogen activation mediated by a deprotonated CH_2 linker connected to the NEt_2 group, employing a molecule of 2-propanol as proton shuttle. The barrier of 27.6 kcal/mol is significantly higher than the corresponding barrier of 18.1 in the MEP.

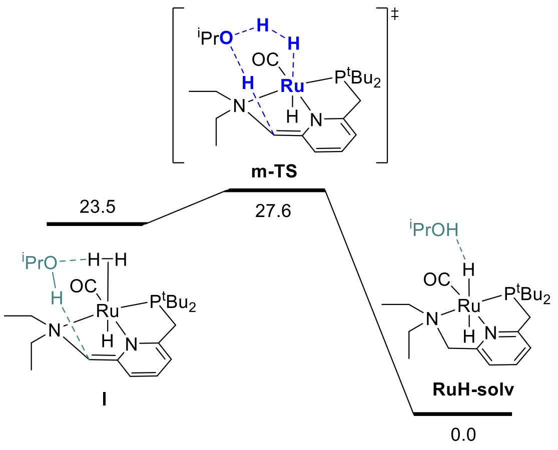

Figure S17. Hydrogen activation through the NCH_2 linker.

Alternative Pathway: Hydrogen Activation Through the PCH_2 Linker

Figure S18 below shows the calculated pathway for hydrogen activation mediated by a deprotonated CH_2 linker connected to the P^tBu_2 group, employing a molecule of 2-propanol as proton shuttle. The barrier of 25.6 kcal/mol is significantly higher than the corresponding barrier of 18.1 in the MEP.

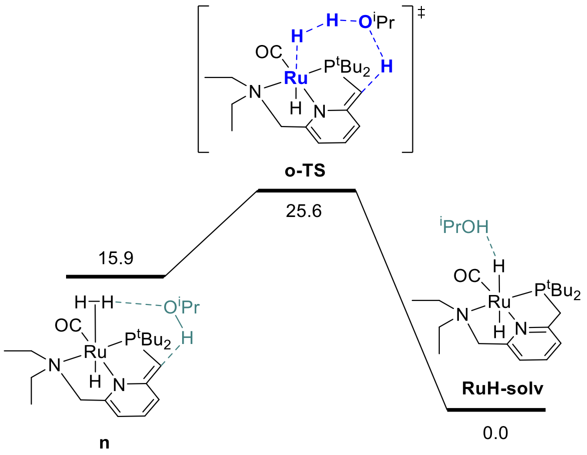

Figure S18. Hydrogen activation through the PCH_2 linker.

Derivation of the Rate Law for Catalytic Epoxide Hydrogenolysis

Because both **RuO^{i}Pr** and **RuH** occupy a significant fraction of the ruthenium speciation under catalytic conditions, saturation kinetics are expected, as described below. In the following derivation, we have assumed that the hydrogenolysis product 2-tetradecanol will interact with the ruthenium species similarly to the 2-propanol solvent. Since the solvent at 13.08 M is always at a much higher concentration than the product, we have ignored potential inhibition by the 2-tetradecanol product in the kinetic analysis.

In the analysis of experimental data, as well as in the DFT calculations, we have taken the standard state of the reacting species **RuH**, **RuO^{i}Pr**, epoxide, and H_2 to be 1.0 M in solution. The solvent isopropyl alcohol has a standard state defined by its neat molarity of 13.08 M.

The two-step sequence in Scheme 7 can be written in linear form as:

$$1)\ \mathrm{RuO^iPr} + \mathrm{H_2} \underset{\mathrm{k_{-1}}}{\overset{\mathrm{k_1}}{\rightleftharpoons}} \mathrm{RuH} + {}^{\mathrm{i}}\mathrm{PrOH}$$

$$2)\ \mathrm{RuH} + \mathrm{epoxide} \xrightarrow{\mathrm{k_2}} \mathrm{RuO^iPr} + \mathrm{product}$$

If **RuO^{i}Pr** and **RuH** are the dominant ruthenium species present, we can represent the sum of their concentrations as $[\mathrm{Ru}]_{\mathrm{total}}$:

$$[\mathrm{Ru}]_{\mathrm{total}} = [\mathrm{RuH}] + [\mathrm{RuO^iPr}]$$

We can apply the steady-state approximation to the **RuH** intermediate:

$$\frac{d[\mathrm{RuH}]}{dt} = 0 = k_1[\mathrm{RuO^iPr}]\,[\mathrm{H_2}] - k_{-1}[\mathrm{RuH}][{}^{i}\mathrm{PrOH}] - k_2[\mathrm{RuH}][\mathrm{epoxide}]$$

Substituting ($[\mathrm{Ru}]_{\mathrm{total}}$ - [RuH]) for [RuO^{i}Pr] gives:

$$0 = k_1([\mathrm{Ru}]_{\mathrm{total}} - [\mathrm{RuH}])\,[\mathrm{H_2}] - k_{-1}[\mathrm{RuH}][{}^{i}\mathrm{PrOH}] - k_2[\mathrm{RuH}][\mathrm{epoxide}]$$

Solving for [RuH] gives:

$$[\mathrm{RuH}] = \frac{k_1[\mathrm{Ru}]_{\mathrm{total}}[\mathrm{H_2}]}{k_1[\mathrm{H_2}] + k_{-1}[{}^{i}\mathrm{PrOH}] + k_2[\mathrm{epoxide}]}$$

From reaction 2 above, the rate of product formation (the negative of the rate of epoxide consumption) is:

$$\frac{dP}{dt} = k_2[\mathrm{RuH}][\mathrm{epoxide}]$$

Substituting in the steady-state concentration of RuH gives:

$$\frac{dP}{dt} = \frac{k_1 k_2[\mathrm{Ru}]_{\mathrm{total}}[\mathrm{H_2}][\mathrm{epoxide}]}{k_1[\mathrm{H_2}] + k_{-1}[{}^{i}\mathrm{PrOH}] + k_2[\mathrm{epoxide}]}$$

Here, we can make a simplifying assumption that $k_2[\text{epoxide}]$ is negligible in the denominator. If $k_2[\text{epoxide}]$ were significant, saturation behavior in [epoxide] would be observed at high epoxide concentrations. This assumption is also consistent with the energy barriers predicted by DFT. The barrier from **RuH-solv** to **e-TS** (corresponding to k_2) is 26.4 kcal/mol, while the barrier from **RuH-solv** backwards to **c-TS^{PNN}** (corresponding to k_{-1}) is 18.1 kcal/mol. This assumption gives the rate law:

$$\frac{dP}{dt} = \frac{k_2[\text{Ru}]_{\text{total}}[H_2][\text{epoxide}]}{[H_2] + \frac{k_{-1}}{k_1}[{}^i\text{PrOH}]}$$

At this point, reaction 1 is a fast pre-equilibrium and reaction 2 is rate-determining. We can replace k_{-1}/k_1 with the reciprocal of the equilibrium constant K_1 for reaction 1. Since the thermodynamic equilibrium constant K_1 is calculated using the standard state of 13.08 M for iPrOH (see below), the term $(k_{-1}/k_1)[{}^i\text{PrOH}]$ is equal to $1/K_1$.

$$\frac{dP}{dt} = \frac{k_2[\text{Ru}]_{\text{total}}[H_2][\text{epoxide}]}{[H_2] + \frac{1}{K_1}}$$

Determination of the Equilibrium Constant K_1 by NMR Spectroscopy

The equilibrium between **RuO^{i}Pr** and **RuH** was analyzed by NMR spectroscopy in the absence of epoxide. In the glovebox, a stock solution was prepared in non-deuterated isopropyl alcohol containing 0.0137 M **RuCl** and 0.20 M KO^{t}Bu. After stirring for five minutes, the solution was filtered, and 0.70 mL was transferred to a J. Young NMR tube. The NMR tube was removed from the box and hydrogen gas was added, after which the NMR tube was sealed and shaken. Thirteen such samples were prepared, with hydrogen pressures ranging from 0.2 bar to 5.6 bar. For each sample, an unlocked ^{1}H NMR spectrum was recorded, using a long delay between scans of 20 seconds to allow for accurate integration. The relative integrations for the H_2 signal at 5.03 ppm, the **RuH** signal at -4.90 ppm, and the **RuO^{i}Pr** signal at -15.70 ppm were used to calculate the molarities of the three species. Table S4 below summarizes this data.

Table S4. NMR Measurements of the **RuH**/**RuO^{i}Pr** equilibrium.

**Integrations (arbitrary units)**			**Concentrations (M)**				
H_2	RuH	RuOiPr	[H_2]	[RuH]	[RuO^{i}Pr]	P(H_2) (bar)	[RuH]/[Ru]$_{tot}$
150076	112142	1433690	0.00069	0.00052	0.01318	0.198	0.0376
194292	307497	1535480	0.00079	0.00125	0.01245	0.226	0.0910
203705	247428	1583980	0.00082	0.00099	0.01271	0.235	0.0724
562829	622694	1378040	0.00228	0.00252	0.01118	0.655	0.1843
717083	762773	1186330	0.00313	0.00333	0.01037	0.900	0.2433
778608	1073590	1119580	0.00322	0.00444	0.00926	0.924	0.3241
1107330	958163	1333740	0.00418	0.00362	0.01008	1.20	0.2643
1090390	1072640	1103780	0.00455	0.00448	0.00922	1.31	0.3270
1974910	1428970	911777	0.00832	0.00602	0.00768	2.39	0.4393
2483920	1560280	734882	0.01123	0.00705	0.00665	3.22	0.5149
4005640	1923490	710453	0.01641	0.00788	0.00582	4.71	0.5751
3338510	1353440	518467	0.01913	0.00776	0.00594	5.49	0.5662
3426820	1340410	538230	0.01942	0.00760	0.00610	5.58	0.5546

For the equilibrium between **RuO^{i}Pr** and **RuH** (reaction 1 on page S25), the thermodynamic equilibrium constant K_1 is defined as follows:

$$K_1 = \frac{[\mathrm{RuH}][{}^i\mathrm{PrOH}]}{[\mathrm{RuO^iPr}][\mathrm{H_2}]} \div \frac{(1\ \mathrm{M})(13.08\ \mathrm{M})}{(1\ \mathrm{M})(1\ \mathrm{M})}$$

Assuming that the only ruthenium species present are **RuH** and **RuO^{i}Pr**, we can solve for [RuO^{i}Pr]:

$$[\mathrm{Ru}]_{\mathrm{total}} = [\mathrm{RuH}] + [\mathrm{RuO^iPr}]$$

$$[\mathrm{RuO^iPr}] = [\mathrm{Ru}]_{\mathrm{total}} - [\mathrm{RuH}]$$

We can then substitute this expression for [RuO^{i}Pr] into the equilibrium equation:

$$K_1 = \frac{[\mathrm{RuH}][{}^i\mathrm{PrOH}]}{([\mathrm{Ru_{total}}] - [\mathrm{RuH}])[\mathrm{H_2}]} \div \frac{(1\ \mathrm{M})(13.08\ \mathrm{M})}{(1\ \mathrm{M})(1\ \mathrm{M})}$$

Solving for [RuH]/[Ru]$_{total}$ gives:

$$\frac{[\mathrm{RuH}]}{[\mathrm{Ru_{total}}]} = \frac{K_1[\mathrm{H_2}]}{[{}^i\mathrm{PrOH}]/13.08 + K_1[\mathrm{H_2}]} = \frac{K_1[\mathrm{H_2}]}{1 + K_1[\mathrm{H_2}]}$$

Plotting [RuH]/[Ru]$_{total}$ vs [H_2] allows K_1 to be determined through a least-squares fit, as shown in Figure 1 in the main text. K_1 was determined to be 89 ± 6, corresponding to $\Delta G°$ = -2.66 ± 0.04 kcal/mol at 298.15 K.

Concentrations Measured in Kinetic Experiments

Table S5. Concentration data from kinetic experiments in Figure 3. Concentrations are in moles/liter.

Standard Conditions: [Ru] = 0.0050 M, $[epoxide]_0$ = 0.250 M, $P(H_2)$ = 20 bar, $[KO^iPr]$ = 0.01875 M

t (s)	[epoxide]	[product]	t (s)	[epoxide]	[product]	t (s)	[epoxide]	[product]	t (s)	[epoxide]	[product]
0	0.250	0.000	0	0.250	0.000	0	0.250	0.000	0	0.250	0.000
1020	0.240	0.010	1020	0.247	0.003	900	0.247	0.003	1920	0.230	0.019
1920	0.227	0.023	1920	0.243	0.007	1800	0.238	0.012	3720	0.211	0.037
2820	0.213	0.035	2820	0.234	0.016	2700	0.226	0.023	5520	0.191	0.057
3720	0.201	0.047	3720	0.222	0.026	3600	0.212	0.036	7320	0.173	0.074
4920	0.185	0.062	4920	0.208	0.040	4800	0.196	0.052	9120	0.154	0.091
6120	0.171	0.076	6120	0.195	0.055	6000	0.181	0.067	10920	0.138	0.107
7320	0.156	0.089	7320	0.179	0.068	7200	0.166	0.081	12720	0.124	0.122
9120	0.138	0.108	9120	0.161	0.086	9000	0.146	0.100	14520	0.111	0.134
10920	0.122	0.125	10920	0.142	0.104	10800	0.129	0.116			
12720	0.107	0.139	12720	0.125	0.119	12600	0.114	0.132			
14520	0.094	0.153	14520	0.111	0.133	14400	0.099	0.146			

Variation of [Ru]:

0.0025 M Ru			0.0037 M Ru			0.0063 M Ru			0.0075 M Ru		
t (s)	[epoxide]	[product]	t (s)	[epoxide]	[product]	t (s)	[epoxide]	[product]	t (s)	[epoxide]	[product]
0	0.250	0.000	0	0.250	0.000	0	0.250	0.000	0	0.250	0.000
900	0.250	0.000	960	0.245	0.005	900	0.241	0.009	1800	0.224	0.026
1800	0.247	0.003	1860	0.237	0.012	1800	0.225	0.024	3600	0.192	0.056
2700	0.241	0.008	2760	0.228	0.020	2700	0.208	0.039	5400	0.160	0.088
3600	0.236	0.014	3660	0.221	0.027	3600	0.196	0.054	7200	0.132	0.115
4800	0.228	0.021	4860	0.210	0.038	4800	0.174	0.072	9000	0.109	0.137
6000	0.220	0.028	6060	0.200	0.046	6000	0.157	0.089	10800	0.090	0.156
7200	0.212	0.035	7260	0.190	0.056	7200	0.141	0.104	12600	0.075	0.172
9000	0.201	0.045	9060	0.178	0.068	9000	0.120	0.124	14400	0.062	0.184
10800	0.191	0.055	10860	0.164	0.080	10800	0.102	0.142			
12600	0.181	0.064	12660	0.152	0.093	12600	0.087	0.157			
14400	0.172	0.073	14460	0.138	0.106	14400	0.074	0.170			

Variation of $[epoxide]_0$:

0.125 M epoxide			0.375 M epoxide		
t (s)	[epoxide]	[product]	t (s)	[epoxide]	[product]
0	0.125	0.000	0	0.375	0.000
960	0.123	0.002	1080	0.364	0.010
1860	0.120	0.005	1980	0.350	0.024
2760	0.115	0.009	2880	0.334	0.040
3660	0.109	0.015	3780	0.317	0.056
4860	0.101	0.023	4980	0.295	0.076
6060	0.093	0.031	6180	0.276	0.094
7260	0.084	0.039	7380	0.255	0.114
9060	0.074	0.049	9180	0.229	0.140
10860	0.065	0.058	10980	0.204	0.164
12660	0.058	0.066	12780	0.181	0.185
14460	0.050	0.073	14580	0.162	0.206

Table S5 continued.

Variation of $P(H_2)$:

10 bar		
t (s)	[epoxide]	[product]
0	0.250	0.000
900	0.245	0.005
1800	0.236	0.013
2700	0.227	0.022
3600	0.216	0.032
4800	0.203	0.044
6000	0.191	0.056
7200	0.178	0.068
9000	0.161	0.084
10800	0.145	0.099
12600	0.131	0.113
14400	0.119	0.126

30 bar		
t (s)	[epoxide]	[product]
0	0.250	0.000
900	0.245	0.005
1800	0.233	0.016
2700	0.220	0.029
3600	0.207	0.041
4800	0.190	0.057
6000	0.175	0.071
7200	0.162	0.084
9000	0.143	0.103
10800	0.126	0.119
12600	0.111	0.134
14400	0.098	0.146

30 bar		
t (s)	[epoxide]	[product]
0	0.250	0.000
1800	0.236	0.014
3600	0.209	0.040
5400	0.183	0.064
7200	0.159	0.087
9000	0.139	0.108
10800	0.121	0.125
12600	0.105	0.140
14400	0.092	0.154

30 bar		
t (s)	[epoxide]	[product]
0	0.250	0.000
1860	0.224	0.025
3660	0.195	0.052
5460	0.169	0.078
7260	0.147	0.099
9060	0.128	0.118
10860	0.111	0.135
12660	0.096	0.149
14460	0.083	0.162

Variation of $[KO^iPr]$:

0.0063 M KO^iPr		
t (s)	[epoxide]	[product]
0	0.250	0.000
1860	0.246	0.004
3660	0.235	0.014
5460	0.217	0.031
7260	0.199	0.048
9060	0.181	0.065
10860	0.164	0.081
12660	0.150	0.095
14460	0.136	0.109

0.0125 M KO^iPr		
t (s)	[epoxide]	[product]
0	0.250	0.000
1920	0.231	0.018
3720	0.211	0.038
5520	0.191	0.058
7320	0.172	0.076
9120	0.154	0.093
10920	0.138	0.108
12720	0.124	0.123
14520	0.110	0.136

0.0125 M KO^iPr		
t (s)	[epoxide]	[product]
0	0.250	0.000
960	0.247	0.003
1860	0.240	0.011
2760	0.229	0.021
3660	0.217	0.031
4860	0.204	0.044
6060	0.190	0.057
7260	0.178	0.069
9060	0.161	0.086
10860	0.145	0.101
12660	0.130	0.115
14460	0.117	0.128

0.0250 M KO^iPr		
t (s)	[epoxide]	[product]
0	0.250	0.000
1080	0.236	0.013
1980	0.222	0.026
2880	0.208	0.040
3780	0.194	0.053
4980	0.177	0.069
6180	0.162	0.084
7380	0.148	0.097
9180	0.129	0.116
10980	0.111	0.132
12780	0.097	0.147
14580	0.083	0.160

Table S6. Concentration data from kinetic experiments in Figure 4 with added [2.2.2]cryptand. Concentrations are in moles/liter.

Addition of [2.2.2]cryptand, conditions otherwise standard

0.0187 M [2.2.2]cryptand			0.0187 M [2.2.2]cryptand			0.0187 M [2.2.2]cryptand			0.0375 M [2.2.2]cryptand		
t (s)	[epoxide]	[product]	t (s)	[epoxide]	[product]	t (s)	[epoxide]	[product]	t (s)	[epoxide]	[product]
0	0.250	0.000	0	0.250	0.000	0	0.250	0.000	0	0.250	0.000
960	0.246	0.004	900	0.246	0.004	1080	0.248	0.002	960	0.244	0.006
1860	0.238	0.010	1800	0.238	0.011	1980	0.244	0.005	1860	0.235	0.013
2760	0.230	0.017	2700	0.231	0.017	2880	0.237	0.010	2760	0.228	0.020
3660	0.221	0.025	3600	0.222	0.024	3780	0.229	0.017	3660	0.220	0.027
4860	0.210	0.033	4800	0.212	0.033	4980	0.219	0.026	4860	0.210	0.035
6060	0.200	0.042	6000	0.202	0.041	6180	0.208	0.034	6060	0.200	0.043
7260	0.190	0.051	7200	0.192	0.049	7380	0.199	0.042	7260	0.191	0.051
9060	0.176	0.062	9000	0.180	0.060	9180	0.185	0.054	9060	0.177	0.062
10860	0.164	0.073	10800	0.168	0.070	10980	0.172	0.065	10860	0.166	0.072
12660	0.151	0.083	12600	0.156	0.080	12780	0.160	0.075	12660	0.155	0.082
14460	0.140	0.092	14400	0.146	0.089	14580	0.150	0.084	14460	0.144	0.090

0.0750 M [2.2.2]cryptand		
t (s)	[epoxide]	[product]
0	0.250	0.000
1080	0.242	0.008
1980	0.238	0.012
2880	0.230	0.018
3780	0.222	0.025
4980	0.211	0.034
6180	0.200	0.042
7380	0.191	0.050
9180	0.178	0.062
10980	0.166	0.071
12780	0.154	0.081
14580	0.143	0.091

Table S7. Concentration data from kinetic experiments in Figure 4 with NaO^{i}Pr as base and added [2.2.2]cryptand. Concentrations are in moles/liter.

Addition of [2.2.2]cryptand, 0.01875 M NaO^{i}Pr instead of KO^{i}Pr

0.0000 M [2.2.2]cryptand		
t (s)	[epoxide]	[product]
0	0.250	0.000
1800	0.246	0.003
3600	0.245	0.007
5400	0.240	0.011
7200	0.236	0.015
9000	0.232	0.019
10800	0.229	0.023
12600	0.224	0.027
14400	0.219	0.031

0.0000 M [2.2.2]cryptand		
t (s)	[epoxide]	[product]
0	0.250	0.000
1800	0.247	0.003
3600	0.245	0.008
5400	0.241	0.012
7200	0.236	0.017
9000	0.231	0.021
10800	0.226	0.026
12600	0.222	0.030
14400	0.217	0.035

0.0187 M [2.2.2]cryptand		
t (s)	[epoxide]	[product]
0	0.250	0.000
1980	0.241	0.009
3780	0.236	0.014
5580	0.228	0.020
7380	0.220	0.027
9180	0.213	0.034
10980	0.206	0.040
12780	0.199	0.046
14580	0.193	0.052

0.0187 M [2.2.2]cryptand		
t (s)	[epoxide]	[product]
0	0.250	0.000
1800	0.242	0.008
3600	0.234	0.015
5400	0.225	0.023
7200	0.218	0.030
9000	0.210	0.037
10800	0.202	0.044
12600	0.194	0.051
14400	0.187	0.057

0.0187 M [2.2.2]cryptand		
t (s)	[epoxide]	[product]
0	0.250	0.000
1860	0.243	0.007
3660	0.234	0.014
5460	0.226	0.022
7260	0.219	0.029
9060	0.211	0.035
10860	0.203	0.042
12660	0.196	0.049
14460	0.189	0.055

0.0375 M [2.2.2]cryptand		
t (s)	[epoxide]	[product]
0	0.250	0.000
1860	0.244	0.005
3660	0.237	0.013
5460	0.227	0.021
7260	0.218	0.029
9060	0.209	0.038
10860	0.199	0.046
12660	0.190	0.055
14460	0.181	0.063

0.0375 M [2.2.2]cryptand		
t (s)	[epoxide]	[product]
0	0.250	0.000
1920	0.245	0.005
3720	0.237	0.012
5520	0.227	0.021
7320	0.219	0.029
9120	0.210	0.036
10920	0.201	0.045
12720	0.193	0.053
14520	0.183	0.061

0.0375 M [2.2.2]cryptand		
t (s)	[epoxide]	[product]
0	0.250	0.000
1860	0.240	0.010
3660	0.230	0.020
5460	0.220	0.029
7260	0.209	0.039
9060	0.200	0.048
10860	0.190	0.056
12660	0.181	0.065
14460	0.172	0.000

0.0750 M [2.2.2]cryptand		
t (s)	[epoxide]	[product]
0	0.250	0.000
1920	0.248	0.002
3720	0.244	0.005
5520	0.239	0.011
7320	0.228	0.020
9120	0.219	0.028
10920	0.212	0.036
12720	0.202	0.044
14520	0.194	0.051

0.0750 M [2.2.2]cryptand		
t (s)	[epoxide]	[product]
0	0.250	0.000
2040	0.242	0.008
3840	0.233	0.015
5640	0.223	0.024
7440	0.215	0.031
9240	0.207	0.039
11040	0.199	0.047
12840	0.191	0.054
14640	0.182	0.062

0.0750 M [2.2.2]cryptand		
t (s)	[epoxide]	[product]
0	0.250	0.000
1980	0.242	0.008
3780	0.233	0.016
5580	0.224	0.024
7380	0.216	0.032
9180	0.208	0.039
10980	0.199	0.047
12780	0.190	0.055
14580	0.182	0.062

Determination of the Rate Constant k_2 from Kinetic Data

In the above section entitled "**Derivation of the Rate Law for Catalytic Epoxide Hydrogenolysis**," the following rate law for epoxide hydrogenolysis was derived:

$$\frac{dP}{dt} = \frac{k_2[Ru]_{total}[H_2][epoxide]}{[H_2] + \frac{1}{K_1}}$$

In a kinetic experiment where epoxide is consumed while $[Ru]_{total}$ and $[H_2]$ are constant, [epoxide] is the only variable:

$$\frac{dP}{dt} = \frac{k_2[Ru]_{total}[H_2]}{[H_2] + \frac{1}{K_1}}[epoxide]$$

Therefore, the pseudo-first-order rate law applies:

$$\ln[epoxide] = -k_{obs}t + \ln[epoxide]_0$$

where:

$$k_{obs} = \frac{k_2[Ru]_{total}[H_2]}{[H_2] + \frac{1}{K_1}}$$

Because $[Ru]_{total}$ and $[H_2]$ are known and constant for each experiment, and K_1 was determined by NMR experiments, we can solve for k_2:

$$k_2 = k_{obs}\frac{[H_2] + \frac{1}{K_1}}{[Ru]_{total}[H_2]}$$

Table S8. Calculation of k_2 from kinetics experiments.

[Ru]	$[epoxide]_0$	$P(H_2)$ (bar)	$[H_2]$	$[KO^iPr]$	k_{obs} (s^{-1})	k_2 ($M^{-1}\cdot s^{-1}$)
0.0050	0.250	20	0.0697	0.0188	7.029E-05	0.01632
0.0050	0.250	20	0.0697	0.0188	6.385E-05	0.01482
0.0050	0.250	20	0.0697	0.0188	7.021E-05	0.01630
0.0050	0.250	20	0.0697	0.0188	6.068E-05	0.01409
0.0025	0.250	20	0.0697	0.0188	2.915E-05	0.01354
0.0038	0.250	20	0.0697	0.0188	4.229E-05	0.01309
0.0063	0.250	20	0.0697	0.0188	8.884E-05	0.01650
0.0075	0.250	20	0.0697	0.0188	1.059E-04	0.01639
0.0050	0.125	20	0.0697	0.0188	7.200E-05	0.01672
0.0050	0.375	20	0.0697	0.0188	6.206E-05	0.01441
0.0050	0.250	10	0.0348	0.0188	5.520E-05	0.01459
0.0050	0.250	30	0.1045	0.0188	6.916E-05	0.01532
0.0050	0.250	30	0.1045	0.0188	7.702E-05	0.01706
0.0050	0.250	30	0.1045	0.0188	7.883E-05	0.01746
0.0050	0.250	20	0.0697	0.0063	5.231E-05	0.01215
0.0050	0.250	20	0.0697	0.0125	5.729E-05	0.01330
0.0050	0.250	20	0.0697	0.0125	6.113E-05	0.01419
0.0050	0.250	20	0.0697	0.0250	7.781E-05	0.01807
					Mean	0.0152
					Std. Dev.	0.0016

Table S8 shows the calculation of k_2 for each independent kinetic experiment in Table S5. The molarity of hydrogen is calculated using the Henry's law constant for hydrogen in isopropyl alcohol at 298.15 K, equal to 0.003483 M/bar.[7] By taking the average and standard deviation, we find a global rate constant k_2 of 0.0152 ± 0.0016 $M^{-1}\cdot s^{-1}$. Using the Eyring equation, we find that $\Delta G^\ddagger$ for reaction 2 is 19.93 ± 0.064 kcal/mol at 298.15 K.

Comparison of (R)-Styrene Oxide Hydrogenolysis by RuCl and $RuPNN^{HEt}$.

The data shown in Figure 5 were collected using the same apparatus and procedure described on page S28 for kinetic studies. Reaction solutions, with a total volume of 10.0 mL, were prepared with 0.050 mmol **RuCl** or **$RuPNN^{HEt}$**, 2.50 mmol (*R*)-styrene oxide (99% e.e.), 0.188 mmol KO^iPr (from a 5% solution in isopropyl alcohol), and 0.50 mmol tetradecane as internal standard. Aliquots were removed at regular intervals and analyzed by GC-FID using the method described above in Table S2. The measured concentrations, as well as the calculated yields, e.e.'s, and branched : linear ratios reported in Figure 5, are shown in Table S9 below.

Table S9. Time Course Data for Hydrogenation of (R)-Styrene Oxide by RuCl vs. $RuPNN^{HEt}$.

RuCl

t (hr)	[(R)-styrene oxide]	[(S)-1-phenylethanol]	[(R)-1-phenylethanol]	2-phenylethanol	% Yield	% e.e.	b : l
0.00	0.25000	0.00000	0.00000	0.00000	0.0		
0.15	0.24348	0.00525	0.00010	0.00064	2.4	96.4	8.4
0.30	0.23262	0.01815	0.00015	0.00177	8.0	98.3	10.4
0.45	0.21816	0.03225	0.00024	0.00293	14.2	98.5	11.1
1.00	0.20219	0.04741	0.00030	0.00418	20.8	98.7	11.4
1.50	0.17333	0.07305	0.00045	0.00665	32.1	98.8	11.1
2.00	0.14807	0.09661	0.00054	0.00866	42.3	98.9	11.2
2.50	0.12614	0.11660	0.00071	0.01033	51.1	98.8	11.4
3.00	0.10768	0.13379	0.00080	0.01184	58.6	98.8	11.4
3.50	0.09217	0.14817	0.00092	0.01311	64.9	98.8	11.4
4.00	0.07831	0.16117	0.00099	0.01409	70.5	98.8	11.5

$RuPNN^{HEt}$

t (hr)	[(R)-styrene oxide]	[(S)-1-phenylethanol]	[(R)-1-phenylethanol]	2-phenylethanol	% Yield	% e.e.	b : l
0.00	0.25000	0.00000	0.00000	0.00000	0.0		
0.15	0.22582	0.01029	0.00820	0.00372	8.9	11.3	5.0
0.30	0.19867	0.02171	0.01942	0.00795	19.6	5.6	5.2
0.45	0.17525	0.03143	0.02926	0.01173	29.0	3.6	5.2
1.00	0.15526	0.03975	0.03766	0.01476	36.9	2.7	5.2
1.50	0.12247	0.05317	0.05148	0.02001	49.9	1.6	5.2
2.00	0.09697	0.06379	0.06231	0.02406	60.1	1.2	5.2
2.50	0.07692	0.07179	0.07067	0.02715	67.8	0.8	5.2
3.00	0.06220	0.07806	0.07713	0.02957	73.9	0.6	5.2
3.50	0.04946	0.08359	0.08273	0.03163	79.2	0.5	5.3
4.00	0.03985	0.08706	0.08643	0.03279	82.5	0.4	5.3

In the above table, "% Yield" is the total yield of phenylethanol isomers, "% e.e." is the enantiomeric excess of the 1-phenylethanol product, and "b : l" is the ratio of branched 1-phenylethanol to linear 2-phenylethanol.

Table S10. Energies calculated by DFT for RuPNN^{HEt}-Catalyzed Dehydrogenation of 2-Propanol

Small Molecules	E(BS2)	G(corr)	SS G(kcal)
H_2	-1.175828688	-0.00151	-736.896913
acetone	-193.1795577	0.055695	-121185.069
2-propanol	-194.3871279	0.081117	-121925.355

Name	E(BS2)	G(corr)	SS G(kcal)	Mass Balance	G(total, kcal)	G(rel)
RuPNN^{HEt}	-1327.012682	0.433732	-832438.34	2-propanol	-954363.6924	1.3
p	-1521.413972	0.532361	-954365.01		-954365.006	0.0
q-TS	-1521.384562	0.532076	-954346.73		-954346.7294	18.3
r	-1521.384944	0.531393	-954347.40		-954347.3981	17.6
s-TS	-1521.382208	0.527700	-954348.00		-954347.9982	17.0
t	-1521.384286	0.530399	-954347.61		-954347.6088	17.4
u	-1520.203857	0.511783	-953618.56	H_2	-954355.4573	9.5
v	-1520.207150	0.513545	-953619.52	H_2	-954356.4183	8.6
w-TS	-1520.202630	0.511123	-953618.20	H_2	-954355.1016	9.9
x	-1520.205612	0.514170	-953618.16	H_2	-954355.0607	9.9
y-TS	-1520.192331	0.512318	-953610.99	H_2	-954347.8893	17.1
z	-1520.204485	0.509772	-953620.22	H_2	-954357.1135	7.9
RuPNN^{HEt}	-1327.012682	0.433732	-832438.34	H_2 + acetone	-954360.3029	4.7

References

(1) Le, L.; Liu, J.; He, T.; Kim, D.; Lindley, E. J.; Cervarich, T. N.; Malek, J. C.; Pham, J.; Buck, M. R.; Chianese, A. R. Structure–Function Relationship in Ester Hydrogenation Catalyzed by Ruthenium CNN-Pincer Complexes. *Organometallics* **2018,** *37*, 3286-3297.

(2) Kim, D.; Le, L.; Drance, M. J.; Jensen, K. H.; Bogdanovski, K.; Cervarich, T. N.; Barnard, M. G.; Pudalov, N. J.; Knapp, S. M. M.; Chianese, A. R. Ester Hydrogenation Catalyzed by CNN-Pincer Complexes of Ruthenium. *Organometallics* **2016,** *35*, 982-989.

(3) He, T.; Buttner, J. C.; Reynolds, E. F.; Pham, J.; Malek, J. C.; Keith, J. M.; Chianese, A. R. Dehydroalkylative Activation of CNN- and PNN-Pincer Ruthenium Catalysts for Ester Hydrogenation. *J. Am. Chem. Soc.* **2019,** *141*, 17404-17413.

(4) Chianese, A. R.; Mo, A.; Lampland, N. L.; Swartz, R. L.; Bremer, P. T. Iridium Complexes of CCC-Pincer N-Heterocyclic Carbene Ligands: Synthesis and Catalytic C-H Functionalization. *Organometallics* **2010,** *29*, 3019-3026.

(5) Chianese, A. R.; Shaner, S. E.; Tendler, J. A.; Pudalov, D. M.; Shopov, D. Y.; Kim, D.; Rogers, S. L.; Mo, A. Iridium Complexes of Bulky CCC-Pincer N-Heterocyclic Carbene Ligands: Steric Control of Coordination Number and Catalytic Alkene Isomerization. *Organometallics* **2012,** *31*, 7359-7367.

(6) Schaus, S. E.; Brandes, B. D.; Larrow, J. F.; Tokunaga, M.; Hansen, K. B.; Gould, A. E.; Furrow, M. E.; Jacobsen, E. N. Highly Selective Hydrolytic Kinetic Resolution of Terminal Epoxides Catalyzed by Chiral (Salen)Co^{iii} Complexes. Practical Synthesis of Enantioenriched Terminal Epoxides and 1,2-Diols. *J. Am. Chem. Soc.* **2002,** *124*, 1307-1315.

(7) Brunner, E. Solubility of Hydrogen in Alcohols. *Ber. Uunsenges. Phys. Chem.* **1979,** *83*, 715-721.
