# Supplementary figures and images for: Highly Enantiomerically Enriched Secondary Alcohols via Epoxide Hydrogenolysis
Source: Organometallics. 2024 Jun 17;43(13):1490–501. doi: 10.1021/acs.organomet.4c00214 (PMC11234370; doi:10.1021/acs.organomet.4c00214)

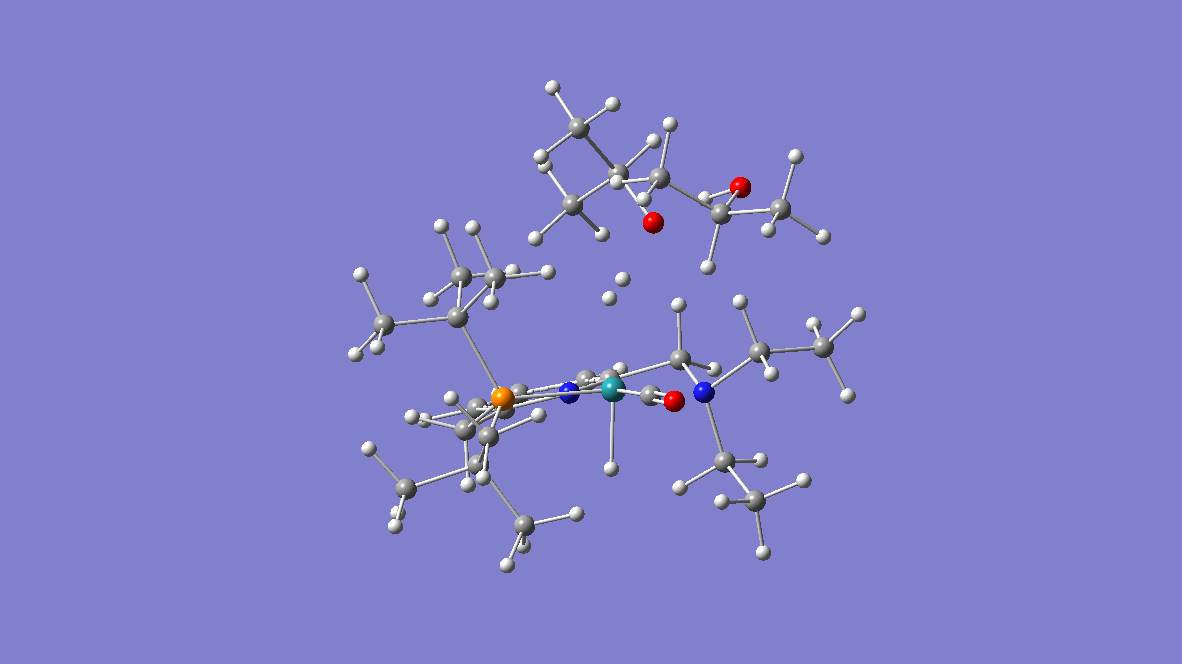

Supplement: Supplementary file 3 — om4c00214_si_003.zip [file om4c00214_si_003.zip › c-TS.gif]

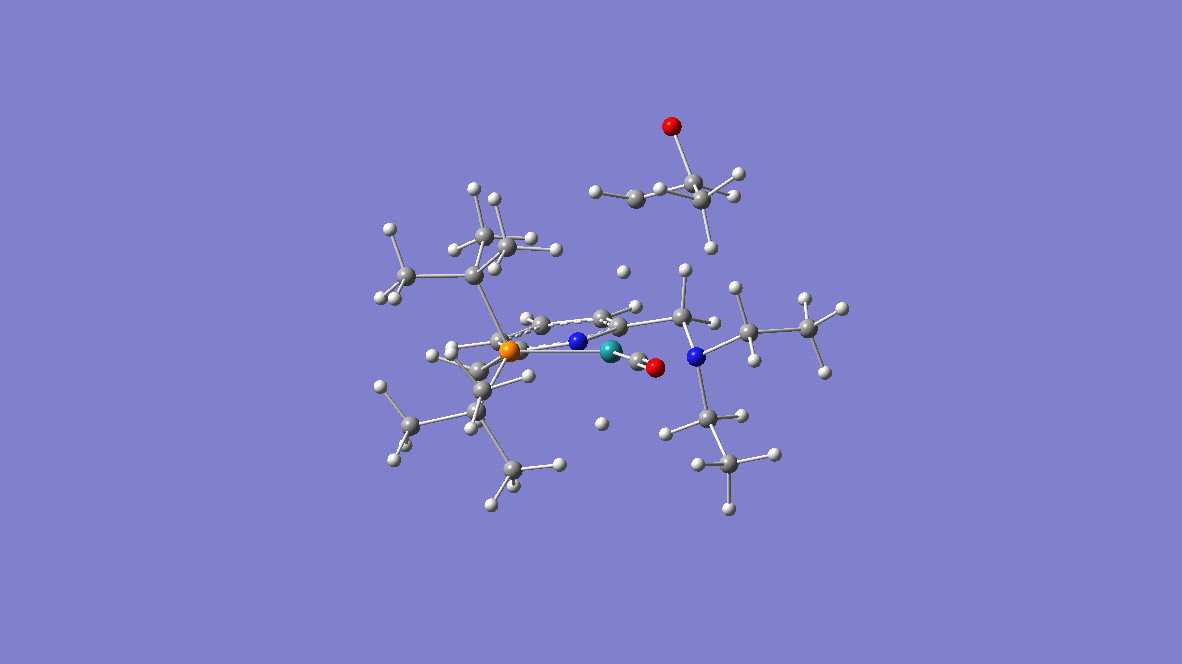

Supplement: Supplementary file 3 — om4c00214_si_003.zip [file om4c00214_si_003.zip › e-TS.gif]

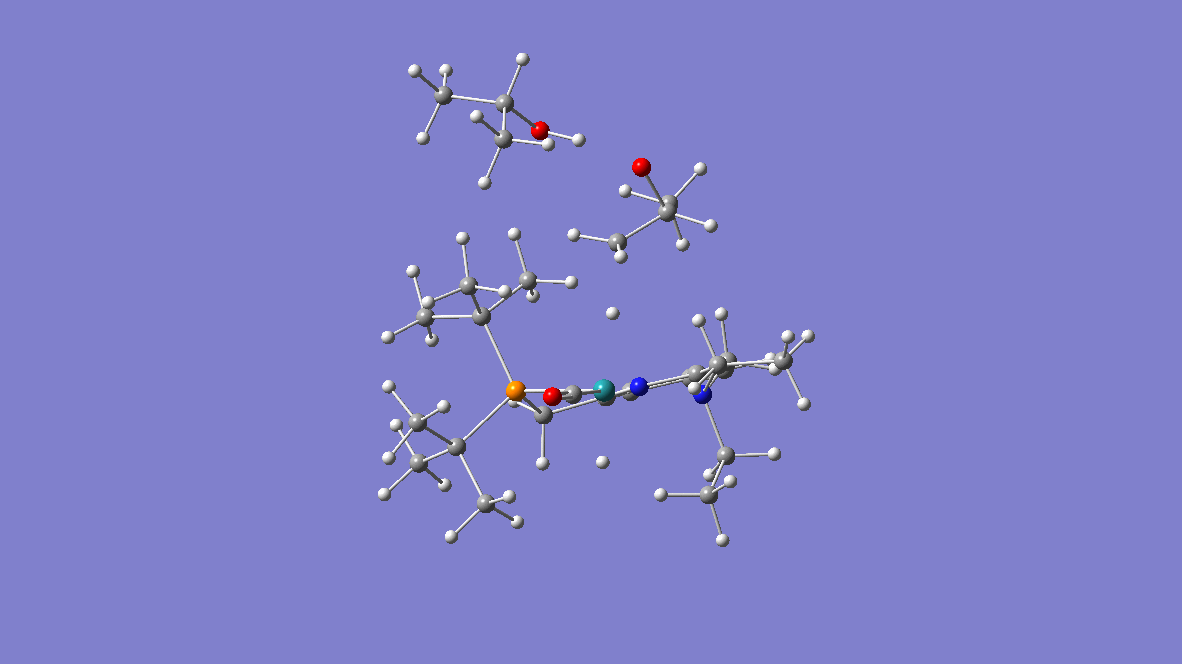

Supplement: Supplementary file 3 — om4c00214_si_003.zip [file om4c00214_si_003.zip › h-TS.gif]

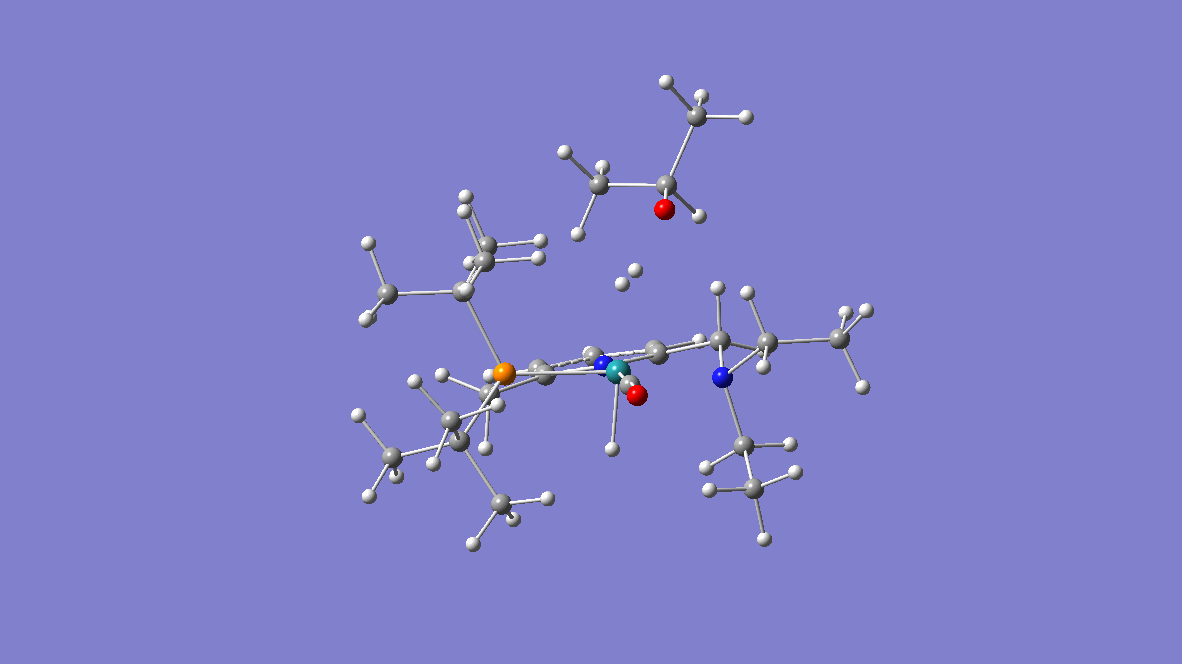

Supplement: Supplementary file 3 — om4c00214_si_003.zip [file om4c00214_si_003.zip › k-TS.gif]

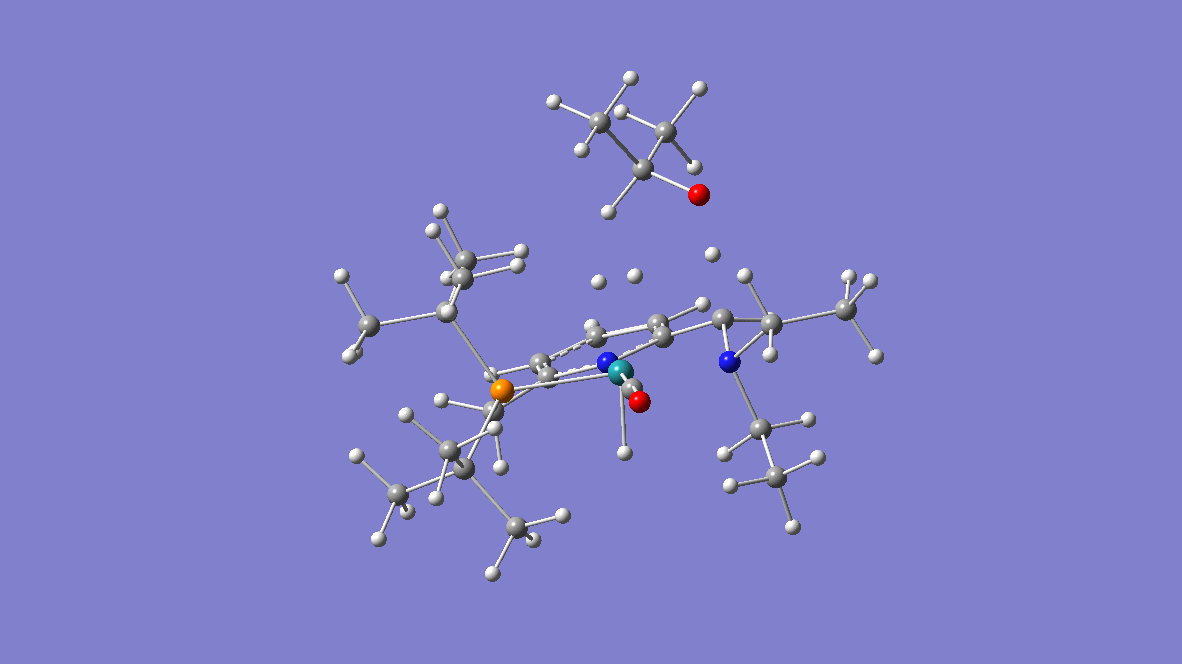

Supplement: Supplementary file 3 — om4c00214_si_003.zip [file om4c00214_si_003.zip › m-TS.gif]

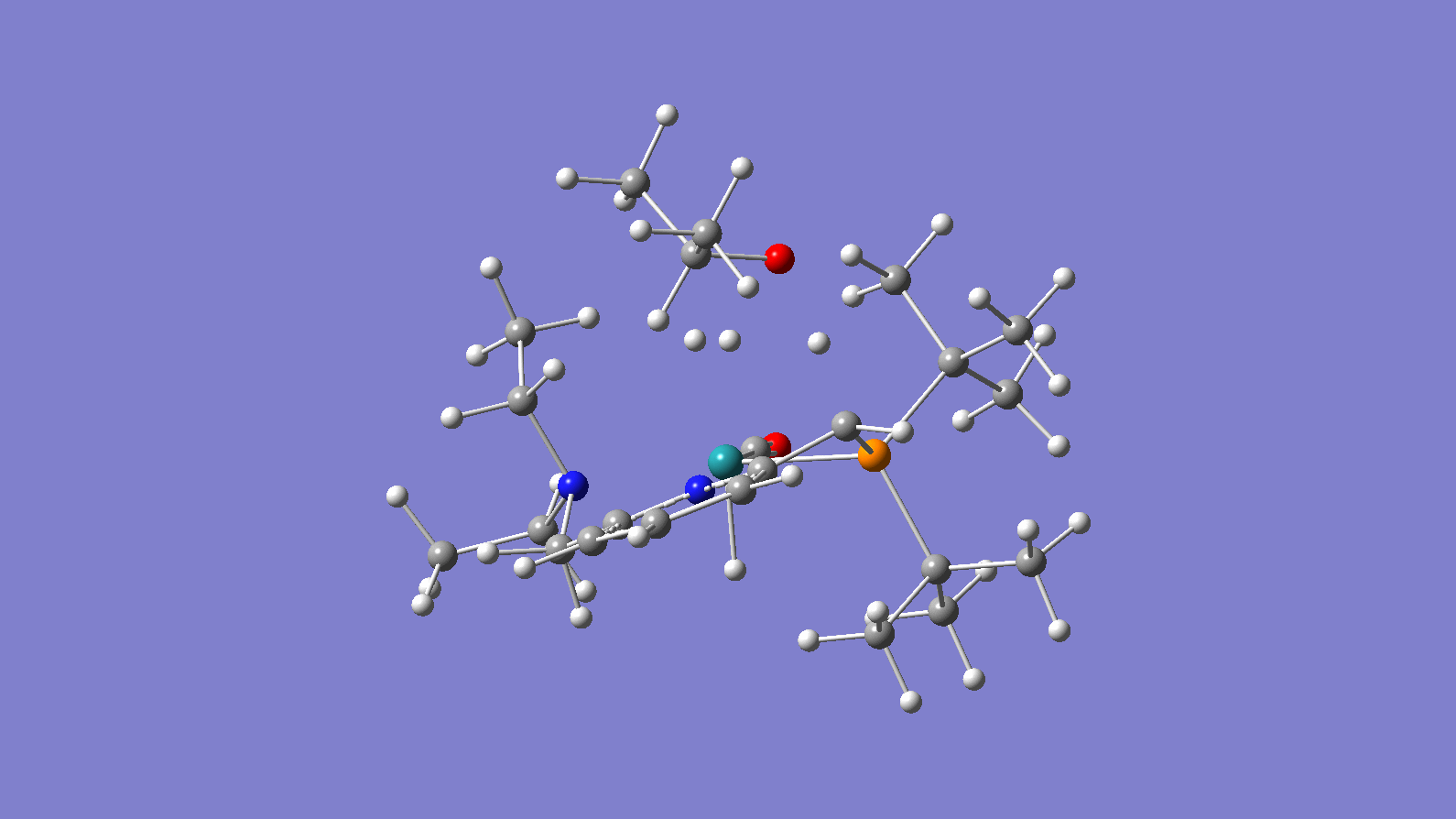

Supplement: Supplementary file 3 — om4c00214_si_003.zip [file om4c00214_si_003.zip › o-TS.gif]

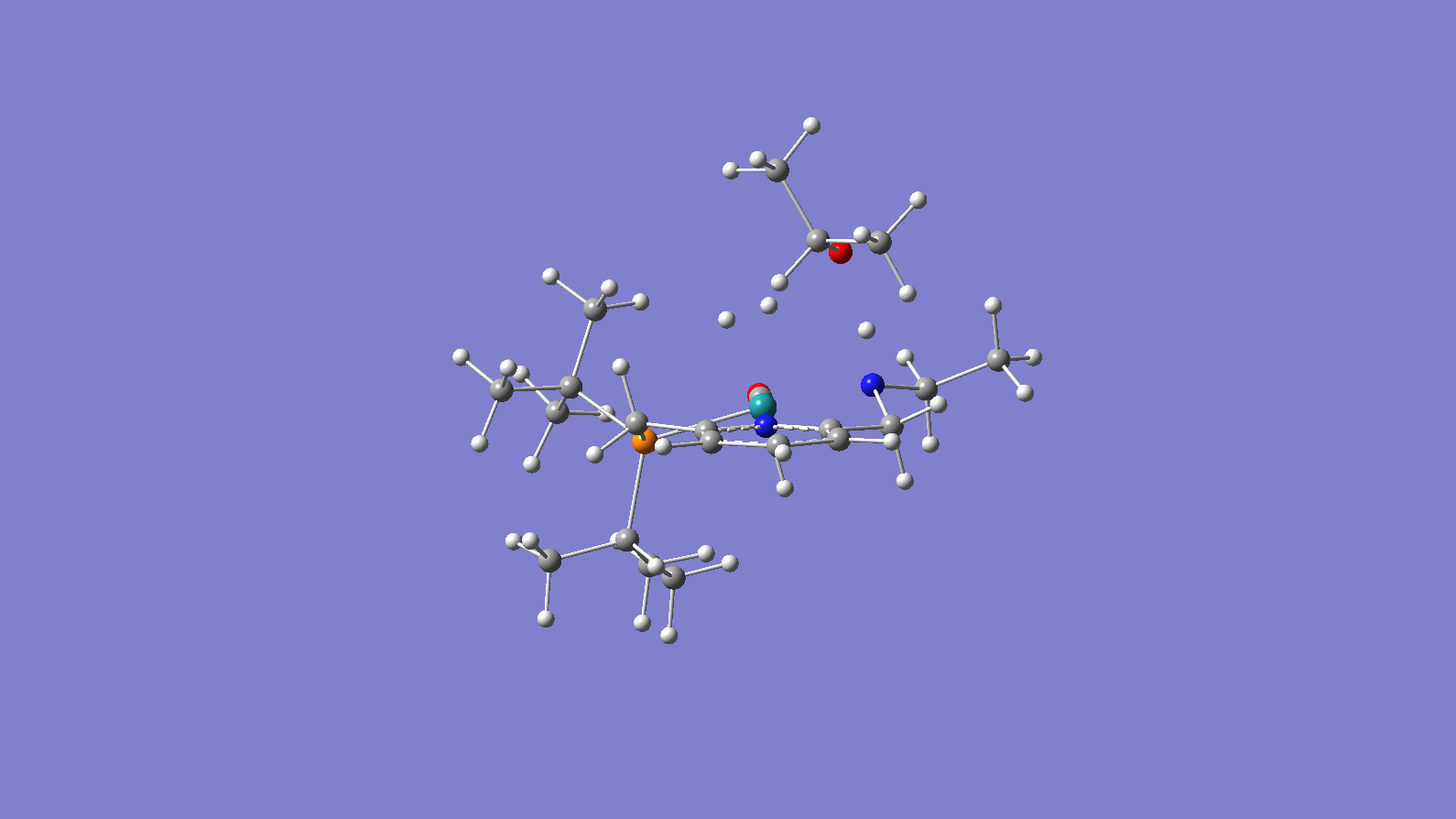

Supplement: Supplementary file 3 — om4c00214_si_003.zip [file om4c00214_si_003.zip › q-TS.gif]

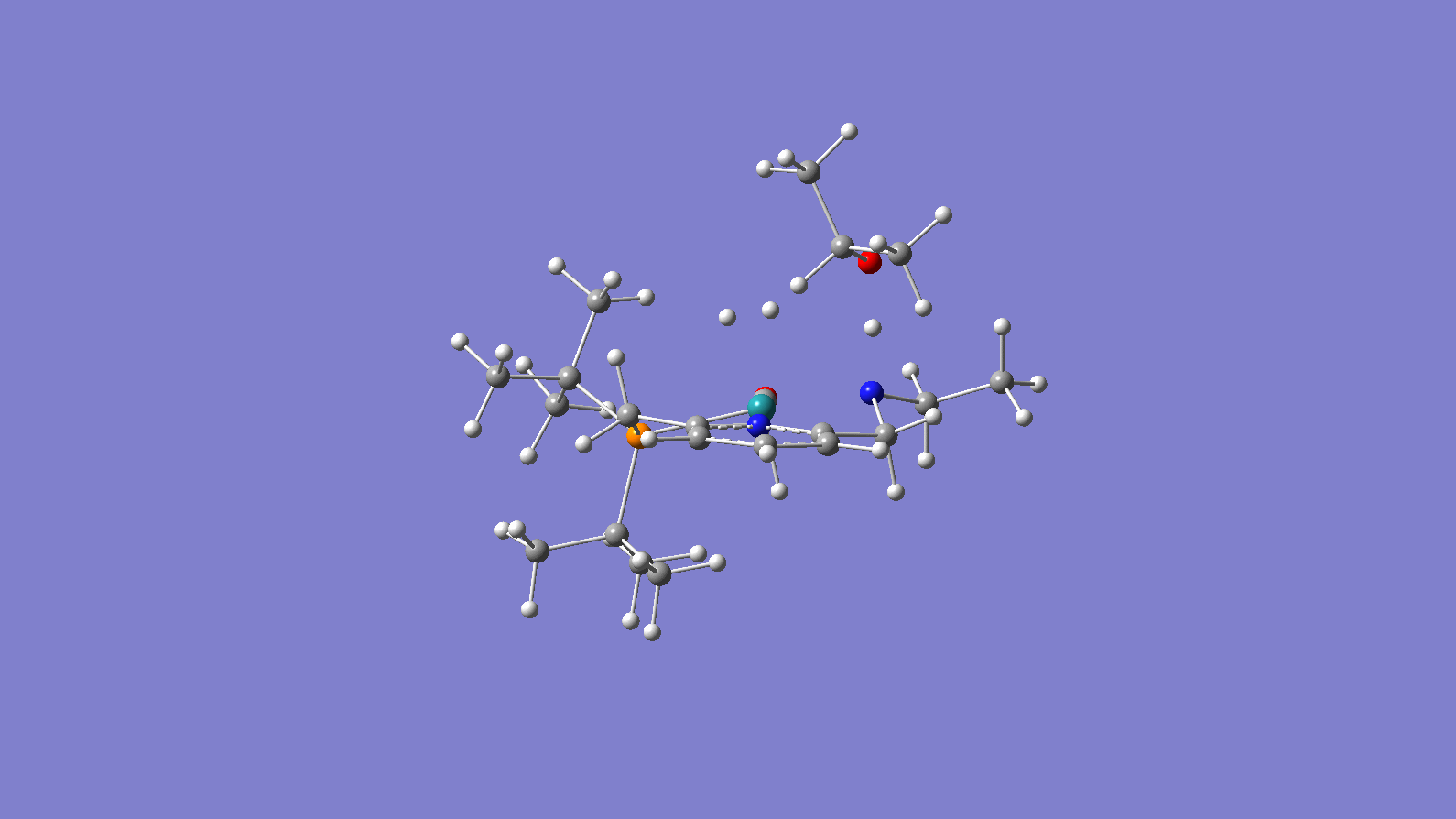

Supplement: Supplementary file 3 — om4c00214_si_003.zip [file om4c00214_si_003.zip › s-TS.gif]

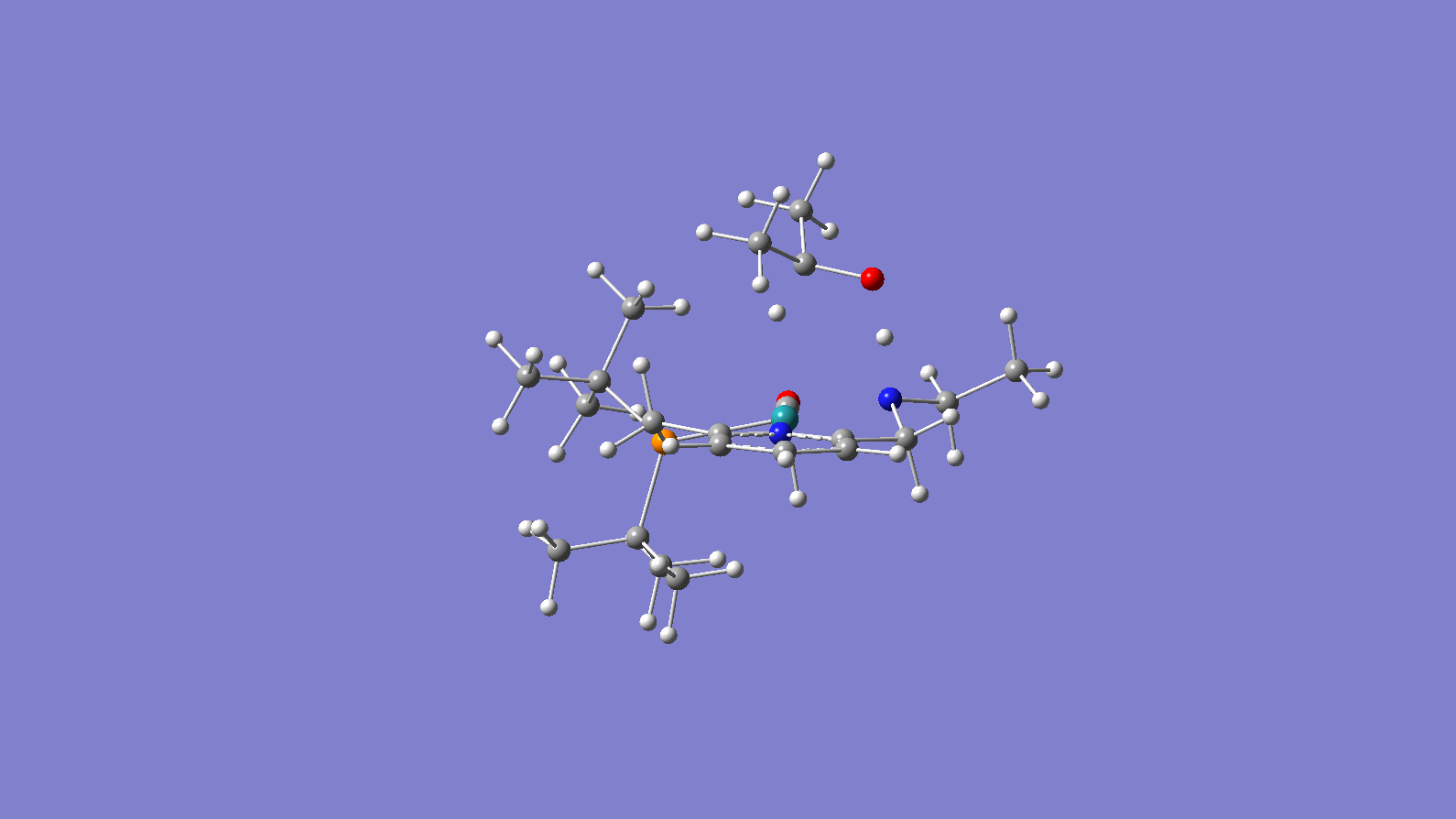

Supplement: Supplementary file 3 — om4c00214_si_003.zip [file om4c00214_si_003.zip › w-TS.gif]

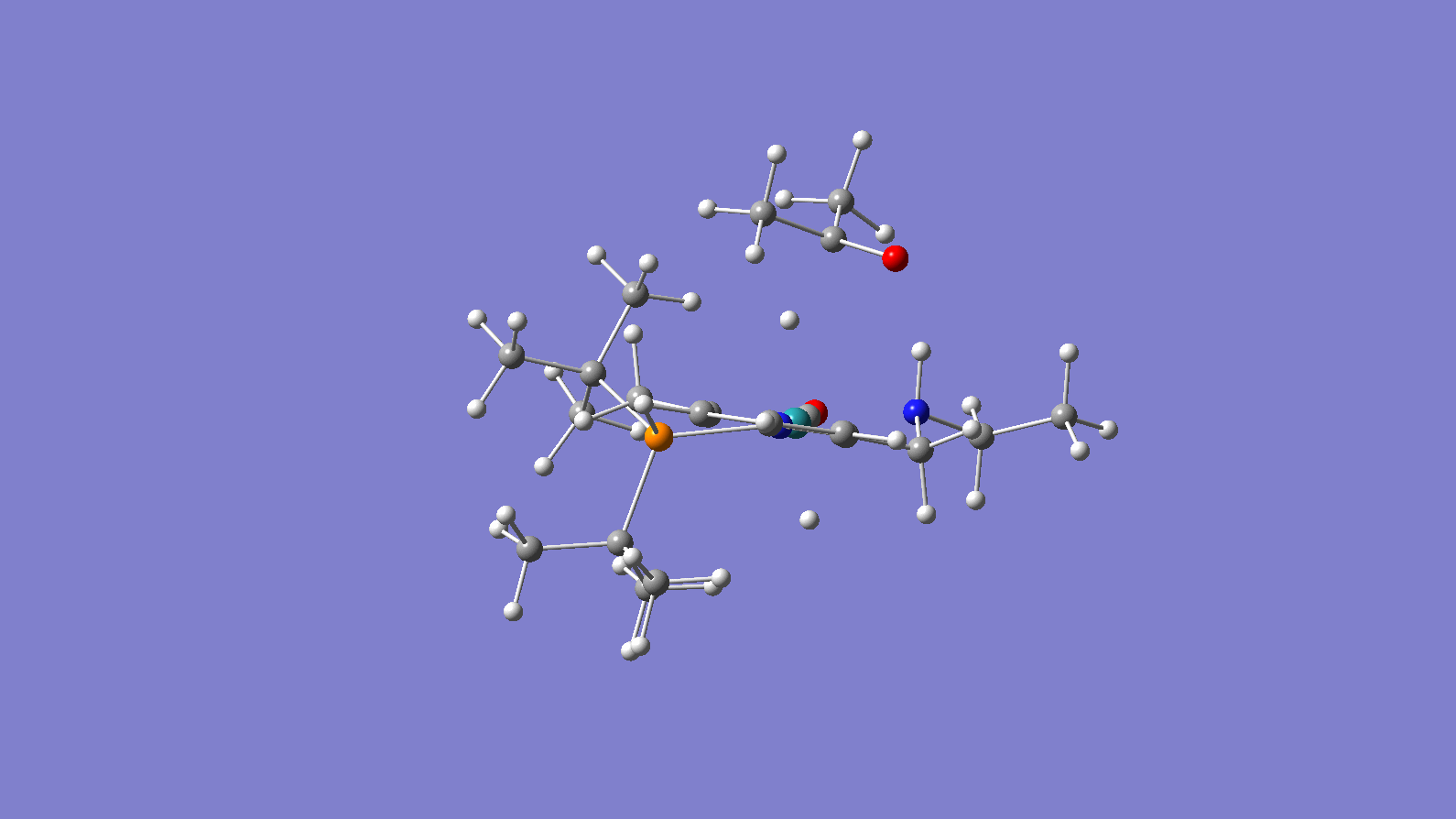

Supplement: Supplementary file 3 — om4c00214_si_003.zip [file om4c00214_si_003.zip › y-TS.gif]
